# Conversion of Bestmann Ylide into Carbophosphinocarbene

**DOI:** 10.1002/anie.202501955

**Published:** 2025-04-07

**Authors:** Libo Xiang, Junyi Wang, Niclas Knoblauch, Alexander Matler, Qing Ye

**Affiliations:** ^1^ Institute for Inorganic Chemistry Institute for Sustainable Chemistry & Catalysis with Boron Julius‐Maximilians‐Universität Würzburg, Am Hubland Würzburg 97074 Germany; ^2^ Department of Chemistry Southern University of Science and Technology 1088 Xueyuan Blvd., Xili Nanshan District Shenzhen 518055 China

**Keywords:** Borirane, Carbone, Carborane, Click, Ylide

## Abstract

The *ortho*‐carboranyl carbophosphinocarbene (CPC) has been synthesized through a click‐type reaction between the super strained carborane‐fused borirane 1,2‐BN(SiMe_3_)_2_–1,2‐C_2_B_10_H_10_ and Bestmann ylide Ph_3_PCCO. The [Cl(CO)₂Ir‐CPC] and [Cl_3_Ga‐CPC] complexes were synthesized, allowing the measurement of their Tolman electronic parameter (TEP) and the sum of Cl─Ga─Cl bond angles (∑ClGaCl), respectively. These data highlight their remarkable electron‐donating ability. Further insights into its electron‐donating properties were gained through theoretical calculations, including analysis of frontier orbitals and proton affinity (PA). Preliminary reactivity investigations demonstrate that the new CPC readily forms adducts with boranes and can effectively stabilize borenium cations. Its strong nucleophilicity enables reactions with carbon dioxide, while its exceptional Brønsted basicity allows it to deprotonate imidazolium to generate carbene species.

## Graphical abstract

The *ortho*‐carboranyl carbophosphinocarbene(CPC) was synthesized via a click‐type reaction. Experimental and computational studies reveal its remarkable electron‐donating ability. Reactivity studies demonstrate its versatility as a stabilizing ligand, a strong nucleophile for CO₂ activation, and a potent Brønsted base for imidazolium deprotonation.

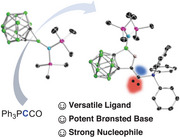

As for carbon‐based *σ*‐donor ligands, singlet carbenes of the general formula R_2_C: undoubtedly hold a prominent position. Since the isolation of the first stable acyclic carbene by Bertrand^[^ ^1^ ^]^ in 1988 and the first stable cyclic carbene by Arduengo^[^ ^2^ ^]^ in 1991, isolable carbene ligands such as N‐heterocyclic carbenes (NHCs) have played an important role across numerous research areas.^[^ ^3^, ^4^, ^5^, ^6^, ^7^ ^]^ This benefits from their strong *σ*‐donating abilities, excellent metal binding affinity, and ease of synthesis.

In recent years, another class of neutral carbon‐based *σ*‐donor ligands, i.e., carbones of the general formula L → C ← L (where L is an electron donor, such as phosphines and NHCs), has gained much attention thanks to a renewed understanding of the bonding in these compounds.^[^ ^8^, ^9^, ^10^, ^11^, ^12^ ^]^ In fact, the synthesis of carbone has a history of over 60 years. In 1961, Ramirez and coworkers reported the first synthesis of carbodiphosphorane (CDP) Ph_3_PCPPh_3_,^[^ ^13^ ^]^ while its single crystal structure was reported 10 years later.^[^ ^14^ ^]^ Depending on the crystallization conditions, the central carbon of Ph_3_PCPPh_3_ exhibited a bent^[^ ^14^, ^15^ ^]^ or linear^[^ ^16^ ^]^ structure, with the PCP angle ranging between 130.1° and 180.0°. Although there have been a few reports on CDP transition metal complexes^[^ ^17^, ^18^, ^19^, ^20^, ^21^, ^22^, ^23^, ^24^, ^25^, ^26^, ^27^, ^28^, ^29^ ^]^ in the following years, the understanding of the electronic structure of CDP itself and its electron‐donating properties as a ligand remains limited. In 2006, Neumüller, Petz, Frenking, and coworkers demonstrated through theoretical calculations and experiments that Ph_3_PCPPh_3_ can indeed be regarded as a phosphine complex of carbon(0), with the central carbon having two available lone pairs of electrons and exhibiting higher proton affinity than NHCs.^[^ ^30^, ^31^, ^32^ ^]^ Inspired and motivated by this reinterpretation of CDP, carbone chemistry began to develop rapidly nearly 50 years after its first synthesis. This includes the expansion of the scope of carbones to electron‐rich allenes named carbodicarbenes (CDC),^[^ ^33^, ^34^, ^35^ ^]^ as well as the applications in constructing novel transition metal complexes^[^ ^36^, ^37^, ^38^, ^39^, ^40^, ^41^, ^42^, ^43^ ^]^ and catalysts,^[^ ^44^, ^45^, ^46^, ^47^, ^48^, ^49^ ^]^ and stabilizing main‐group element compounds.^[^ ^50^, ^51^, ^52^, ^53^, ^54^ ^]^ Carbophosphinocarbene (CPC) in the form of R_3_P → C ← CR_2_ (**I**) or R_3_P → C = CR_2_ (**II**) can be regarded as a class of carbene and phosphine mixed carbones (Figure 1). It was first synthesized by Bestmann and coworkers in 1973 (**A** in Figure 1) and was referred to as vinylidenephosphorane at the time.^[^ ^55^, ^56^ ^]^ The same group published the single crystal structure a year later, showing that it exhibited a bent structure similar to CDP.^[^ ^56^ ^]^ In 1977, they reported the first synthesis of a cyclic CPC.^[^ ^57^ ^]^ Similar to CDP, CPC chemistry also experienced a roughly 30‐year hiatus, until 2008, when the Bertrand group conducted an in‐depth study on cyclic CPC (**B** in Figure 1), demonstrating that **B** has a strong *σ*‐donating ability and readily coordinates with [Rh(CO)_2_Cl] to yield the first CPC‐transition metal complexes.^[^ ^58^ ^]^ A year later, the Fürstner group proposed viewing CPCs as carbene and phosphine mixed complexes of carbon.^[^ ^59^ ^]^ In addition to synthesizing AuCl complexes of **C** and **D**, **A** was also shown to be able to provide two electron lone pairs to form a digold complex, supporting the extreme structural formula **I**, where the ability of the C‐center to donate two pairs of electrons is highlighted. In 2015, Ong and co‐workers successfully synthesized the NHC and phosphine mixed carbene **E**, which displays a higher *σ*‐donating ability than NHC and can serve as an organocatalyst for the reductive N‐methylation of amines with CO_2_ in the presence of borane.^[^ ^60^, ^61^ ^]^

**Figure 1:**
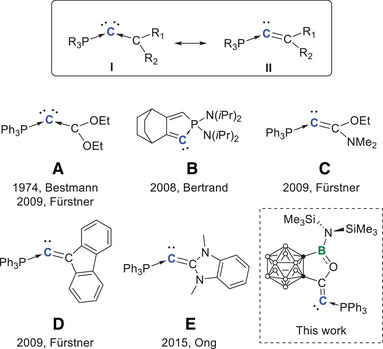
The extreme structural formula I and II of CPC, the reported examples of CPC, **A–E**, and the carboranyl CPC in this work. The assignment of the extreme structure for **A**–**E** and the carboranyl CPC in this work is based on whether dimetal complexes have been attained.

Ketenylidenetriphenylphosphorane (Bestmann ylide) was first synthesized and single crystal structurally characterized in 1966.^[^ ^62^, ^63^ ^]^ With its readiness to participate in chemical transformations, such as cycloaddition reactions,^[^ ^64^ ^]^ and those based on its electrophilicity at the carbonyl position,^[^ ^62^, ^63^, ^64^, ^65^, ^66^, ^67^ ^]^ it has proven to be a versatile reagent for organic synthesis and is now commercially available. On the other hand, although Bestmann ylide Ph_3_PCCO could also be regarded as a phosphine and carbonyl mixed carbone, studies have shown that it exhibits indeed very limited carbone character.^[^ ^59^ ^]^ Instead, it is best known as a Lewis base for its ditopicity, meaning both oxygen and carbon have comparable coordination abilities.^[^ ^67^, ^68^ ^]^ For instance, there is no selectivity when Bestmann ylide forms adducts with Lewis acidic boranes that lack steric hindrance.^[^ ^68^ ^]^

Our research group has recently developed a class of noncoordinated carborane‐fused boriranes,^[^ ^69^, ^70^, ^71^, ^72^, ^73^, ^74^, ^75^ ^]^ which has demonstrated its versatility in click‐type ring‐expansion reactions.^[^ ^69^, ^75^ ^]^ We are curious whether similar reactions can be achieved with Bestmann ylide, thus converting it into a carboranyl CPC in a click manner. More interestingly, in contrast to previously reported CPCs, the CPC constructed using this method features an *ortho*‐carboranyl‐substituted boryl backbone. The strong inductive effect of the *o*‐carborane at the *C*‐positions^[^ ^76^, ^77^, ^78^, ^79^, ^80^ ^]^ should result in considerable Lewis acidity at the boryl center. Thus, if the carboranyl CPC exists as a monomer without undergoing intermolecular oligomerization, it could serve as a unique Lewis acid–base bifunctional molecule. Herein, we present our validation of this hypothesis, including click‐type synthesis, the single‐crystal structure of its monomeric form, and its unique reactivity, wherein both the CPC and boryl sites participate in the reactions.

The *o*‐carborane‐fused aminoborirane **1** was reacted with the Bestmann ylide Ph_3_PCCO in toluene. The reaction was monitored by multinuclear NMR spectroscopy (Scheme 1). The ^11^B‐NMR spectrum showed a complete and selective conversion of **1** (23.9 ppm) into a new boron‐containing species showing a resonance at 34.6 ppm. Meanwhile, the ^31^P NMR spectrum displayed a singlet at −0.4 ppm that is slightly up‐field shifted compared to that of Ph_3_PCCO (3.1 ppm), implying that the electronic environment of the phosphorus center is not much affected. After workup, the crude product was recrystallized from a saturated Et_2_O solution at −30 °C to afford **2** as a yellow crystalline solid with a decent yield of 91%. Single crystal X‐ray diffraction analysis confirmed the atom connectivity of **2**, indicating that the carbonyl group of Bestmann ylide was inserted in a side‐on manner into the highly strained C_2_B three‐membered ring (Figure 2).^[^ ^81^ ^]^ As a consequence, the carbonyl group was converted into a (carboranyl)(oxy)carbene, and **2** can be considered as a (carboranyl)(oxy)carbene/phosphine‐mixed carbone (i.e., CPC). Like the other reported CPCs, **2** featured a bent geometry. The C2─C1─P bond angle of 117.94(18)° is smaller than that of the other reported acyclic CPCs (**A** 125.6(8)°, **E** 143.04(10)°), yet larger than that of the only cyclic CPC (**B** 99.94(11)°). The C1─C2 bond length (1.311(3) Å) is comparable to that of **A** (1.314(10) Å) and **E** (1.3378(17) Å), slightly smaller than that of **B** (1.358(2) Å). The P─C1 bond length (1.733(2) Å) lies in the range expected for a P → C dative bond (**A** 1.682(4) Å, **B** 1. 783(15) Å, **E** 1.6435(12) Å).

**Scheme 1:**
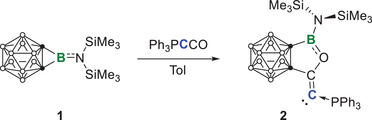
Synthesis of **2**.

**Figure 2:**
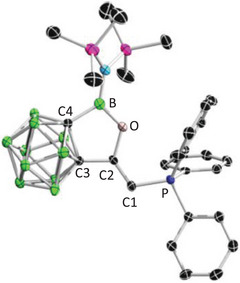
Single crystal structures of **2**. Thermal ellipsoids are drawn at 50% probability level. The hydrogen atoms have been removed for clarity. Selected bond lengths (Å) and angles (°): C1─C2 1.311(3), C2─C3 1.514(3), C3─C4 1.648(3), C1─P 1.733(2), B─C4 1.605(4), B─O 1.384(3), O─B─C4 107.7(2), P─C1─C2 117.94(18).

The Tolman electronic parameter (TEP) is a useful scale for the assessment of the electronic properties of ligands.^[^ ^82^, ^83^, ^84^ ^]^ Strong electron‐donating ligands increase the electron density on the metal center. This will strengthen the π back‐donation from the metal to the CO ligands, thus lowering the CO stretching frequency and leading to smaller TEP values. As such, we set out to synthesize the carbonyl model complex **4**. To this end, cyclooctadiene iridium(I) chloride dimer^[^ ^85^ ^]^ was chosen as a precursor to react with **2** at room temperature for 4 h to afford **3**, which has been a single crystal structurally characterized (Figure S66).

The corresponding dicarbonyl complex **4** was synthesized by bubbling carbon monoxide (CO) through a dichloromethane solution of **3** (Scheme 2). After evaporating all volatiles, the remaining solid was washed with hexane and recrystallized from toluene, yielding the product as an analytically pure yellow powder. The ^31^P NMR spectrum of **4** showed a slightly downfield shifted singlet at 22.3 ppm compared to **3** (18.4 ppm). Yellow crystals suitable for X‐ray diffraction were obtained by slow evaporation of a saturated toluene solution at room temperature. Single crystal X‐ray diffraction analysis unambiguously revealed a square‐planar iridium (I) complex **4** bearing two *cis*‐orientated carbonyl ligands (Figure 3, left). The IR spectrum indicated the CO stretching frequencies at ṽ = 2050 and 1967 cm^−1^, corresponding to a TEP value of 2037.2 cm^−1^ (see SI for details). Based on this, **2** should be less electron donating compared to other CPCs, such as **D** (2034.2 cm^−1^), **B** (2033.8 cm^−1^), and **A** (2025.4 cm^−1^). This could be attributed to the strong inductive effect of the *o*‐carboranyl group^[^ ^76^, ^77^, ^78^, ^79^, ^80^ ^]^ and the oxy group, as well as the presence of a π‐acidic boryl group, which, respectively, weaken the *σ*‐ and π‐donating abilities of the carbone center. Nonetheless, *o*‐carboranyl CPC **2** is still a stronger electron donor compared with classical phosphine‐ and carbene‐ligands such as PEt_3_ (2061.7 cm^−1^), NHCs (2049.6–2054.1 cm^−1^; IMe 2054.1 cm^−1^), and cyclic (alkyl)(amino)carbenes (cAACs, 2046.3–2050.0 cm^−1^; ^Me^cAAC 2050.0 cm^−1^).^[^ ^86^, ^87^, ^88^ ^]^ See Figure 4 and Table 1 for an overview of the TEP values.

**Scheme 2:**
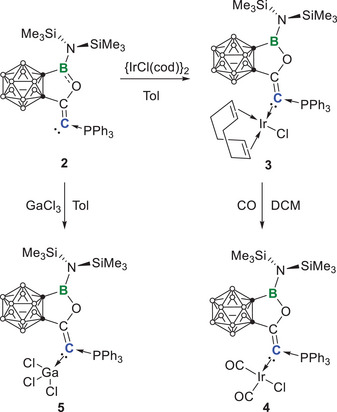
Synthesis of **3**–**5**.

**Figure 3:**
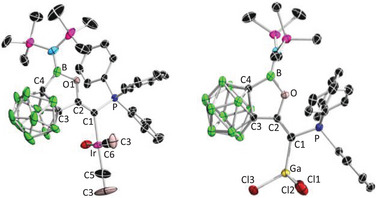
Single crystal structures of **4** (left) and **5** (right). Thermal ellipsoids are drawn at 50% probability level. The hydrogen atoms have been removed for clarity. Selected bond lengths (Å) and angles (°): For **4**: C1─C2 1.327(7), C2─C3 1.499(7), C3─C4 1.639(8), C1─P 1.781(5), B─C4 1.611(9), B─O 1.398(7), O─B─C4 106.1(5), P─C1─C2 117.2(4); For **5**: C1─C2 1.356(4), C2─C3 1.501(4), C3─C4 1.651(4), C1─P 1.808(3), B─C4 1.595(4), B─O 1.408(4), C1─Ga 2.019(3), O─B─C4 106.3(2), P─C1─C2 111.4(2), Cl1─Ga─Cl2 112.54(4), Cl2─Ga─Cl3 102.67(3), Cl3─Ga─Cl1 103.51(3).

**Figure 4:**
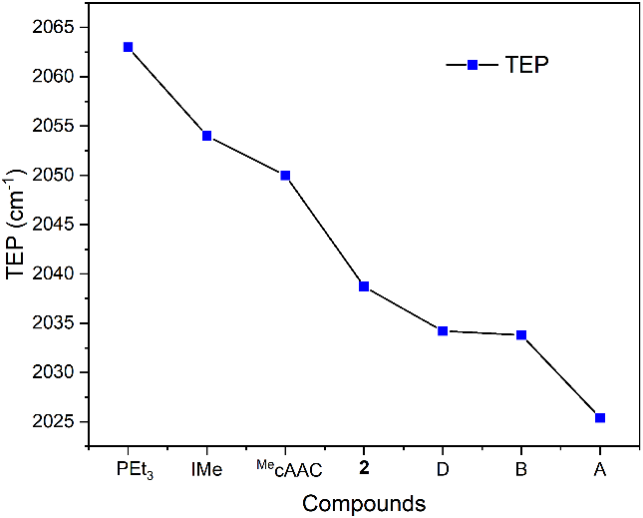
Results of TEP.

**Table 1:** TEP, PA, Sum of Cl─Ga─Cl Angles in L·GaCl_3_ and percent buried volume of L in L·GaCl_3_ (%Vbur) (IMe: 4,5‐dimethyl‐imidazol‐2‐ylidene; MecAAC: 1‐(2,6‐diisopropylphenyl)‐3,3‐dimethyl‐4,5‐dihydro‐1H‐imidazol‐2‐ylidene).

	TEP (cm^−1^)	1^st^PA (kcal mol^−1^)	2^nd^PA (kcal mol^−1^)	Σ_ClGaCl_ exp. (deg)	Σ_ClGaCl_ calcd. (deg)	%V_bur_
PEt_3_	2061.7	231.2	−	330.9(6)	343.9	27.4
Ime	2054.1	261.5	−	325.3(6)	333.9	26.8
IMes	2049.5	271.2	−	325.4(5)	333.9	33.9
^Me^cAAC	2050.0	271.8	−	321.5(9)	333.8	38.0
Ph_3_PCCO	−	247.9	123.4	−	339.6	31.4
**2**	2037.2	273.9	145.9	318.7(3)	330.4°	41.4
**A**	2025.4	285.2	156.3	319.7(1)	328.7	37.1
**B**	2033.8	289.9	138.3	−	331.4	36.4
**C**	−	283.8	170.4	315.9(3)	326.8	39.0
**D**	2034.2	279.9	153.8	−	327.1	38.2
**E**	−	281.1	181.9	320.0(2)	328.9	37.2

Another method to evaluate the electronic properties of ligands involves measuring the sum of the Cl─Ga─Cl bond angles (∑ClGaCl) in [Cl_3_Ga‐L] complexes as an indicator of the degree of pyramidalization of the Ga center, which reflects the donor strength of various ligands, including phosphines, carbenes (NHC/CAAC), and several CDC/CDP ligands.^[^ ^86^ ^]^ The reason is that stronger *σ*‐donors enhance the s‐character of the vacant orbital on Ga that interacts with the ligand, thereby increasing the p‐character of the Ga─Cl bonding, which in turn compresses the Cl─Ga─Cl angles. ∑ClGaCl generally correlates with TEP. However, it should be noted that ∑ClGaCl indicator may be more sensitive to multiple other factors including noncovalent interactions, crystal forces, and topology.^[^ ^86^, ^87^, ^88^ ^]^ We synthesized and structurally characterized the *ortho*‐carboranyl CPC **2**‐ligated gallium complex **5**, indicating a ∑ClGaCl of 318.7(3)°. This value suggests the same trend among PEt₃ (330.9(6)°) < IMe (325.3(6)°) < ^Me^cAAC (321.5(9)°) < **2** as that according to the TEP results. Moreover, since the experimental TEP values of **C** and **E** are unavailable, the ∑ClGaCl values allow for a comparison of the electron‐donating ability among **2**, **E,** and **C**, suggesting that **2** is less electron donating than **C** but more electron donating than **E** (Figure 5). However, it should be noted that the ∑ClGaCl and the TEP results are conflicting regarding the relative electron‐donating abilities of **2** and **A**. Therefore, we should acknowledge that, although ∑ClGaCl of **A** (319.7 (1)°) and **E** (320.0(2)°) are larger than that of **2** (318.7(3)°), this does not necessarily mean that the electron‐donating ability of **2** is greater than **A** and **E**. For instance, the detailed examination of **E** in the solid state revealed a noncovalent interaction between one of the electronegative chloride atoms and one of the flanking NHCs, which, to some extent, increased the Cl─Ga─Cl angle.^[^ ^60^, ^61^ ^]^

To better understand the electronic properties and donating ability of **2**, DFT calculations were performed. The HOMO in **2** is primarily localized on the carbon center, while the HOMO‐1 represents a C─C π orbital. By contrast, the HOMO and HOMO‐1 of Ph_3_PCCO are closely degenerate, both reflecting significant back‐bonding from carbon to CO. The HOMO of **2** is slightly higher in energy than that of Ph_3_PCCO (Figure 6). Orbital localization analysis further reveals that the *σ* orbital in compound **2** is more localized on the carbon center (66.2%), with minimal distribution on the neighboring carbon atom (7.5%). Additionally, the Wiberg bond indices of the C─C (1.954) and C─P (1.223) bonds in Ph₃PCCO are both higher than those of **2** (Table S11, C─C 1.887, C─P 1.193), indicating a stronger π‐interaction in Ph_3_PCCO. The gas‐phase structures of **5** and (Ph_3_P)(OC)C→GaCl₃ were optimized to reveal that the *σ* orbital in **2** is more localized on the carbon comparison. Although the gas‐phase structure is less constrained than the solid‐state structure, usually resulting in larger Cl─Ga─Cl bond angles, ∑ClGaCl of **5** (Table 1 330.4°) is noticeably smaller than that of (Ph_3_P)(OC)C → GaCl₃ (339.6°). Moreover, we calculated both the first and second proton affinities (PAs) of carbenes and CPCs to examine their *σ*‐ and π‐electron donating abilities (Table 1).

**Figure 5:**
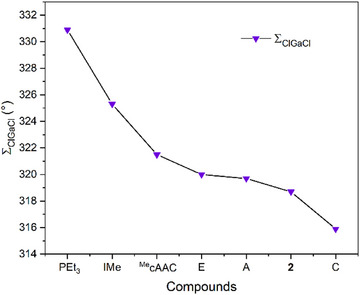
Results of ∑ClGaCl.

**Figure 6:**
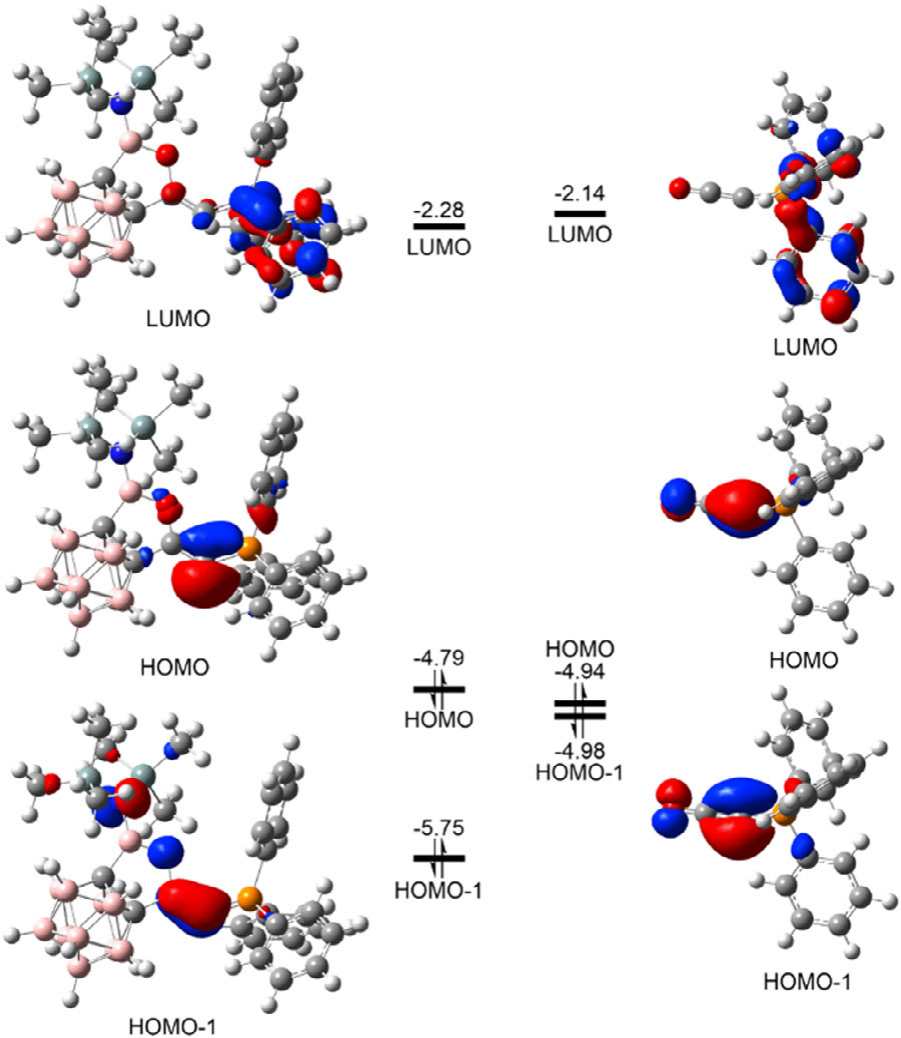
The frontier molecular orbital diagrams and energies (eV) of compound **2** (left) and Ph_3_PCCO (right). Isovalue = 0.05.

The first PA of **2** is notably higher than that of PEt_3_ and Ph_3_PCCO, slightly higher than that of IMe, IMes, and ^Me^cAAC, yet somewhat lower than that of **A**‐**E**. Meanwhile, the second PA of **2** exceeds those of Ph₃PCCO and **B**, but remains lower than those of other CPCs supported by π‐electron donating ligands. In addition, we obtained the ∑ClGaCl values from the optimized structures of the base–GaCl₃ complexes (Table 1), which generally match the trend of reported experimental data. Overall, the ∑ClGaCl values align well with the first PA results. Compound **2** has a smaller ∑ClGaCl than PEt₃, carbenes, and Ph₃PCCO, but a larger ∑ClGaCl than other CPCs except **B**. It should be mentioned that steric hindrance should, to some extent, affect the ∑ClGaCl values. In this regard, buried volume calculations were performed (Table 1), which indeed revealed that **B** is notably less bulky than **2** and the other CPCs in Table 1. This might explain the relatively larger ∑ClGaCl of **B** compared to **2**. Additionally, as shown in Table S11, the Wiberg bond index of the C1─C2 and C1─P bonds of CPCs (R_2_C2═C1═PR_3_) aligns well with the π‐accepting ability of the carbene ligands. The C1─C2 bond order in **2** is higher than those in **A**–**E**, as the carbene unit in **2** has a higher π‐accepting ability. The C1─P bond strength generally follows an inverse trend relative to the C1─C2 bond. On the other hand, the natural population analysis (NPA) charge on C1 (Table S11) is not strongly correlated with electron‐donating ability.

The reaction of **2** with borane dimethylsulfide leads to the formation of adduct **6**. The single‐crystal structure shows a B2─C1 bond length of 1.622(4) Å (Figure 7, left), which is comparable to other C → borane dative bonds. Hydride abstraction of **6** by using B(C₆F₅)₃ leads to the formation of the carboranyl CPC stabilized B_2_H_5_^+^ species **7** (Figure S67). The presence of HB(C₆F₅)₃^−^ counter anion was indicated by the doublet (¹*J*(¹H,¹¹B) = 90.2 Hz) at −25.4 ppm. The hydride‐bridged structure of **7** was indicated by the characteristic broad ^1^H NMR signal at −3.88 ppm.^[^ ^89^, ^90^ ^]^

**Figure 7:**
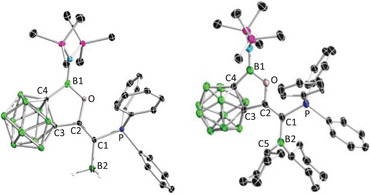
Single crystal structures of **6** (left) and **8** (right). Thermal ellipsoids are drawn at 50% probability level. The hydrogen atoms have been removed for clarity. Selected bond lengths (Å) and angles (°): For **6**: C1–C2 1.338(3), C2–C3 1.506(3), C3–C4 1.658(3), C1–P 1.794(3), B–C4 1.591(4), B–O 1.395(3), C1–B2 1.622(4), O–B–C4 107.1(2), P–C1–C2 115.16(19), C2–C1–B2 130.4(2), B2–C1–P 114.48(17).; For **8**: C1–C2 1.358(10), C2–C3 1.511(8), C3–C4 1.657(9), C1–P 1.803(6), B–C4 1.589(10), B–O 1.413(11), C1–B2 1.588(10), O–B–C4 106.6(6), P–C1–C2 114.0(5).

Moreover, **2** could react with Cy₂BCl (Cy = cyclohexyl) to yield the carboranyl CPC stabilized borenium compound **8**. The single crystal structure shows that the cation consists of a Cy₂B⁺ unit coordinated to the carbone center, while the counter anion is Cy₂BCl₂^−^. The Cy₂B⁺ unit and the central carbon of **2** are arranged orthogonally (C2─C1─B2─C5 72.88°) due to the steric congestion between the bulky substituents borenium and the carbone. As a consequence, the second electron pair of the carbone center could not effectively donate to the empty orbital on the borenium center. This can indeed be reflected by the relatively long B2─C1 distance (1.588(10) Å).

The calculations show that **2** has a higher PA than carbenes such as IMes (Table 1). Besides, in our recently published NHC‐catalyzed proton transfer reaction, the proposed mechanism was also based on the higher PA of carbones compared to carbenes.^[^ ^68^ ^]^ To our delight, the successful isolation of **2** allows us to verify this experimentally. Specifically, a proton competition experiment between **2** and IMes was performed. Compound **2** was treated with an equimolar amount of 1,3‐bis(2,4,6‐trimethylphenyl)‐4,5‐dihydroimidazolium tetrafluoroborate ([IMes‐H]^+^[BF_4_]^−^) at room temperature (Scheme 3). Monitoring the reaction by ^31^P NMR spectrum showed the complete consumption of **2** (δ^31^P − 0.4) within 10 min and the formation of a new species showing a downfield‐shifted resonance at δ^31^P 12.99. The ^1^H NMR spectrum showed a new doublet at 4.64 ppm with a coupling constant of ^2^ *J*_HP_ = 14.58 Hz, which turned to a singlet signal on the ^1^H{^31^P} NMR spectrum (see Figures S44 and S46.). This result is indicative of the protonation of **2**. However, no free carbene was observed. Instead, the new signals at −142.5 ppm in the ^19^F spectrum and at −0.8 ppm in the ^11^B NMR are consistent with those reported for the [IMes‐BF_3_] adduct.^[^ ^91^, ^92^ ^]^ This is not very surprising, as the Lewis basic IMes and the Lewis acidic 3‐coordinate boron of [**2**‐H]^+^ might form a frustrated Lewis pair (FLP), which split BF_4_^−^ into BF_3_ and F^−^ cooperatively. The former forms an adduct with IMes, while the latter binds to the Lewis acidic 3‐coordinate boron of [**2**‐H]^+^ to generate **9** that has been structurally characterized (Figure 8, left).

**Scheme 3:**
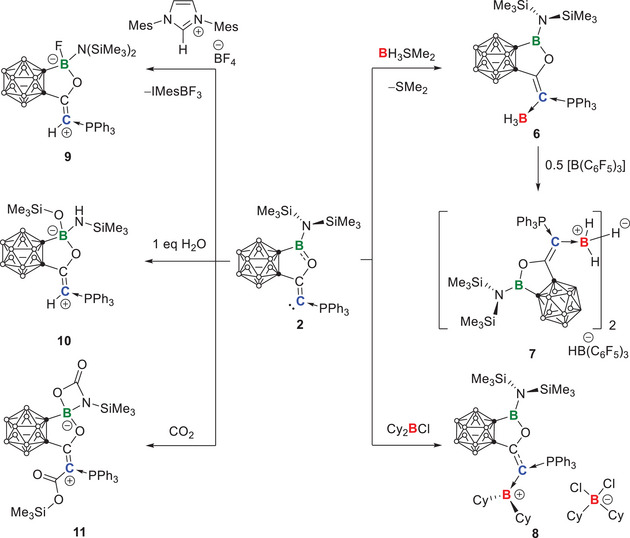
Reactivity of **2**.

**Figure 8:**
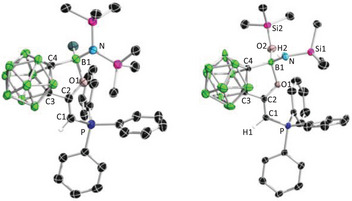
Single crystal structures of **9** (left) and **10** (right). Thermal ellipsoids are drawn at 50% probability level. The hydrogen atoms have been removed for clarity. Selected bond lengths (Å) and angles (°): For **9**: C1─C2 1.351(2), C2─C3 1.504(2), C3─C4 1.638(2), C1─P 1.7574(15), B─C4 1.681(2), B─O 1.560(2), O─B─C4 99.43(12), P─C1─C2 124.42(12); For **10**: C1─C2 1.349(4), C2─C3 1.513(4), C3─C4 1.623(4), C1─P 1.750(3), B─C4 1.673(4), B─O 1.648(4), B1─O2 1.423(4), O─B─C4 97.0(2), P─C1─C2 123.1(2).

Compound **2** can be easily protonated by water as well. A reaction between **2** and water was performed in a 1:1 stoichiometry using a saturated water solution in toluene. ^1^H NMR analysis confirmed the protonation at the carbone center, displaying again the characteristic doublet at 4.37 ppm (^2^ *J*_HP_ = 18.89 Hz). The ^13^C spectrum showed an upfield shift from 81.8 ppm (*J*_CP_ = 43.82 Hz) to 78.8 ppm (*J*_CP_ = 19.65 Hz), likely due to protonation. To further confirm this, single crystals (89% yield) suitable for X‐ray diffraction analysis were grown from a concentrated toluene solution at −30 °C. The single crystal structure clearly indicated the protonation of the carbone center by water (see H1 in Figure 8, right). However, this also leads to the formation of the basic hydroxide anion (HO^−^) that will be accommodated by the acidic boron (B1 in Figure 8, right) in the backbone. Subsequently, due to the strong oxophilicity of silicon and the spatial availability, the hydroxide moiety exchanges its proton for a trimethylsilyl group from the neighboring N(SiMe_3_)_2_ group, generating the final product **10** (Scheme 3).

Furthermore, the nucleophilicity of **2** allows it to react with CO_2_. A solution of **2** in C_6_D_6_ was bubbled with CO₂ for 1 min. Then, the reaction progress was monitored by ^31^P‐ and ^11^B‐NMR spectroscopy. After 90 min, the signal of the **2** (δ^31^P − 0.4, δ^11^B 34.6) completely disappeared and the new signals appeared at δ^31^P 19.4 and δ^11^B 5.0. After removing all volatiles under reduced pressure, the resulting pale‐yellow solid was washed with hexane and recrystallized from toluene, yielding product **11** as a colorless crystalline solid in 42% yield. Colorless crystals suitable for X‐ray diffraction were obtained by slow diffusion of hexane into a saturated toluene solution at −30 °C. The single crystal structure (Figure 9) revealed that **2** reacted with two equivalents of CO_2_. In the first step, one equivalent of CO₂ should be added to the carbone center, forming a carboxylate intermediate (**Int1**, Scheme 4). Due to the strong oxophilicity of silicon, the activated CO_2_ grabs one of the trimethylsilyl groups to form **Int2**. Next, the nucleophilic nitrogen and the adjacent electrophilic boron of **Int2** grabs the second CO_2_ cooperatively to yield the final product **11**.

**Figure 9:**
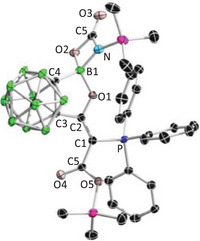
Single crystal structures of **11**. Thermal ellipsoids are drawn at 50% probability level. The hydrogen atoms have been removed for clarity. Selected bond lengths (Å) and angles (°): C1─C2 1.351(2), C2─C3 1.504(2), C3─C4 1.638(2), C1─P 1.7574(15), B─C4 1.681(2), B─O 1.560(2), O─B─C4 99.43(12), P─C1─C2 124.42(12).

**Scheme 4:**
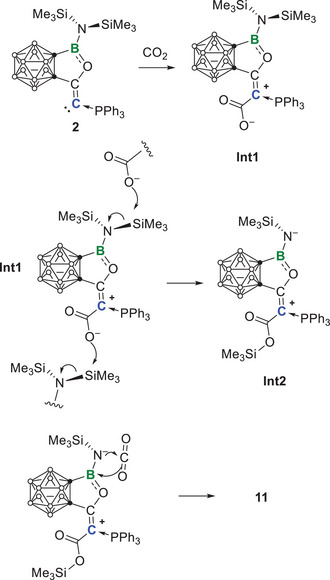
Plausible reaction mechanism for the formation of **11**.

In conclusion, the *ortho*‐carboranyl carbophosphinocarbene was successfully synthesized through click between the super‐strained carborane‐fused borirane and the Bestmann ylide. The remarkable electron‐donating ability of the carboranyl CPC was demonstrated both experimentally (i.e., TEP, ∑ClGaCl) and theoretically (i.e., frontier orbital analyses, PA). Reactivity studies revealed the versatility of carboranyl CPC as a stabilizing ligand, a strong nucleophile as well as a strong Brønsted base, enabling the stabilization of borenium cations, the activation of CO₂, and the deprotonation of imidazolium, respectively. Further exploration of super‐strained C_2_B‐boracycles as click reagents for the synthesis of boron‐containing functional molecules and ligands, as well as systematic studies on carboranyl CPC, are currently underway in our laboratory.

## Conflict of Interests

The authors declare no conflict of interest.

## Supporting information



Supporting Information

Supporting Information

## Data Availability

The data that support the findings of this study are available in the supplementary material of this article.
