# Supplementary material for: Conversion of Bestmann Ylide into Carbophosphinocarbene
Source: Angew Chem Int Ed Engl. 2025 Apr 7;64(22):e202501955. doi: 10.1002/anie.202501955 (PMC12105685; doi:10.1002/anie.202501955)
Supplement: Supplementary file 1 — Supporting Information [file ANIE-64-e202501955-s002.pdf]

Supporting Information

Conversion of Bestmann Ylide into Carbophosphinocarbene

Libo Xiang, Junyi Wang, Niclas Knoblauch, Alexander Matler, Qing Ye*

*Corresponding author, E-mail: qing.ye@uni-wuerzburg.de

Table of Contents

Experimental procedures ***3***

Synthesis and Spectral Data ***4***

TEP ***43***

Calculation details ***57***

References ***59***

Experimental procedures

General remarks

Unless otherwise noted, the following conditions apply.

All the manipulations were carried out using standard schlenk lines or glovebox under an argon atmosphere. All the solvents were dried following standard techniques. C_6D_6 was distilled from Na/K and stored under an argon atmosphere before use. $CDCl_3$ was dried and stored over 4 molecular sieves before use. CD_3CN and THF-d_8 were distilled from CaH_2 under an inert atmosphere and stored with molecular sieves before use. Ph_3PCCO, $GaCl_3$, $BH_3(SMe_2)$, $PhBCl_2$, Cy_2BCl, $B(C_6F_5)_3$, bis(1,5-cyclooctadien)diiridium(I)dichlorid were purchased from commercial sources and used without further purification. The *o*-carborane-fused aminoborirane **1** was synthesized according to the literature.[1] Other reagents were used as received without further purification.

The nuclear magnetic resonance spectroscopy was recorded on a Bruker Avance-400 (^{1}H 400.1 MHz; ^{11}B 128.5 MHz; ^{13}C 101 MHz; ^{31}P: 162. MHz; ^{19}F 376 MHz) spectrometer at room temperature. ^{11}B NMR, $^{11}B\{^{1}H\}$ spectra are referenced relative to 15% $BF_3{\cdot}OEt_2$. $^{31}P\{^{1}H\}$ NMR chemical shifts are relative to 85% H_3PO_4. ^{19}F NMR chemical shifts are referenced relative to trifluoromethane. High-resolution mass spectrometry (HRMS) was performed with a Thermo Fisher Scientific Q-Exactive MS System. Elemental analysis (C, H, N) was performed on a vario micro cube CHNS analyzer.

Synthesis and Spectral Data

Synthesis of 2

To a solution of borirane **1** (100 mg, 0.32 mmol) in 4 mL of toluene at room temperature, Ph_3PCCO (97 mg, 0.32 mmol) was added rapidly under stirring. The reaction mixture was stirred thoroughly and then cooled to –35 °C, where it was maintained for 12 hours. The resulting pale-yellow powder was isolated as the analytically pure product. Light-yellow crystals suitable for X-ray diffraction were obtained by storing a saturated diethyl ether solution of the product at –30 °C for 24 hours. Yield: 179 mg (91%)

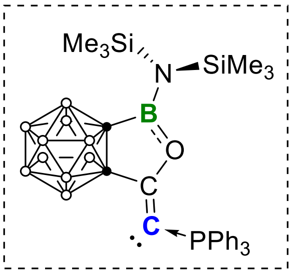

^{1}H NMR (C_6D_6): δ = 7.63 to 7.58 (m, 6H, H of Ph), 7.02 to 6.93 (m, 9H, H of Ph), 3.70 to 2.08 (m, 10H, B*H*), 0.06 (s, 18H, Si*Me₃*); **^{1}H{^{11}B} NMR** (C_6D_6): δ = 7.63 to 7.58 (m, 6H, H of Ph), 7.02 to 6.94 (m, 9H, H of Ph), 3.48 (s, 2H, B*H*), 3.21 (s, 1H, B*H*), 3.07 (s, 2H, B*H*), 2.97(s, 1H, B*H*), 2.08(s, 4H, B*H*), 0.06 (s, 18H, Si*Me₃*); **^{11}B NMR** (C_6D_6): δ = 34.6 (s, Cb*B*O), –1.8 (d, *J* = 126.3 Hz, B_{Cb}), –6.4 (d, *J* = 131.9 Hz, B_{Cb}), –9.0 to –11.1 (m, B_{Cb}); **^{11}B{^{1}H} NMR** (C_6D_6): δ = 35.2 (Cb*B*O), –1.8 (B_{Cb}), –6.4 (B_{Cb}), –9.7 (B_{Cb}), –11.1 (B_{Cb}); **^{13}C NMR** (C_6D_6): δ = 141.6 (d, J_{PC} = 6.32 Hz, Cb*C*O), 133.4 (d, J_{PC} = 9.55 Hz, C_{Ph}), 131.7 (d, J_{PC} = 2.73 Hz, C_{Ph}), 129.9 (d, J_{PC} = 86.69 Hz, C_{Ph}), 128.8 (d, J_{PC} = 11.71 Hz, C_{Ph}), 81.8 (d, J_{PC} = 43.82 Hz, *C*(0)), 3.5 (Si*Me₃*); **^{31}P{^{1}H} NMR** (C_6D_6): –0.44; **HRMS (m/z)**: $[M+H]^+$ calcd. for $C_{28}H_{44}ONB_{11}PSi_2$, 618.3717; found 618.3741.

Synthesis of 3

Cyclooctadiene iridium(I) chloride dimer (50.0 mg, 74.4 µmol, 1.0 eq) was dissolved in 2 mL of toluene. To this solution, a suspension of compound **2** (110 mg, 179 µmol, 2.4 eq) in 2 mL of toluene was added dropwise at room temperature with stirring. The reaction mixture was stirred continuously for 24 hours, resulting in the precipitation of a solid. The precipitate was collected by filtration, washed with hexane (3 × 2 mL), and recrystallized using a minimal amount of tetrahydrofuran (THF). The product was isolated as a yellow powder. Yellow crystals suitable for X-ray diffraction were obtained by allowing hexane to diffuse into a saturated THF solution of the product. Yield: m = 120 mg (85 %).

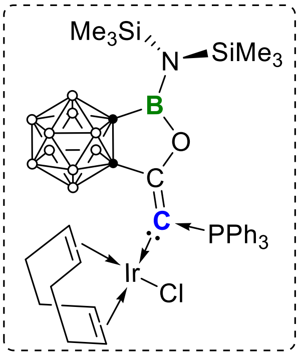

^{1}H NMR ($CDCl_3$): *δ* = 7.85 to 7.29 (m, 15H, H of Ph), 4.49 to 4.44 (m, 1H, H_{COD}), 3.89 to 3.85 (m, 1H, H H_{COD}) , 3.33 to 3.30 (m, 1H, H H_{COD}), 3.03 to 1.74 (m, 13H, m, B*H* + H_{COD}), 1.40 to 1.35 (m, 1H, H H_{COD}), 1.22 to 1.14 (m, 2H, H H_{COD}), 1.09 to 1.07 (m, 1H, H H_{COD}), 0.96 to 0.91 (m, 2H, H H_{COD}), –0.20 (s, 18H, Si*Me₃*); **^{1}H{^{11}B} NMR** ($CDCl_3$): *δ* = 7.68 to 7.26 (m, 15H, H of Ph), 4.49 to 4.45 (m, 1H, H_{COD}), 4.08

(s, 1H, B*H*), 3.90 to 3.85 (m, 1H, H H_{COD}) , 3.34 to 3.29 (m, 1H, H H_{COD}), 3.20 (s, 1H, B*H*), 2.66 (s, 1H, B*H*), 2.47 (s, 1H, B*H*), 2.39 to 2.14 (m, 8H, m, B*H* + H_{COD}), 1.41 to 1.34 (m, 1H, H H_{COD}), 1.21 to 1.14 (m, 1H, H H_{COD}), 1.09 to 1.05 (m, 2H, H H_{COD}), 0.97 to 0.92 (m, 2H, H H_{COD}), –0.20 (s, 18H, Si*Me₃*); **^{11}B NMR** ($CDCl_3$): δ = 33.2 (s, Cb*B*), –2.0 (s, B_{Cb}), –5.0 (d, J = 211.9 Hz, B_{Cb}), –8.4 (d, J = 288.3 Hz, B_{Cb}), –10.8 (d, J = 362.3 Hz, B_{Cb}); **^{11}B{^{1}H} NMR** ($CDCl_3$): δ = 34.2 (s, Cb*B*), –1.6 to –2.4 (m, B_{Cb}), –4.0 to –11.4 (m, B_{Cb}); **^{13}C NMR** ($CDCl_3$): δ = 148.2 (d, J_{CP} = 6.17 Hz, Cb*C*O), 135.4 to 127.6 (m, C_{Ph}), 116.7 (d, J_{CP} = 7.83 Hz, Ir*C*(0)P), 79.5 (d, J_{CP} = 28.37 Hz, $C(sp^2)_{COD}$), 78.0 (d, J_{CP} = 1.93 Hz, $C(sp^2)_{COD}$), 77.6 (d, J_{CP} = 3.44 Hz, $C(sp^2)_{COD}$), 77.2 (m (below chloroform peak), $C(sp^2)_{COD}$), 52.7 (s, C_{Cb}), 51.7 (s, C_{Cb}), 32.4 (s, $C(sp^3)_{COD}$), 32.3 (s, $C(sp^3)_{COD}$), 29.8 (s, $C(sp^3)_{COD}$), 28.3 (s, $C(sp^3)_{COD}$), 2.9 (Si*Me₃*); **^{31}P{^{1}H} NMR** ($CDCl_3$): δ = 18.4 (s); **HRMS (m/z)**: $[M]^+$ calc. for $C_{36}H_{55}B_{11}ClIrNOPSi_2$, 951.3974; found 951.3971.

Synthesis of 4

A solution of compound **3** (100.0 mg, 105 µmol) in dichloromethane (15 mL) was prepared and degassed. The solution was stirred under a carbon monoxide atmosphere for 10 minutes, during which the color changed sequentially from yellow to orange to deep red and finally reverted to a lighter yellow than the initial solution. Afterward, all volatiles were removed under reduced pressure, and the resulting solid was washed with hexane (2 × 3 mL). Recrystallization from toluene yielded the product as a yellow powder. Yellow crystals suitable for X-ray diffraction were obtained by the slow evaporation of a saturated toluene solution at room temperature. Yield: m = 84.3 mg (89 %)

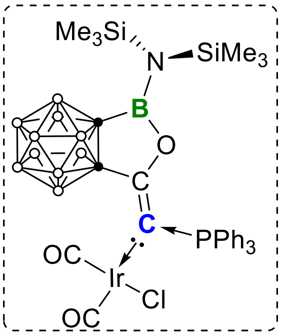

^{1}H NMR (C_6D_6): δ = 7.74 (dd, J_{HP} = 12.10 Hz, J_{HH} = 7.55 Hz, 6H, H of Ph), 7.04 to 6.94 (m, 9H, H of Ph), 3.8 to 1.8 (m, 10H, B*H*), –0.21 (s, 18H, Si*Me₃*); **^{1}H{^{11}B} NMR** (C_6D_6): δ = 7.72 (dd, J_{HP} = 12.34 Hz, J_{HH} = 7.59 Hz, 6H, H of Ph), 7.04 to 6.94 (m, 9H, H of Ph), 4.62 (s, 1H, B*H*), 3.98 (s, 1H, B*H*), 3.18 (s, 1H, B*H*), 3.02 (s, 2H, B*H*), 2.99 (m, 1H, B*H*), 2.82 (s, 1H, B*H*), 2.64 (s, 1H, H_{Cb}), 2.57 (s, 2H, B*H*), –0.20 (s, 18H,Si*Me₃*); **^{11}B NMR** (C_6D_6): δ = 34.5 (s, Cb*B*), 1.2 to –9.8 (m, B_{Cb}); **^{11}B{^{1}H} NMR** (C_6D_6): δ = 34.4 (s, Cb*B*), –0.8 (s, B_{Cb}), –3.2 (s, B_{Cb}), –5.4 to –6.4 (m, B_{Cb}), –8.5 to –11.6 (m, B_{Cb}); **^{13}C NMR** (C_6D_6): δ = 180.0 (d, J_{CP} = 1.17 Hz, *C*O), 170.6 (s, *C*O), 154.1 (d, J_{CP} = 6.03 Hz, Cb*C*O), 134.4 (d, J_{CP} = 9.84 Hz, C_{Ph}), 132.9 (d, J_{CP} = 2.98 Hz, C_{Ph}), 128.8 (d, J_{CP} = 12.47 Hz, C_{Ph}), 127.8 (d, J_{CP} = 23.19 Hz, C_{Ph}), 110.2 (d, J_{CP} = 25.48 Hz, C(0)), 79.0 (s, C_{Cb}), 78.7 (s, C_{Cb}), 2.8 (Si*Me₃*); **^{31}P{^{1}H} NMR** (C_6D_6): δ = 22.3 (s); **IR (PhMe)**: ṽ (CO) = 2050, 1967 cm^{-1}; **HRMS (m/z)**: $[M]^+$ calc. for $C_{30}H_{43}B_{11}ClIrNO_3PSi_2$, 899.2933; found 899.2928.

Synthesis of 5

To a solution of compound **2** (93 mg, 0.15 mmol) in 2 mL of toluene at room temperature, A toluene solution of $GaCl_3$ (26 mg, 0.15 mmol) was added dropwise while stirring. was added dropwise. The reaction mixture was stirred for 30 minutes, leading to the formation of a precipitate. The precipitate was collected by filtration, washed with hexane (2 × 2 mL), and dried under vacuum. The analytically pure product was obtained as a white powder. Crystals suitable for X-ray diffraction were obtained by storing a saturated DCM solution of the product at –30 °C for 24 hours. Yield: m = 101 mg (85 %).

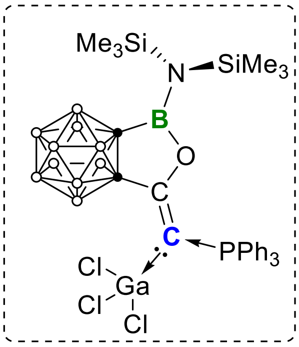

^{1}H NMR ($CDCl_3$): δ = 7.84 to 7.78 (m, 6H, H of Ph), 7.72 to 7.68 (m, 3H, H of Ph), 7.62 to 7.57 (m, 6H, H of Ph), 2.90 to 1.96 (m, 10H, B*H*), –0.08 (s, 18H, Si*Me$_3$*); **^{1}H{^{11}B} NMR** ($CDCl_3$): δ = 7.74 to 7.68 (m, 6H, H of Ph), 7.62 to 7.58 (m, 3H, H of Ph), 7.52 to 7.47 (m, 6H, H of Ph), 3.28 (s, 2H, B*H*), 2.43 (s, 2H, B*H*), 2.37 (s, 2H, B*H*), 2.12 (s, 2H, B*H*), 2.08 (s, 2H, B*H*), –0.17 (s, 18H, Si*Me$_3$*); **^{11}B NMR** ($CDCl_3$): δ = 34.3 (s, Cb*B*O), –2.1 (br, B_{Cb}), –6.4 (d, *J* = 123.9 Hz, B_{Cb}), –10.7 (br, B_{Cb}); **^{11}B{^{1}H} NMR** ($CDCl_3$): δ = 33.8 (Cb*B*O), –1.1 (B_{Cb}), –3.0 (B_{Cb}), –5.4 (B_{Cb}), –9.9 (B_{Cb}), –12.2 (B_{Cb}); **^{13}C NMR** ($CDCl_3$): δ = 137.9 (s, Cb*C*O), 134.4 (d, J_{PC} = 9.85 Hz, C_{Ph}), 133.9 (d, J_{PC} = 3.23 Hz, C_{Ph}), 129.6 (d, J_{PC} = 12.69 Hz, C_{Ph}), 122.2 (d, J_{PC} = 90.71 Hz, C_{Ph}), 77.2 (s, *C*(0)), 3.2 (Si*Me$_3$*); **^{31}P{^{1}H} NMR** ($CDCl_3$): 20.17; **HRMS (m/z)**: $[M-Cl]^-$ calcd. For $C_{28}H_{43}B_{11}NOSi_2PCl_2Ga$, 754.2050; found 754.2339.

Synthesis of 6

To a solution of compound **2** (93 mg, 0.15 mmol) in 2 mL of diethyl ether at room temperature, $BH_3{\cdot}SMe_2$ (0.15 mmol, 2 M in THF) was added. The mixture was stirred thoroughly and then cooled to −35 °C, which was maintained for 12 hours. The resulting white crystals were collected by filtration, washed with cold diethyl ether (2 × 1 mL), and dried under vacuum. The analytically pure product was obtained as a white powder. Crystals suitable for X-ray diffraction were obtained by storing a saturated THF solution of the product at –30 °C for 48 hours. Yield: m = 79 mg (82 %).

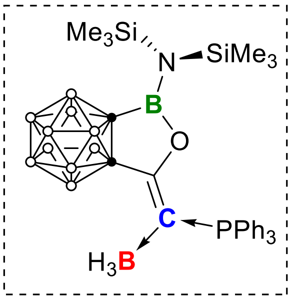

^{1}H NMR (THF-*d*8): δ = 7.67 to 7.62 (*m*, 9H, H of Ph), 7.56 to 7.51 (*m*, 6H, H of Ph), 3.70 to 2.08 (*m*, 10H, B*H*), 1.03 to 0.93 (*m*, 3H, B*H$_3$*), –0.12 (*s*, 18H, Si*Me$_3$*); **^{1}H{^{11}B} NMR** (THF-*d*8): δ = 7.67 to 7.62 (*m*, 6H, H of Ph), 7.55 to 6.94 (*m*, 9H, H of Ph), 3.20 (*s*, 2H, B*H*), 2.43 (*s*, 1H, B*H*), 2.36 (*s*, 2H, B*H*), 2.28 (*m*, 3H, B*H*), 2.19 (*s*, 2H, B*H*), 0.96 (*d*, 3H, B*H$_3$*), –0.12 (*s*, 18H, Si*Me$_3$*); **^{11}B NMR** (THF-*d*8): δ = 33.6 (*s*, Cb*B*O), –1.8 (*d*, *J* = 138.92 Hz, B_{Cb}), –4.1 (*d*, *J* = 130.82 Hz, B_{Cb}), –4.6 to –11.8 (*m*, B_{Cb}), –28.5 (*q*, *J* = 89.39 Hz, *B*H$_3$); **^{11}B{^{1}H} NMR** (THF-*d*8): δ = 33.2 (Cb*B*O), –1.8 (B_{Cb}), –4.1 (B_{Cb}), –7.1 (B_{Cb}), –9.5 (B_{Cb}), –12.4 (B_{Cb}), –28.5 (*B*H$_3$); **^{13}C NMR** (THF-*d*8): δ = 135.2 (*d*, J_{PC} = 9.36 Hz, C_{Ph}), 133.8 (*d*, J_{PC} = 2.83 Hz, C_{Ph}), 129.8 (*d*, J_{PC} = 12.25 Hz, C_{Ph}), 124.6 (*d*, J_{PC} = 87.23 Hz, C_{Ph}), 81.8 (*d*, J_{PC} = 43.82 Hz, *C*(0)), 3.3

(Si*Me*$_3$); **^{31}P{^{1}H} NMR** (THF-*d*8): 21.25; **HRMS (m/z)**: $[M+H]^+$ calcd. for $C_{28}H_{47}ONB_{12}PSi_2$, 632.4045; found 632.4064.

Synthesis of 7

To a 2 mL dichloromethane solution of $B(C_6F_5)_3$ (77 mg, 0.08 mmol) at room temperature, compound **6** (97 mg, 0.15 mmol) was added. The resulting colorless mixture was stirred for 15 minutes. The solvent was then removed under reduced pressure, and the residue was washed with hexane (2 × 2 mL). After drying under vacuum, compound **7** was obtained as a white solid. Yield: m = 142 mg (54 %).

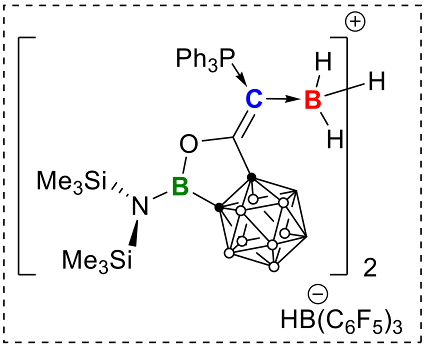

^{1}H NMR ($CDCl_3$): δ = 7.68 to 7.63 (m, 6H, H of Ph), 7.50 to 7.48 (m, 24H, H of Ph), 3.53 to 1.98 (m, 24H, B*H*), –0.10 (s, 36H, Si*Me*$_3$), –3.88 (br, 1H, Bridge *H*); **^{1}H{^{11}B} NMR** ($CDCl_3$): δ = 7.68 to 7.63 (m, 6H, H of Ph), 7.50 to 7.48 (m, 24H, H of Ph), 3.69 (s, 1H, B*H*), 3.01 (s, 4H, B*H*), 2.74 (s, 2H, B*H*), 2.51(s, 9H, B*H*), 2.30 (s, 5H, B*H*), 2.12 (s, 3H, B*H*), –0.10 (s, 36H, Si*Me*$_3$), –3.90 (br, 1H, Bridge *H*); **^{11}B NMR** ($CDCl_3$): δ = 30.0 (s, Cb*B*O), –2.0 (br, B_{Cb}), –6.8 (br, B_{Cb}), –13.7 (br, B_{Cb}), –25.4 (d, *J* = 90.2 Hz, H*B*(C_6F_5)$_3$); **^{11}B{^{1}H} NMR** ($CDCl_3$): δ = 29.8 (Cb*B*O), –2.2 (B_{Cb}), –6.3 (B_{Cb}), –14.1 (B_{Cb}), –25.6 (s, H*B*(C_6F_5)$_3$); **^{13}C NMR** ($CDCl_3$): δ = 156.5 (d, J_{PC} = 5.08 Hz, Cb*C*O), 149.5 to 147.0 (m, $C_{Ph\text{-}F}$), 134.0 (d, J_{PC} = 9.71 Hz, C_{Ph}), 129.4 (d, J_{PC} = 12.66 Hz, C_{Ph}), 128.6 (d, J_{PC} = 81.69 Hz, C_{Ph}), 120.6 (d, J_{PC} = 88.71 Hz, C_{Ph}), 77.2 (s, *C*cb), 3.0 (Si*Me*$_3$); **^{31}P{^{1}H} NMR** ($CDCl_3$): 22.86; **^{19}F{^{1}H} NMR** ($CDCl_3$): –133.38 (d, 2F, *J* = 20.12 Hz), –164.18 (t, 1F, *J* = 19.46 Hz), –167.20 (m, 2F); **HRMS (m/z)**: $[M]^+$ calcd. for $C_{56}H_{91}O_2N_2B_{24}P_2Si_4$, 1257.8011; found 1257.8006.

Synthesis of 8

To a solution of compound **2** (93 mg, 0.15 mmol) in 2 mL of diethyl ether at room temperature, Cy_2BCl (0.30 mmol, 1 M in Hex) was added dropwise. The mixture was stirred thoroughly and then cooled to −35 °C, which was maintained for 12 hours. The resulting white crystals were collected by filtration, washed with cold diethyl ether (2 × 3 mL), and dried under vacuum. The analytically pure product was obtained as a white powder. Crystals suitable for X-ray diffraction were obtained by storing a saturated ACN solution of the product at –30 °C for 24 hours. Yield: m = 106 mg (68 %).

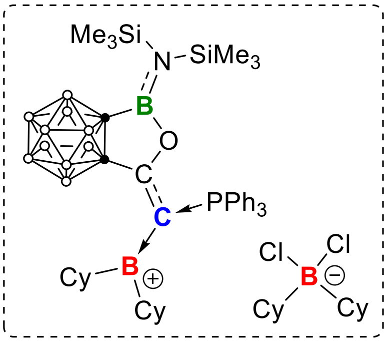

^{1}H NMR (CD_3CN): δ = 7.80 to 7.76 (m, 3H, H of Ph), 7.71 to 7.68 (m, 4H, H of Ph), 7.66 to 7.61 (m, 8H, H of Ph), 3.03 to 2.05 (m, 10H, B*H*), 1.71 to 1.63 (m, 20H, H of Cy), 1.49 to 1.45 (m, 1H, H of Cy), 1.25 to 1.10 (m, 12H, H of Cy), 1.07 to 0.99 (m, 8H, H of Cy), 0.89 to 0.79 (m, 1H, H of Cy), 0.53 to 0.47 (m, 2H, H of Cy), –0.12 (s, 18H, Si*Me$_3$*); **^{1}H{^{11}B} NMR** (CD_3CN): δ = 7.83 to 7.79 (m, 3H, H of Ph), 7.74 to 7.70 (m, 4H, H of Ph), 7.69 to 7.63 (m, 8H, H of Ph), 2.61 (s, 2H, B*H*), 2.46 (s, 2H, B*H*), 2.37 (s, 2H, B*H*), 2.26 (s, 2H, B*H*), 2.19 (s, 1H, B*H*), 2.15 (s, 1H, B*H*), 1.72 to 1.68 (m, 20H, H of Cy), 1.51 to 1.44 (m, 1H, H of Cy), 1.27 to 1.15 (m, 12H, H of Cy), 1.10 to 1.01 (m, 8H, H of Cy), 0.93 to 0.82 (m, 1H, H of Cy), 0.56 to 0.49 (m, 2H, H of Cy), –0.12 (s, 18H, Si*Me$_3$*); **^{11}B NMR** (CD_3CN): δ = 37.3 (s, Cb*B*O), 10.7 (*B*Cy), 7.1 (*B*Cy), –4.9 (d, *J* = 99.7 Hz, B_{Cb}), –7.5 (d, *J* = 149.2 Hz, B_{Cb}),), –11.1 (d, *J* = 142.3 Hz, B_{Cb}),; **^{11}B{^{1}H} NMR** (CD_3CN): δ = 37.2 (Cb*B*O), 10.7 (*B*Cy), 7.1 (*B*Cy), –5.1 (B_{Cb}), –7.6 (B_{Cb}), –11.1 (B_{Cb}); **^{13}C NMR** (CD_3CN): δ = 145.2 (s, Cb*C*O), 135.1 (d, J_{PC} = 3.30 Hz, C_{Ph}), 134.2 (d, J_{PC} = 11.05 Hz, C_{Ph}), 130.5 (d, J_{PC} = 13.16 Hz, C_{Ph}), 121.4 (d, J_{PC} = 92.64 Hz, C_{Ph}), 92.2 (s, *C*(0)), 30.1 (d, J_{PC} = 4.25 Hz, C_{Cy}), 29.2 (d, J_{PC} = 43.69 Hz, C_{Cy}), 28.6 (s, C_{Cy}), 27.9 (d, J_{PC} = 19.65 Hz, C_{Cy}), 30.1 (d, J_{PC} = 4.25 Hz, C_{Cy}),5.3 (Si*Me$_3$*); **^{31}P{^{1}H} NMR** (CD_3CN): 11.92.

Synthesis of 9

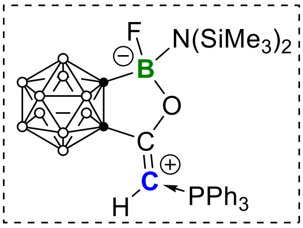

Compound **2** (93 mg, 0.15 mmol) was dissolved in 2 mL of dichloromethane at room temperature. A DCM solution of the [IMes-H]$^{+}$ (1,3-Bis(2,4,6-trimethylphenyl)-4,5-dihydroimidazolium tetrafluoroborate) (59 mg, 0.15 mmol) was added dropwise while stirring. The mixture was stirred thoroughly, then the solvent was removed under reduced pressure. The residue was extracted twice with toluene (1 mL each). The combined extracts were collected, and the solvent was removed under vacuum. The analytically pure product was obtained as a white powder. Crystals suitable for X-ray diffraction were obtained by storing a toluene solution of the product at –30 °C for 24 hours. Yield: m = 67 mg (70 %).

^{1}H NMR (C_6D_6): δ = 7.16 to 7.10 (m, 6H, H of Ph overlaped with C_6D_6), 7.00 to 6.96 (m, 3H, H of Ph), 6.92 to 6.88 (m, 6H, H of Ph), 4.64 (d, J_{HP} = 14.58 Hz, 1H, $H_{C(0)}$), 3.76 to 2.31 (m, 10H, B*H*), 0.26 s, 18H, Si*Me$_3$*); **^{1}H{^{11}B} NMR** (C_6D_6): δ = 7.16 to 7.10 (m, 6H, H of Ph), 7.01 to 6.97 (m, 3H, H of Ph), 6.93 to 6.88 (m, 6H, H of Ph), 4.64 (d, J_{HP} = 14.60 Hz, 1H, $H_{C(0)}$), 3.15 (s, 4H, B*H*), 3.08 (s, 1H, B*H*), 3.02 (s, 2H, B*H*), 2.94 (s, 1H, B*H*), 2.89 (s, 1H, B*H*), 2.82 (s, 1H, B*H*), 0.26 (s, 18H, Si*Me$_3$*); **^{1}H{^{31}P} NMR** (C_6D_6): δ = 7.16 to 7.12 (m, 6H, H of Ph), 7.01 to 6.97 (m, 3H H of Ph), 6.92 to 6.88 (m, 6H, H of Ph), 4.63 (s, 1H, $H_{C(0)}$), 3.39 to 2.29 (m, 10H, B*H*), 0.26 (s, 18H, Si*Me$_3$*); **^{11}B NMR** (C_6D_6): δ = 8.5 (s, Cb*B*), –4.1 (s, B_{Cb}). –6.8 (br, B_{Cb}), –10.5 (br, B_{Cb}); **^{11}B{^{1}H} NMR** (C_6D_6): δ = 8.4 (s, Cb*B*), –3.1 (s, B_{Cb}), –4.6 (s, B_{Cb}), –6.6 (s, B_{Cb}), –10.5 (s, B_{Cb}); **^{13}C NMR** (C_6D_6): δ = 175.0 (s, Cb*C*O), 133,7 (d, J_{CP} = 2.96 Hz, C_{Ph}), 133.2 (d, J_{CP} = 10.68 Hz, C_{Ph}), 129.4 (d, J_{CP} = 13.05 Hz, C_{Ph}), 121.6 (d, J_{CP} = 92.23 Hz, C_{Ph}), 79.7 (d, J_{CP} = 19.22 Hz, C(0)), 55.88 (s, C_{Cb}), 54.82 (s, C_{Cb}), 5.3 (d, J_{CF} = 1.65 Hz Si*Me$_3$*); **^{31}P{^{1}H} NMR** (C_6D_6): δ =

12.99 (s); **$^{19}F\{^1H\}$ NMR** (C_6D_6): –124.63 (s); **HRMS (m/z)**: $[M]^+$ calc. for $C_{28}H_{44}B_{11}NOSi_2PF$, 635.3779; found 635.3774.

Synthesis of 10

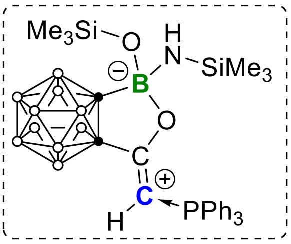

A saturated solution of water (10 µL) in toluene (15 mL) was prepared at room temperature and degassed as previously described. A portion of the solution (1.87 mL, 0.08 mmol, 1.0 eq) was added to compound **2** (50 mg, 0.08 mmol, 1.0 eq). The resulting mixture was stirred at room temperature for 2 hours. Since residual starting material was detected in the NMR spectra, an additional portion of the saturated water solution (1.87 mL, 81.2 µmol, 1.0 eq) was added, and stirring was continued for another 3 hours. Afterward, all volatiles were removed under vacuum. The analytical crystals suitable for X-ray diffraction were obtained by storing a toluene solution of the product at –30 °C for 24 hours. Yield: m = 46.8 mg (89 %).

^1H NMR (C_6D_6): δ = 7.22 to 7.17 (m, 5H, H of Ph), 7.03 to 6.99 (m, 3H, H of Ph), 6.97 to 6.93 (m, 7H, H of Ph), 4.37 (d, J_{HP} = 18.89 Hz, 1H, $H_{C(0)}$), 3.75 to 2.28 (m, 10H, B*H*), 0.71 (s, 1H, N*H*), 0.29 (s, 9H, O-Si*Me₃*), 0.12 (s, 9H, N-Si*Me₃*); **$^1H\{^{11}B\}$ NMR** (C_6D_6): δ = 7.20 to 7.15 (m, 5H, H of Ph), 7.04 to 6.99 (m, 3H, H of Ph), 6.91 to 6.96 (m, 7H, H of Ph), 4.37 (d, J_{HP} = 18.87 Hz, 1H, $H_{C(0)}$), 3.13 (s, 3H, B*H*), 3.03 (s, 1H, B*H*), 2.91 (s, 4H, B*H*), 2.82 (s, 2H, B*H*), 0.70 (s, 1H, N*H*), 0.28 (s, 9H, O-Si*Me₃*), 0.12 (s, 9H, N-Si*Me₃*); **$^1H\{^{31}P\}$ NMR** (C_6D_6): δ = 7.20 to 7.17 (m, 6H, H of Ph), 7.03 to 6.99 (m, 3H H of Ph), 6.97 to 6.93 (m, 6H, H of Ph), 4.38 (s, 1H, $H_{C(0)}$), 3.37 to 2.64 (m, 10H, B*H*), 0.71 (s, 1H, N*H*), 0.29 (s, 9H, O-Si*Me₃*), 0.12 (s, 9H, N-Si*Me₃*); **^{11}B NMR** (C_6D_6): δ = 10.1 (s, Cb*B*), –2.8 (s, B_{Cb}). –4.5 (s, B_{Cb}), –5.9 s, (s, B_{Cb}), –7.1 (s, B_{Cb}), –10.7 (s, B_{Cb}); **$^{11}B\{^1H\}$ NMR** (C_6D_6): δ = 10.3 (s, Cb*B*), –3.4 (s, B_{Cb}), –5.2 (s, B_{Cb}), –6.6 (s, B_{Cb}), –11.1 (s, B_{Cb}); **^{13}C NMR** (C_6D_6): δ = 173.9 (d, J_{CP} = 1.89 Hz, Cb*C*O), 131,1 (d, J_{CP} = 3.13 Hz, C_{Ph}), 130.9 (d, J_{CP} = 10.72 Hz, C_{Ph}), 127.2 (d, J_{CP} = 12.82 Hz, C_{Ph}), 120.9 (d, J_{CP} = 92.46 Hz, C_{Ph}), 78.8 (d, J_{CP} = 19.65 Hz, C(0)), 55.9 (s, C_{Cb}), 54.89 (s, C_{Cb}), 0.9 (s, O-Si*Me₃*), 0.2 (s, N-Si*Me₃*); **$^{31}P\{^1H\}$ NMR** (C_6D_6): δ = 14.90 (s); **HRMS (m/z)**: $[M]^+$ calc. for $C_{28}H_{45}B_{11}NO_2Si_2P$, 633.3823; found 633.3835.

Synthesis of 11

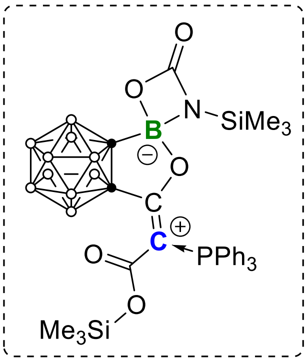

A solution of compound **2** (93 mg, 0.15 mmol) in toluene (5 mL) was prepared and degassed. The solution was placed under a carbon dioxide atmosphere and stirred for 90 minutes. After removing all volatiles under reduced pressure, the resulting pale-yellow solid was washed with hexane (3 × 2 mL). The crude product was recrystallized from toluene. Colorless crystals suitable for X-ray diffraction were obtained by allowing hexane to diffuse into a saturated toluene solution at −30 °C. Yield: m = 44 mg (42 %).

^{1}H NMR (C_6D_6): δ = 7.32 to 7.27 (m, 6H, H of Ph), 7.01 to 6.98 (m, 3H, H of Ph), 6.96 to 6.93 (m, 6H, H of Ph), 3.32 to 2.54 (m, 10H, B*H*), 0.15 (s, 9H, O-Si*Me*$_3$), –0.06 (s, 9H, N-Si*Me*$_3$); **^{1}H{^{11}B} NMR** (C_6D_6): δ = 7.33 to 7.28 (m, 6H, H of Ph), 7.02 to 6.99 (m, 3H, H of Ph), 6.97 to 6.93 (m, 6H, H of Ph), 3.21 (s, 1H, B*H*), 3.14 (s, 2H, B*H*), 3.05 (s, 3H, B*H*), 2.92 (s, 2H, B*H*), 2.85 (s, 1H, B*H*), 2.72 (s, 1H, B*H*), 0.15 (s, 9H, O-Si*Me*$_3$), –0.05 (s, 9H, N-Si*Me*$_3$); **^{11}B NMR** (C_6D_6): δ = 5.05 (s, Cb*B*N), –1.1 (d, *J* = 153.03 Hz, B_{Cb}), –5.1 (d, *J* = 158.13 Hz, B_{Cb}), –5.9 (B_{Cb}), –10.4 (s, B_{Cb}); **^{11}B{^{1}H} NMR** (C_6D_6): δ = 5.03 (s, Cb*B*N), –0.9 (B_{Cb}), –5.2 (B_{Cb}), –6.3 (B_{Cb}), –11.1 (B_{Cb}); **^{13}C NMR** (C_6D_6): δ = 170.9 (s, Cb*C*O), 133.6 (d, J_{PC} = 3.02 Hz, C_{Ph}), 133.2 (d, J_{PC} = 10.54 Hz, C_{Ph}), 129.2 (d, J_{PC} = 13.06 Hz, C_{Ph}), 119.6 (d, J_{PC} = 92.38 Hz, C_{Ph}), 87.3 (d, J_{PC} = 11.05 Hz, *C*(0)), 0.2 (s, O-Si*Me*$_3$), –1.3 (s, N-Si*Me*$_3$); **^{31}P{^{1}H} NMR** (C_6D_6): 19.43.

NMR Spectroscopy

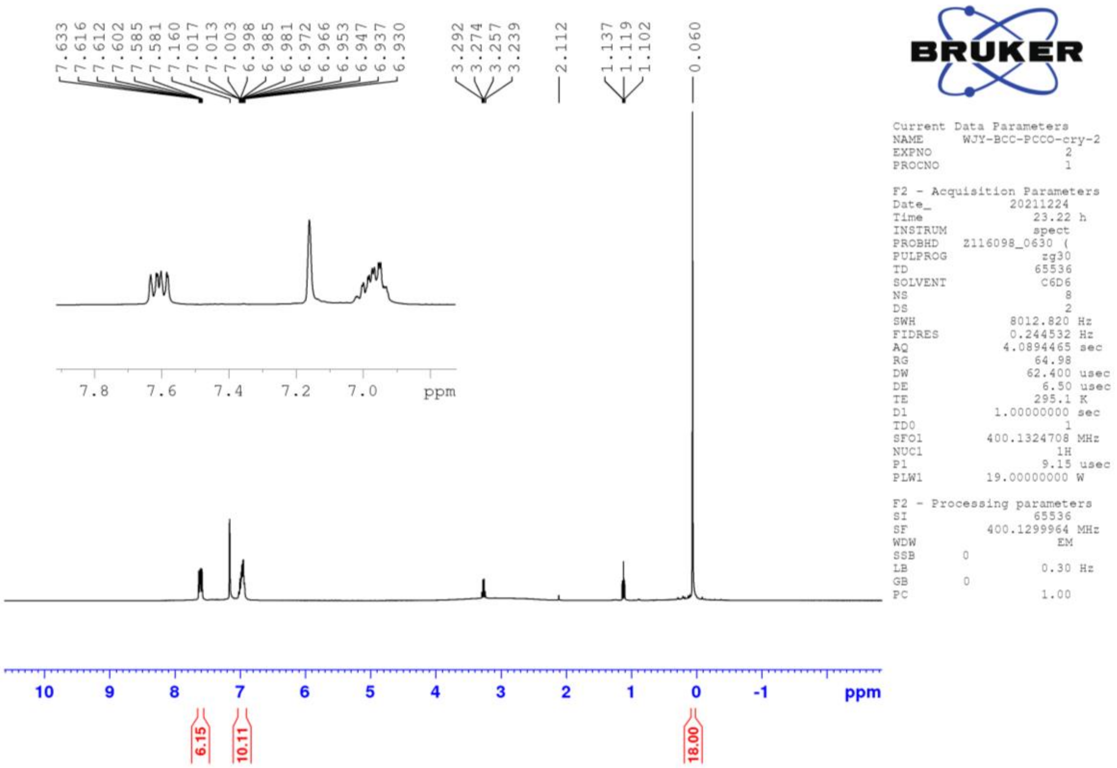

Figure S1. ^{1}H NMR spectra of **2** in C_6D_6 at 298 K.

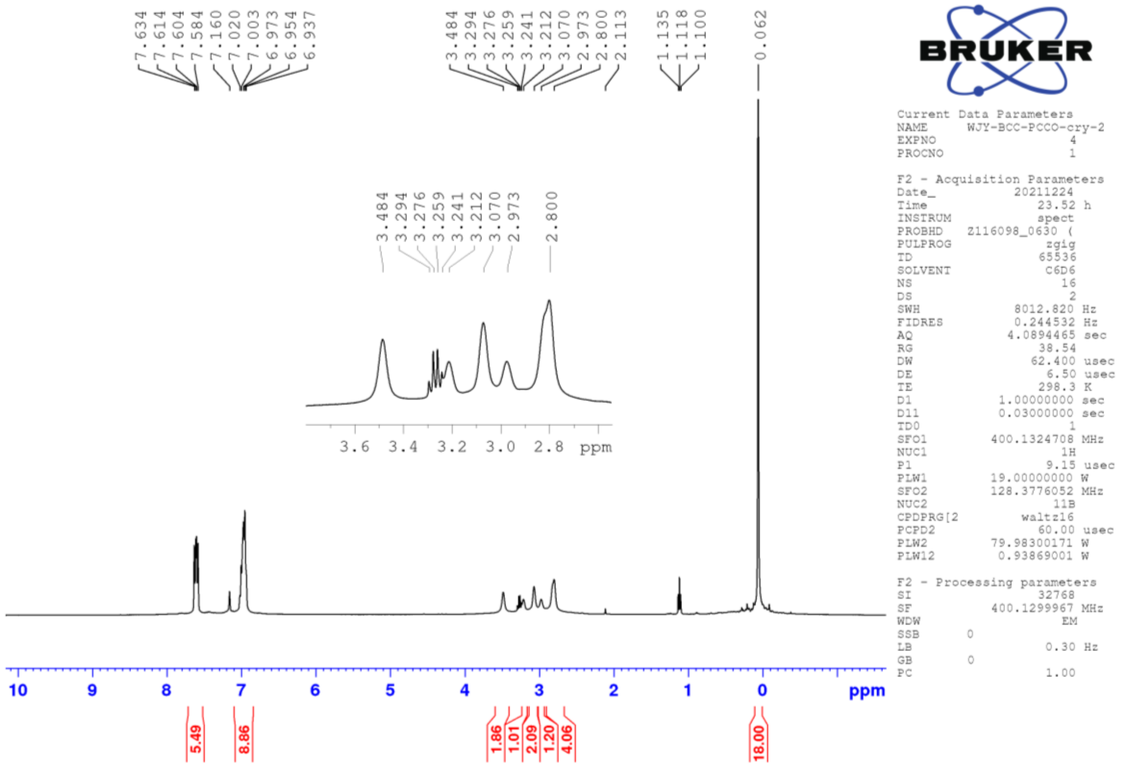

Figure S2. ^{1}H{^{11}B} NMR spectra of **2** in C_6D_6 at 298 K.

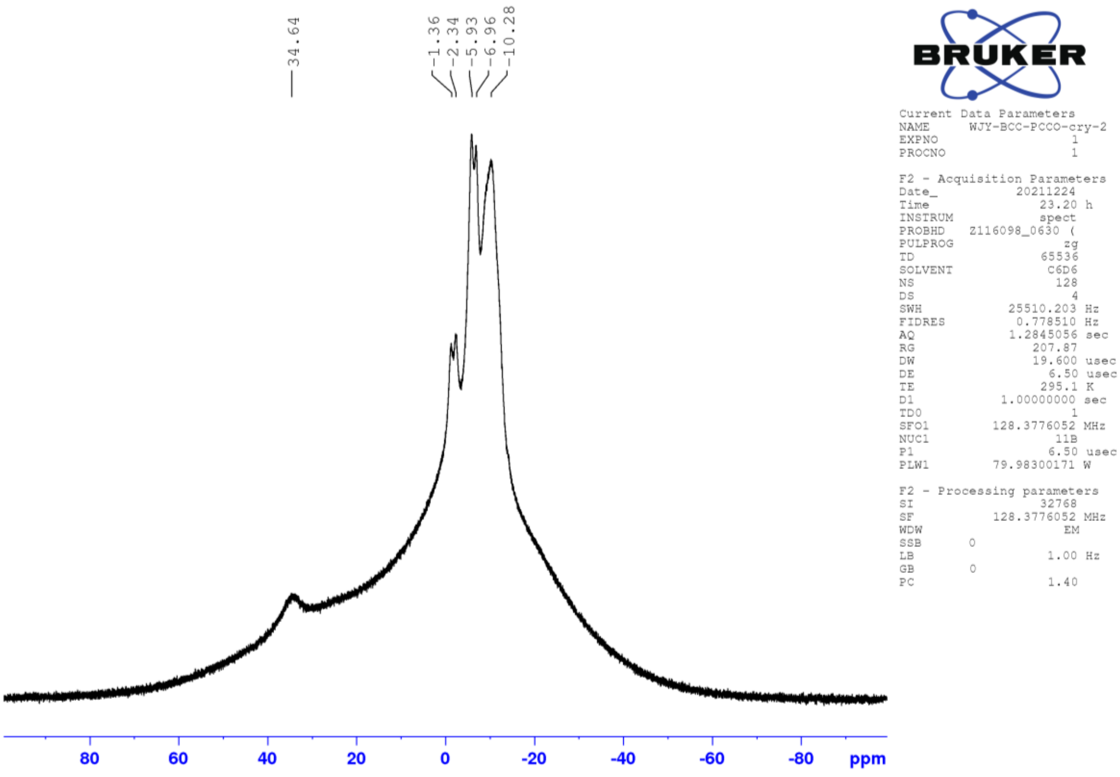

Figure S3. ^{11}B NMR spectra of **2** in C_6D_6 at 298 K.

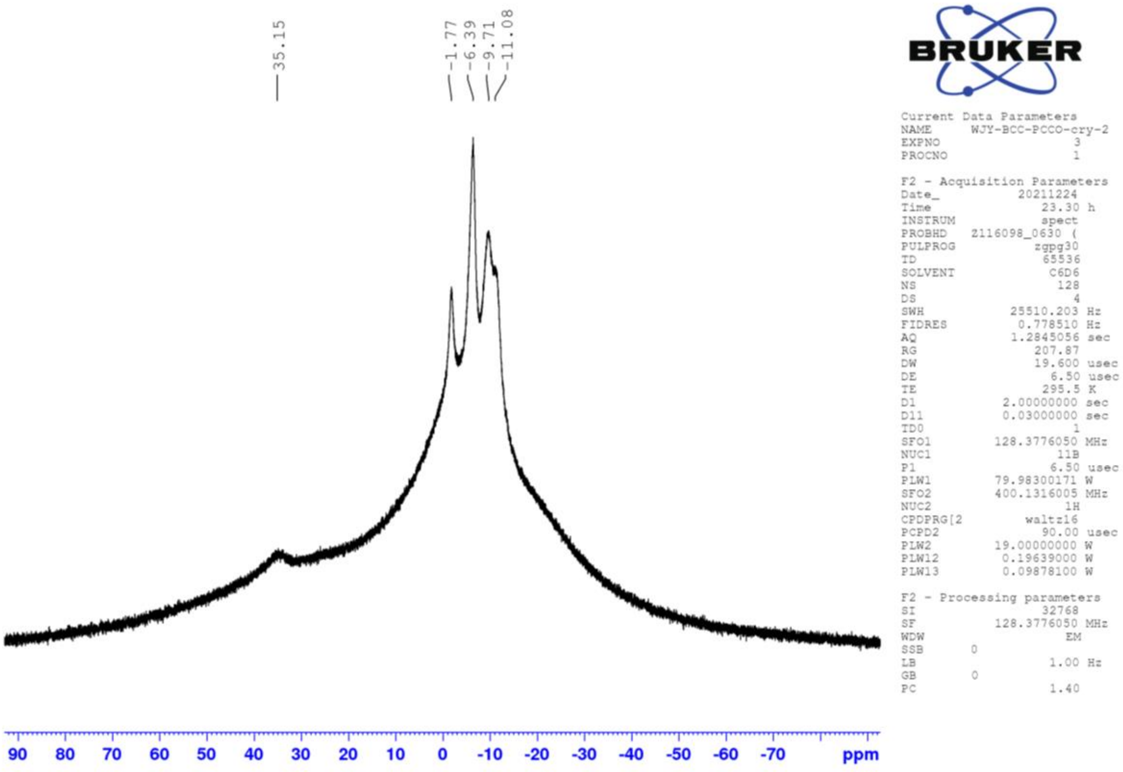

Figure S4. $^{11}B\{^1H\}$ NMR spectra of **2** in C_6D_6 at 298 K.

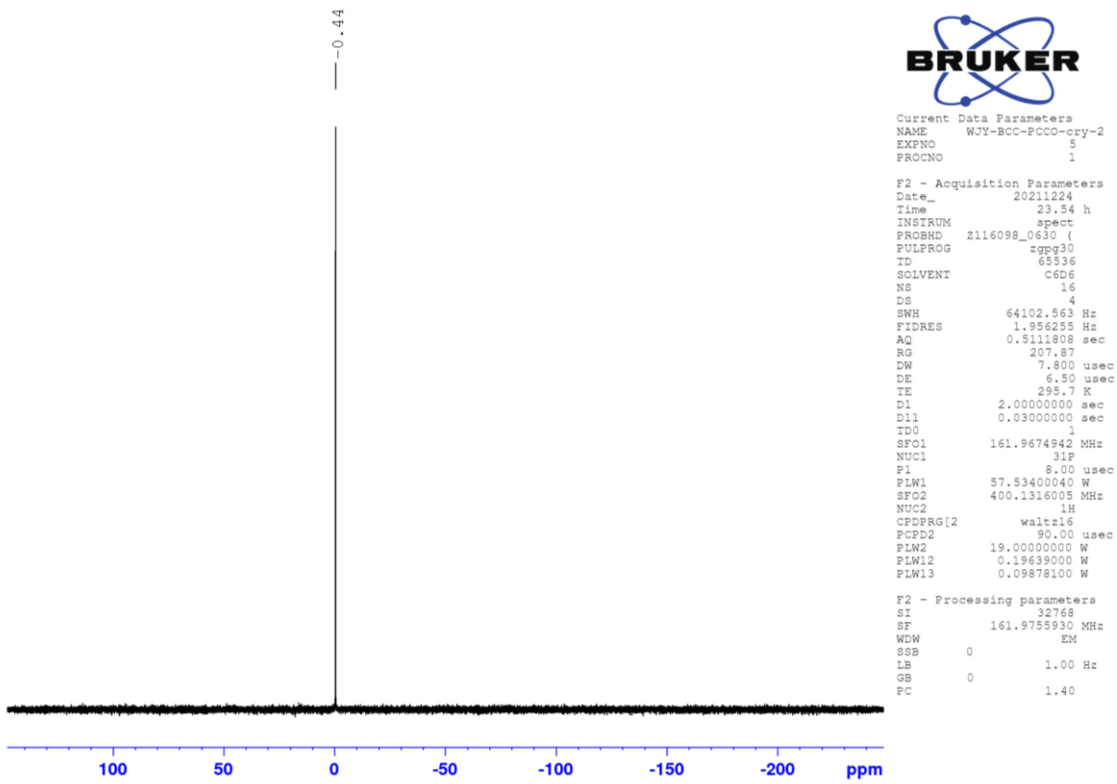

Figure S5. ^{31}P NMR spectra of **2** in C_6D_6 at 298 K.

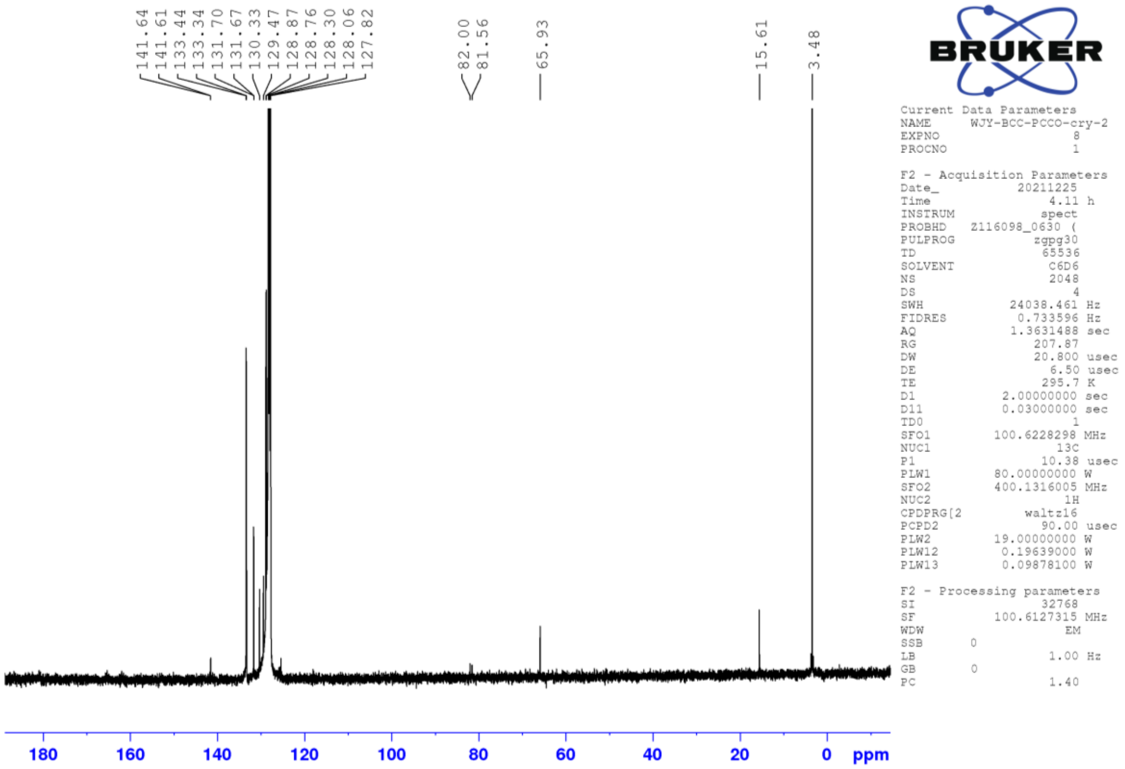

Figure S6. $^{13}C\{^1H\}$ NMR spectra of **2** in C_6D_6 at 298 K.

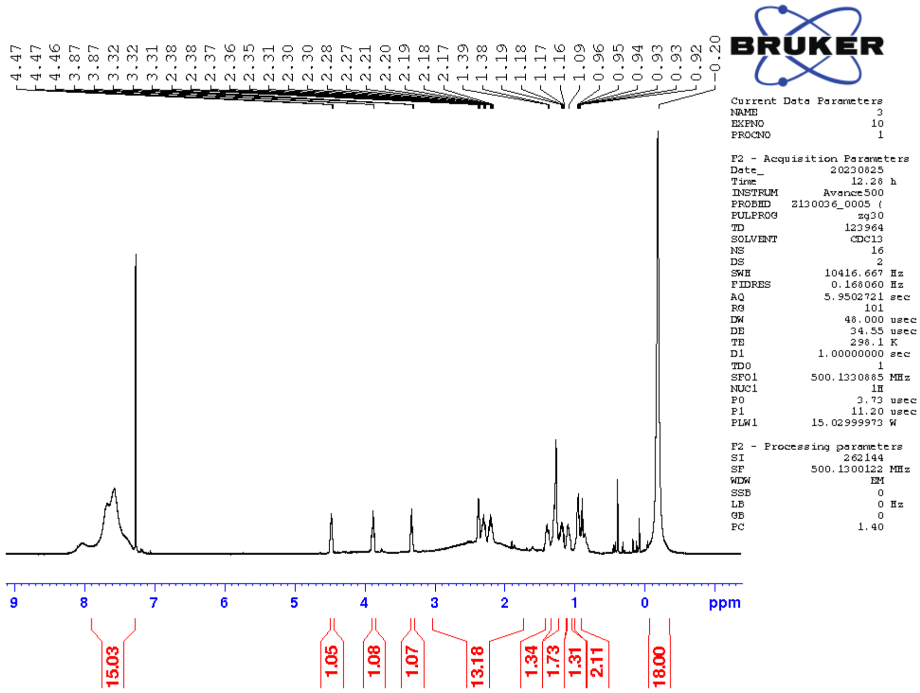

Figure S7. ^{1}H NMR spectra of **3** in $CDCl_3$ at 298 K.

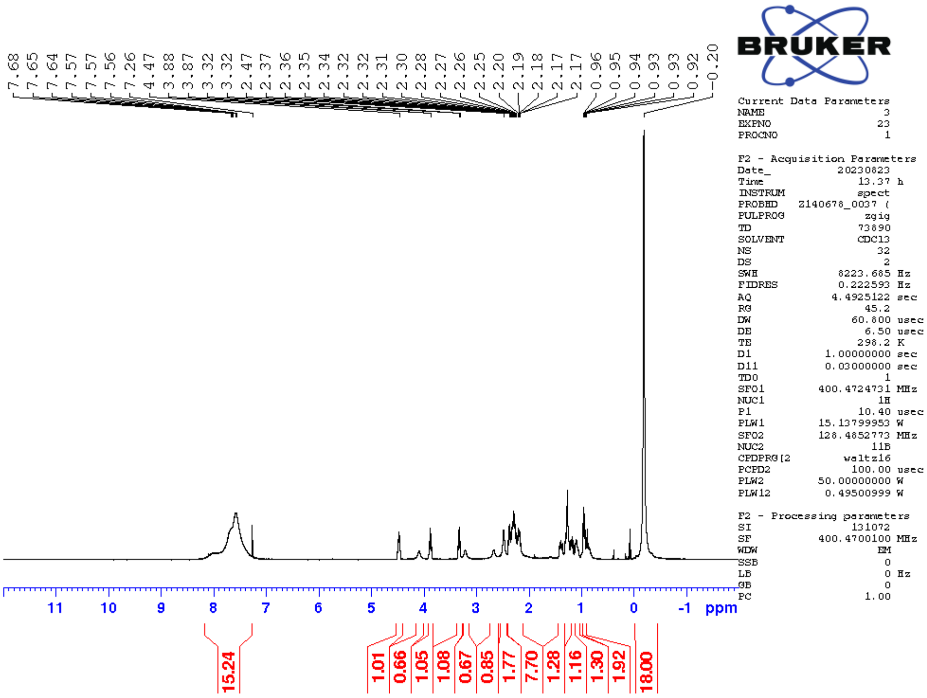

Figure S8. $^{1}H\{^{11}B\}$ NMR spectra of **3** in $CDCl_3$ at 298 K.

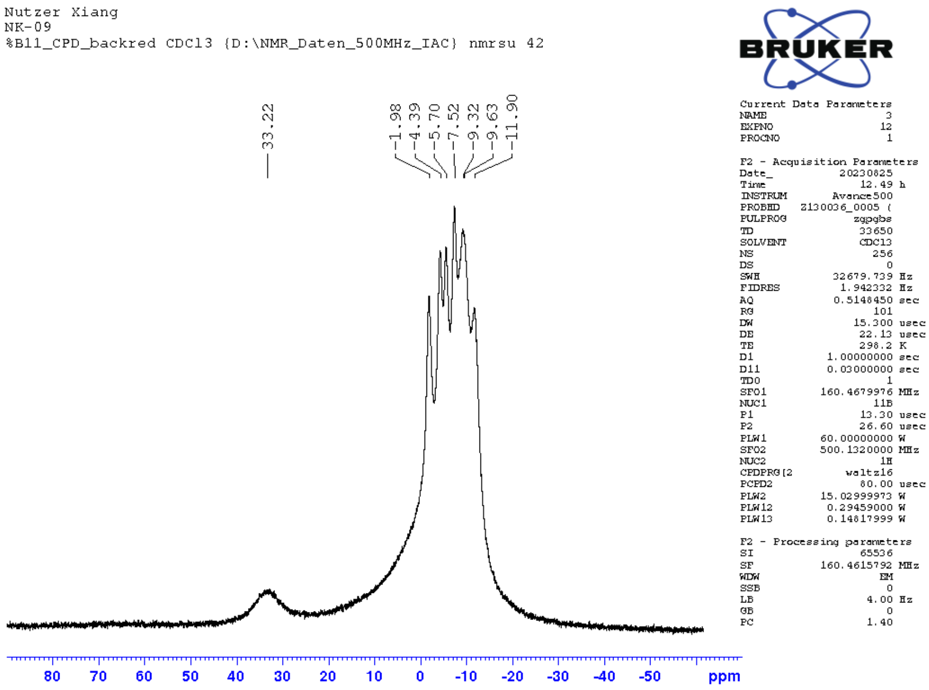

Figure S9. ^{11}B NMR spectra of **3** in $CDCl_3$ at 298 K.

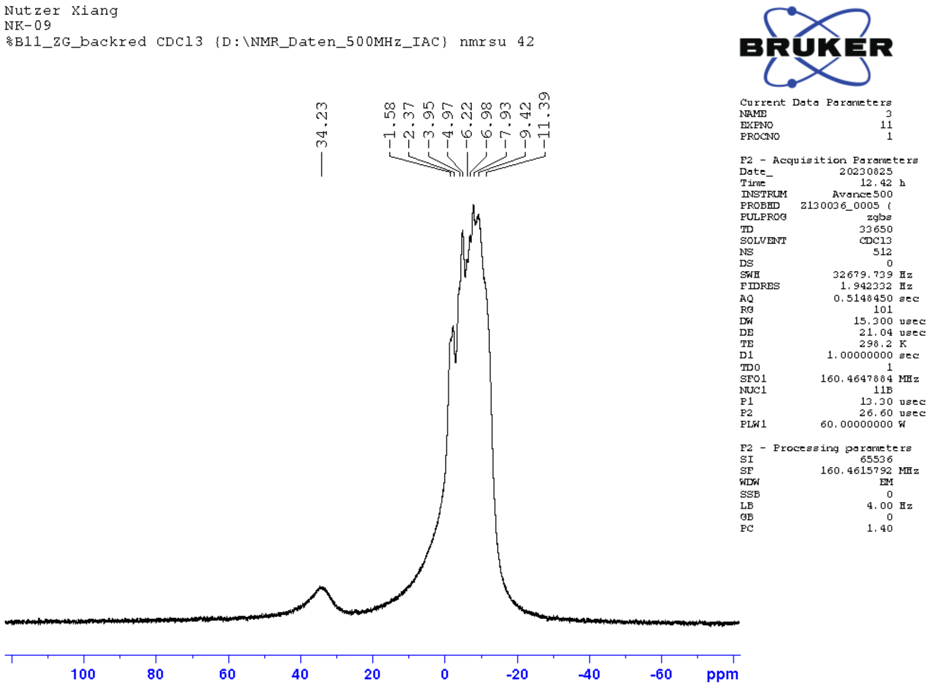

Figure S10. $^{11}B\{^1H\}$ NMR spectra of **3** in $CDCl_3$ at 298 K.

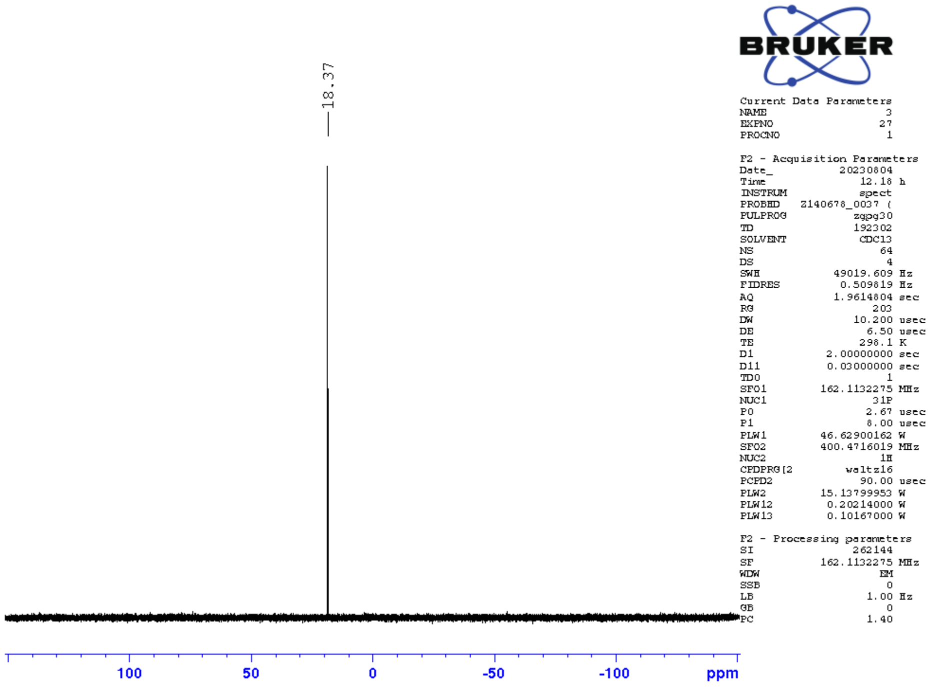

Figure S11. ^{31}P NMR spectra of **3** in $CDCl_3$ at 298 K.

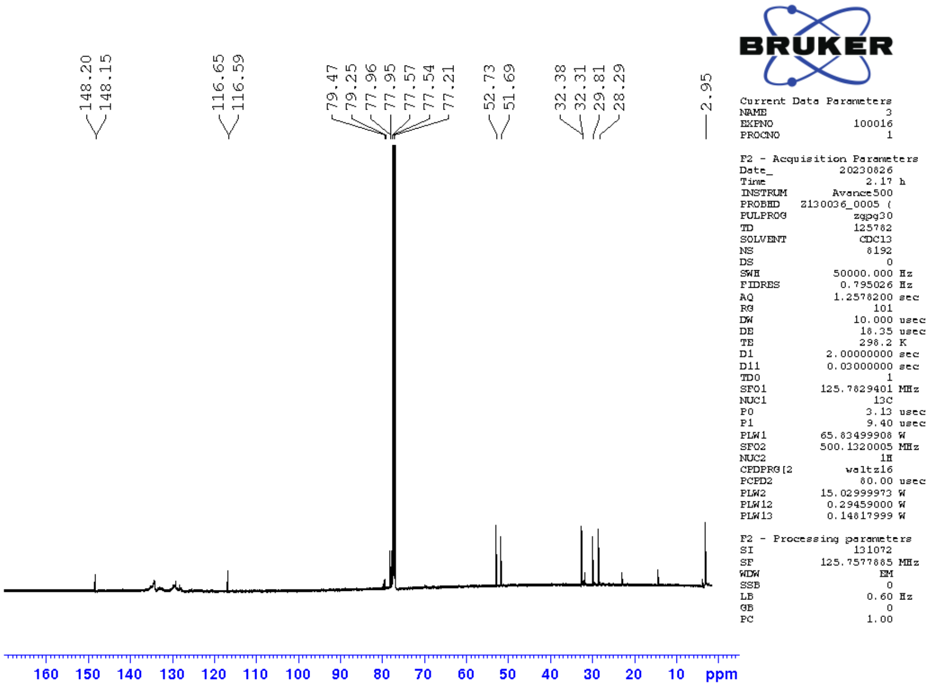

Figure S12. $^{13}C\{^1H\}$ NMR spectra of **3** in $CDCl_3$ at 298 K.

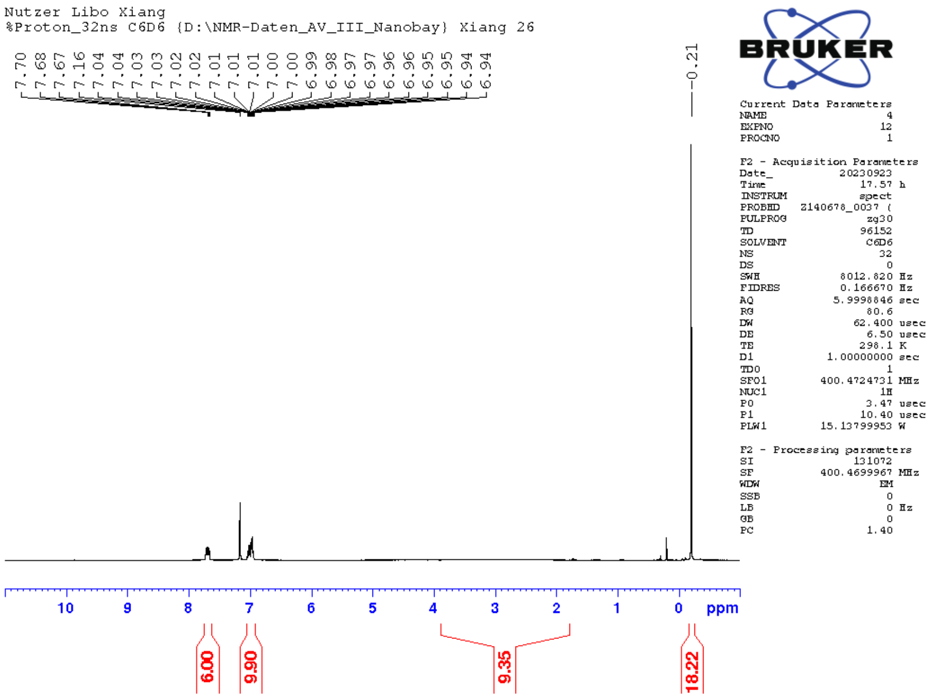

Figure S13. ^{1}H NMR spectra of **4** in C_6D_6 at 298 K.

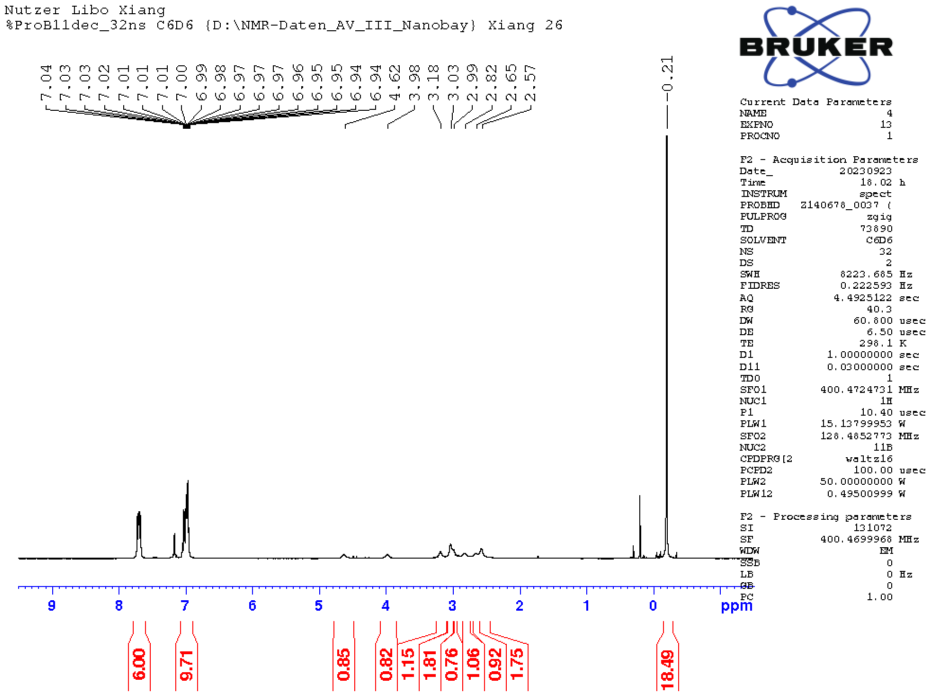

Figure S14. $^{1}H\{^{11}B\}$ NMR spectra of **4** in C_6D_6 at 298 K.

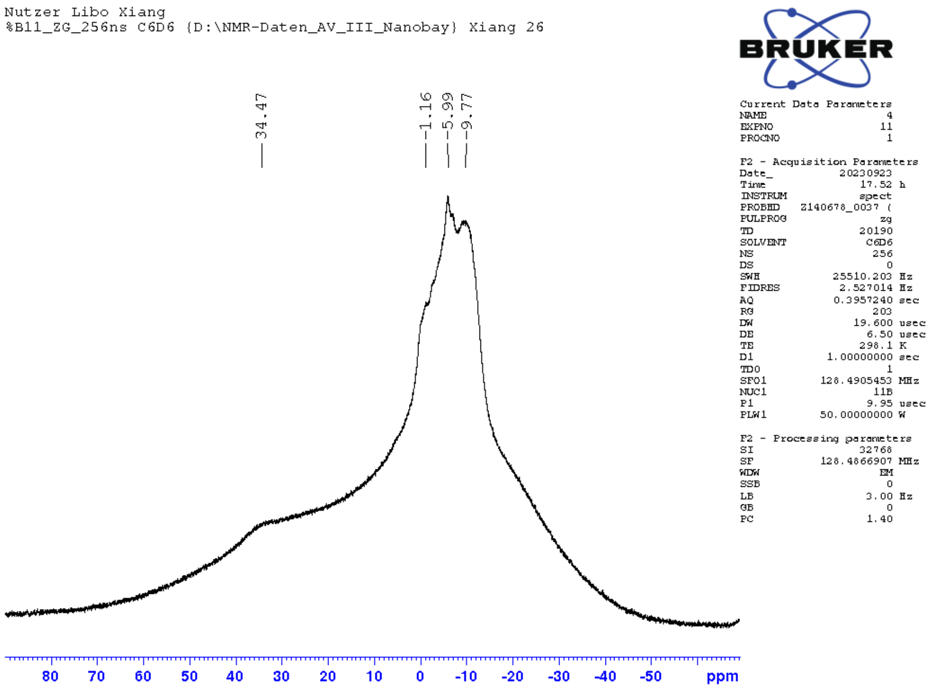

Figure S15. ^{11}B NMR spectra of **4** in C_6D_6 at 298 K.

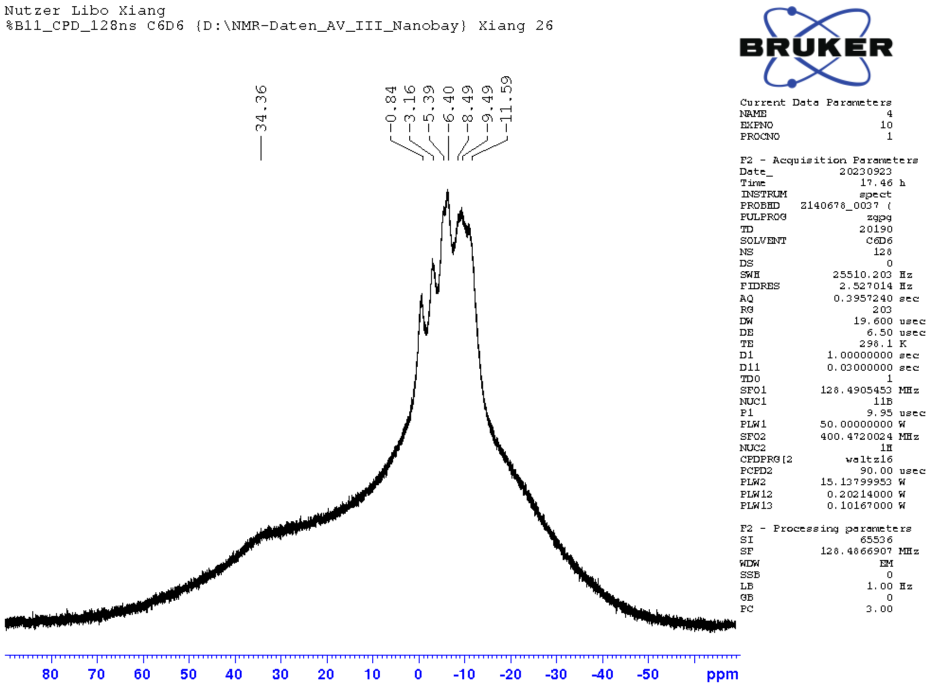

Figure S16. ^{11}B{^{1}H} NMR spectra of **4** in C_6D_6 at 298 K.

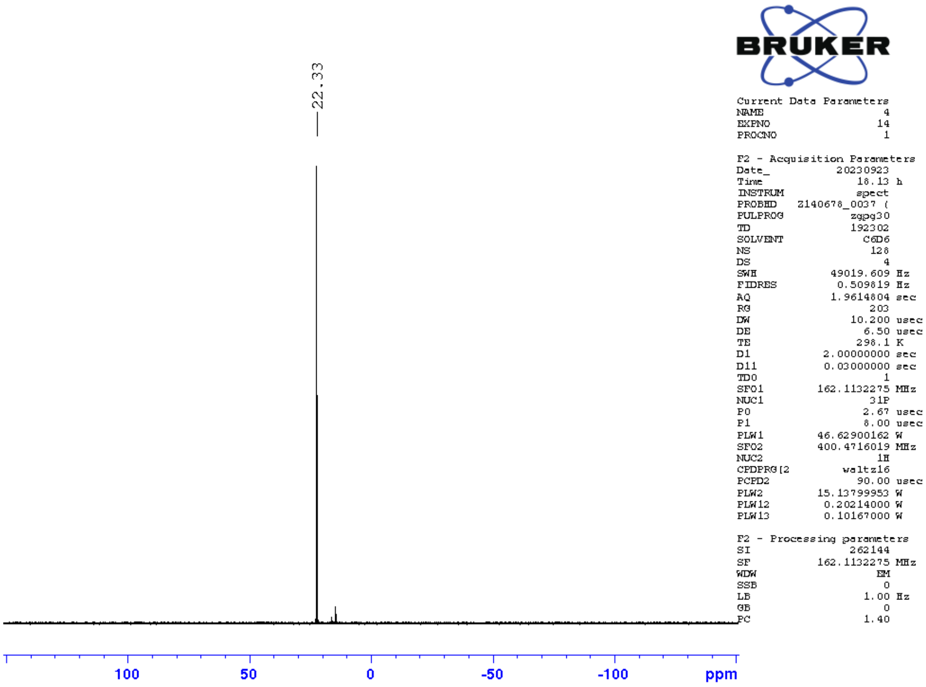

Figure S17. ^{31}P NMR spectra of **4** in C_6D_6 at 298 K.

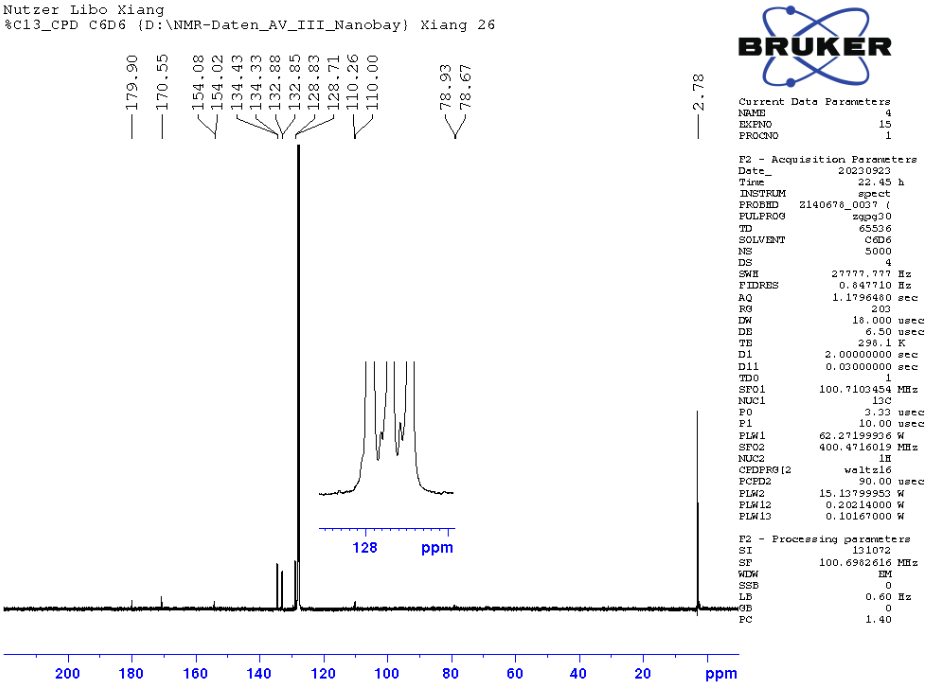

Figure S18. $^{13}C\{^1H\}$ NMR spectra of **4** in C_6D_6 at 298 K.

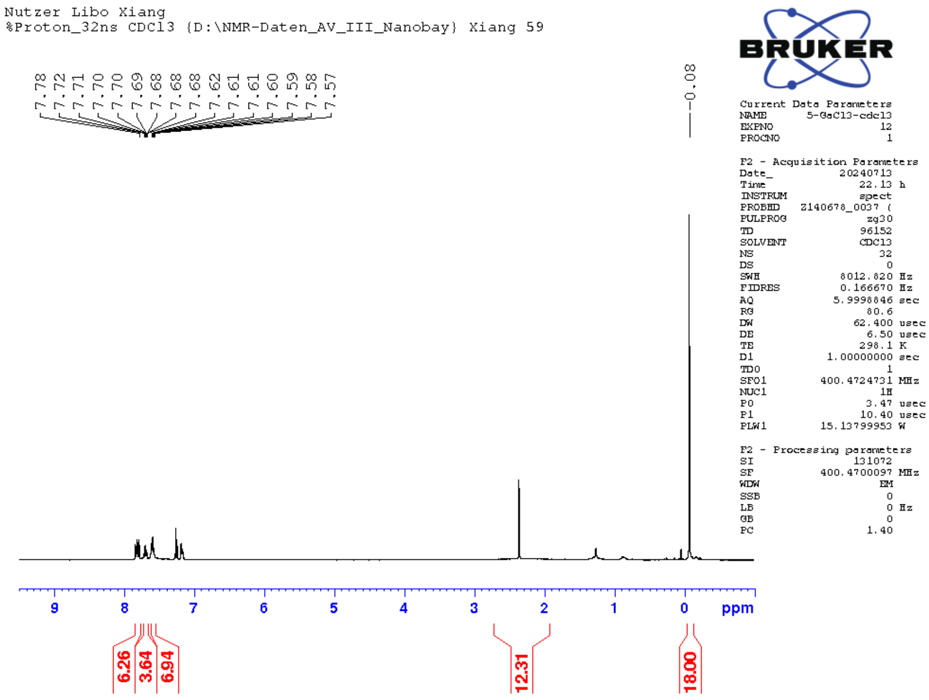

Figure S19. ^{1}H NMR spectra of **5** in $CDCl_3$ at 298 K.

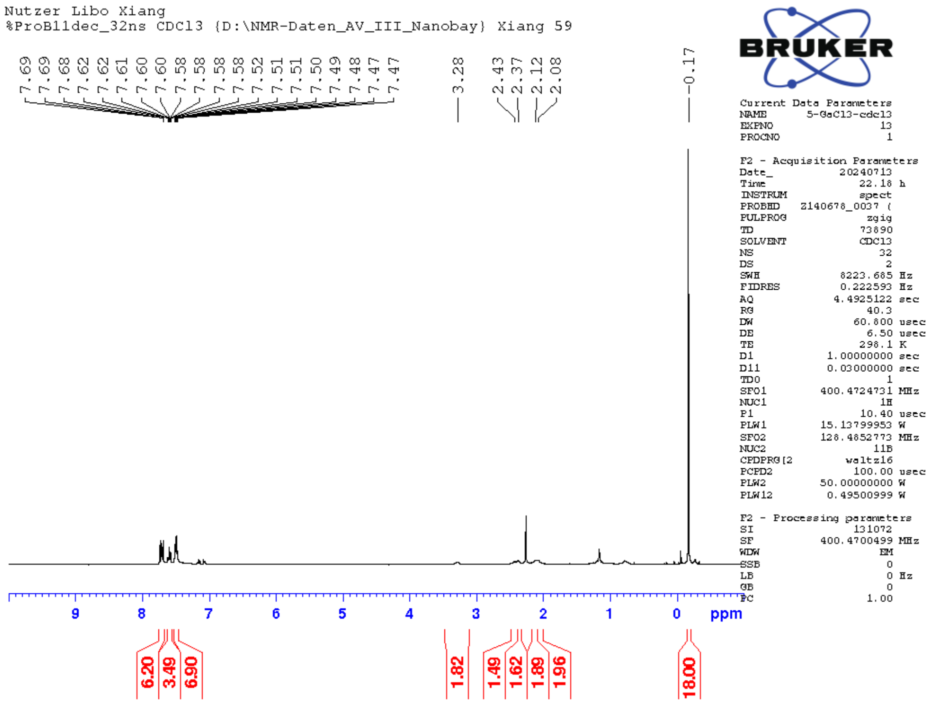

Figure S20. ^{1}H{^{11}B} NMR spectra of **5** in $CDCl_3$ at 298 K.

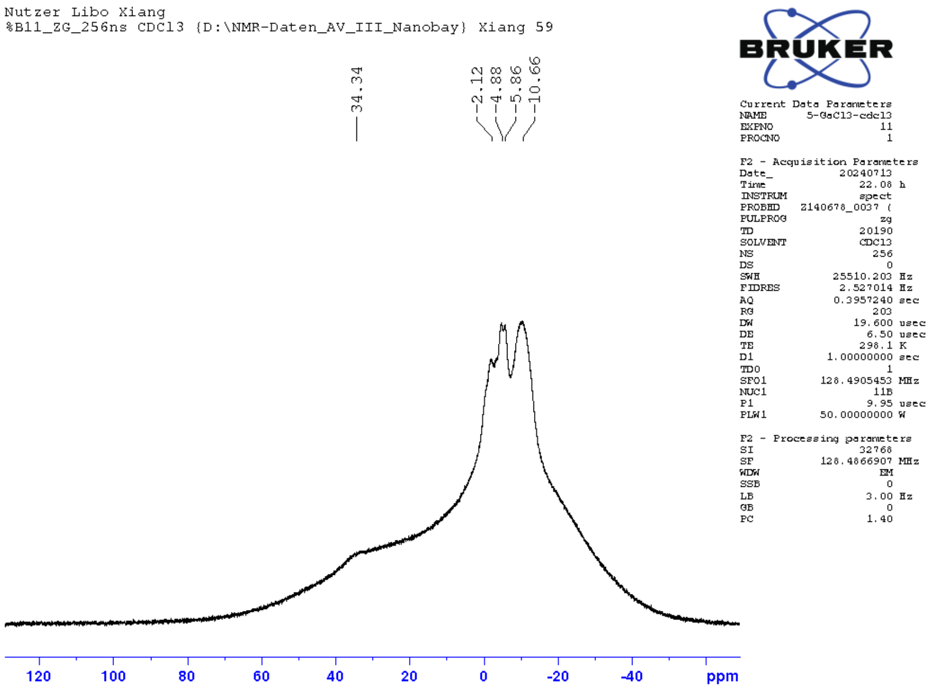

Figure S21. ${}^{11}B$ NMR spectra of **5** in $CDCl_3$ at 298 K.

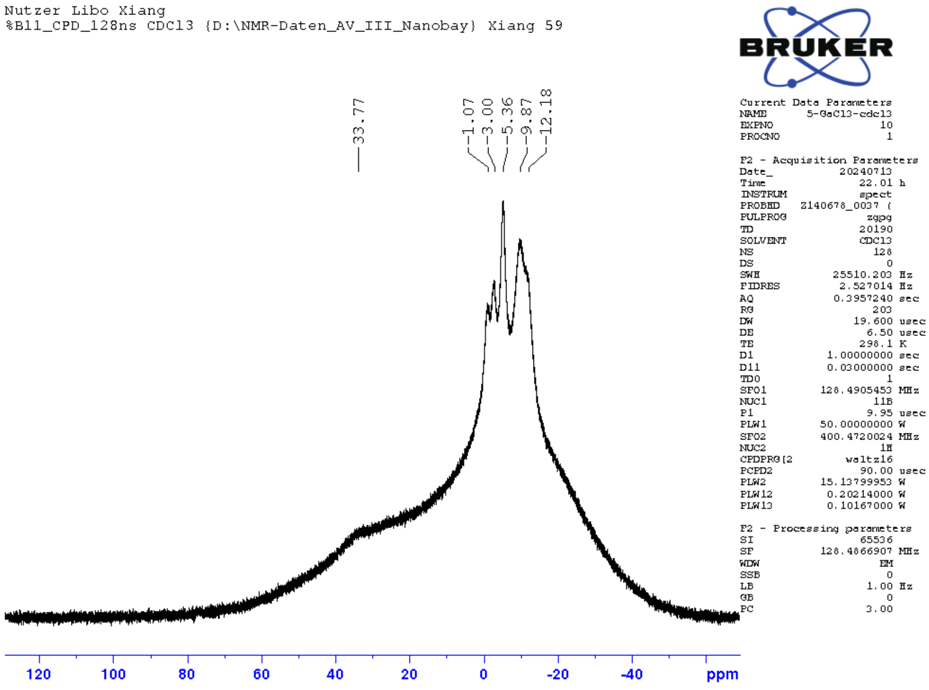

Figure S22. ${}^{11}B\{{}^{1}H\}$ NMR spectra of **5** in $CDCl_3$ at 298 K.

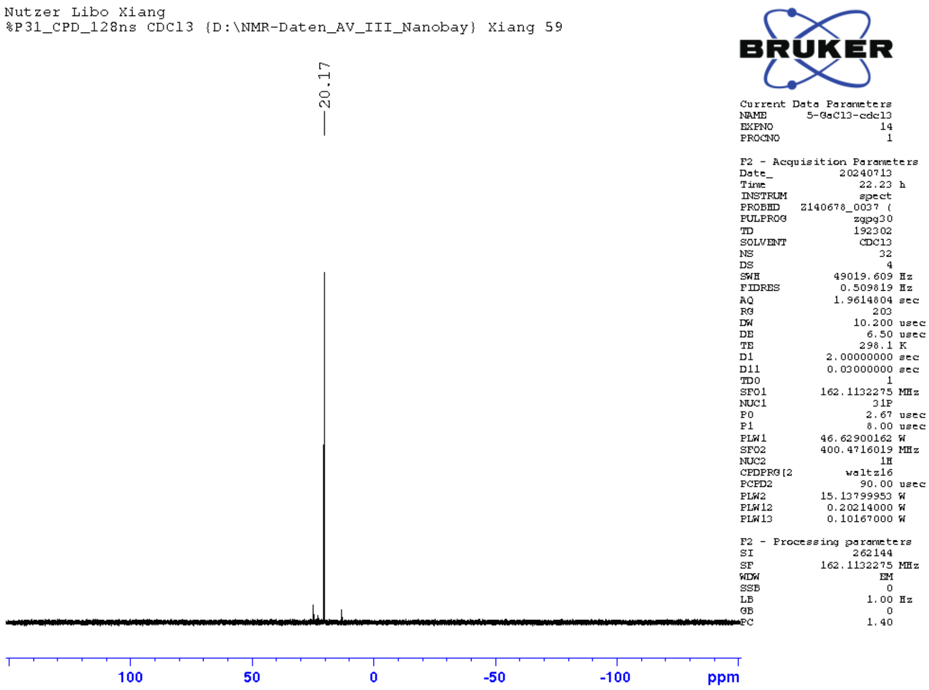

Figure S23. ^{31}P NMR spectra of **5** in $CDCl_3$ at 298 K.

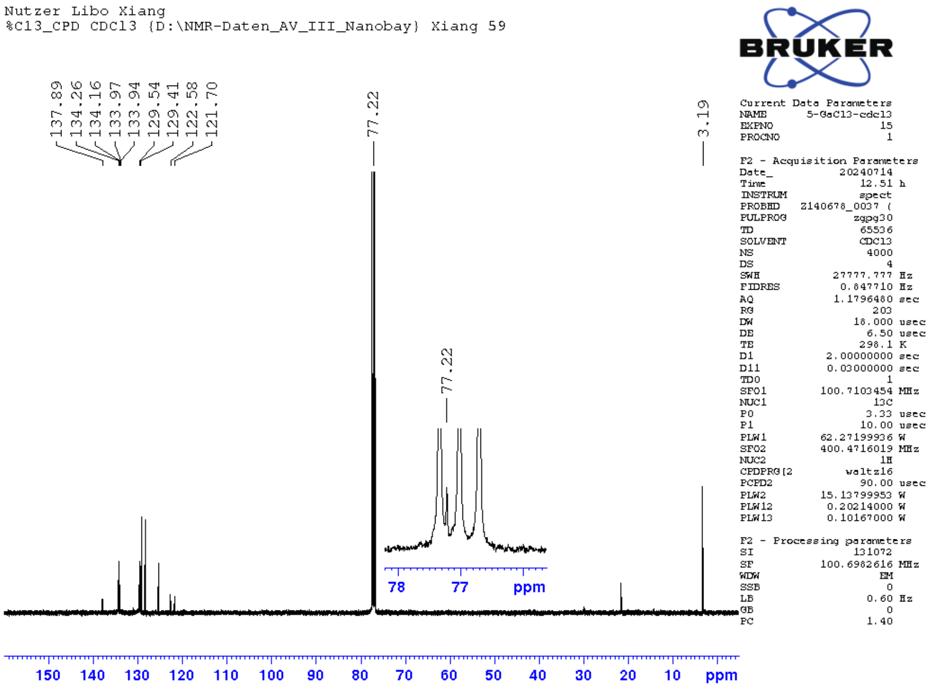

Figure S24. $^{13}C\{^1H\}$ NMR spectra of **5** in $CDCl_3$ at 298 K.

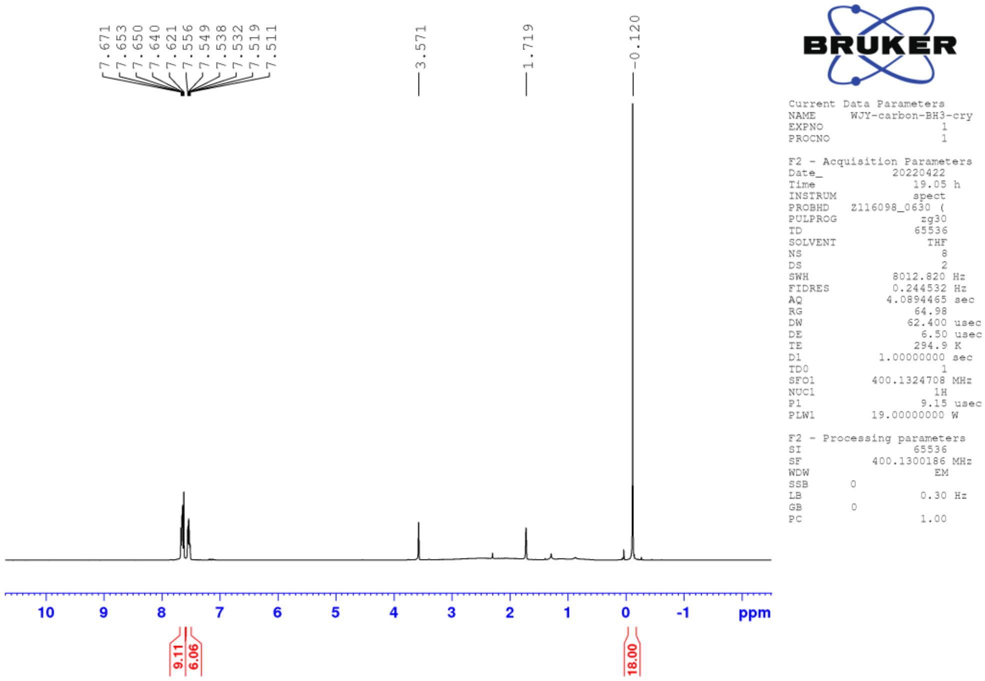

Figure S25. ^{1}H NMR spectra of **6** in THF-d_8 at 298 K.

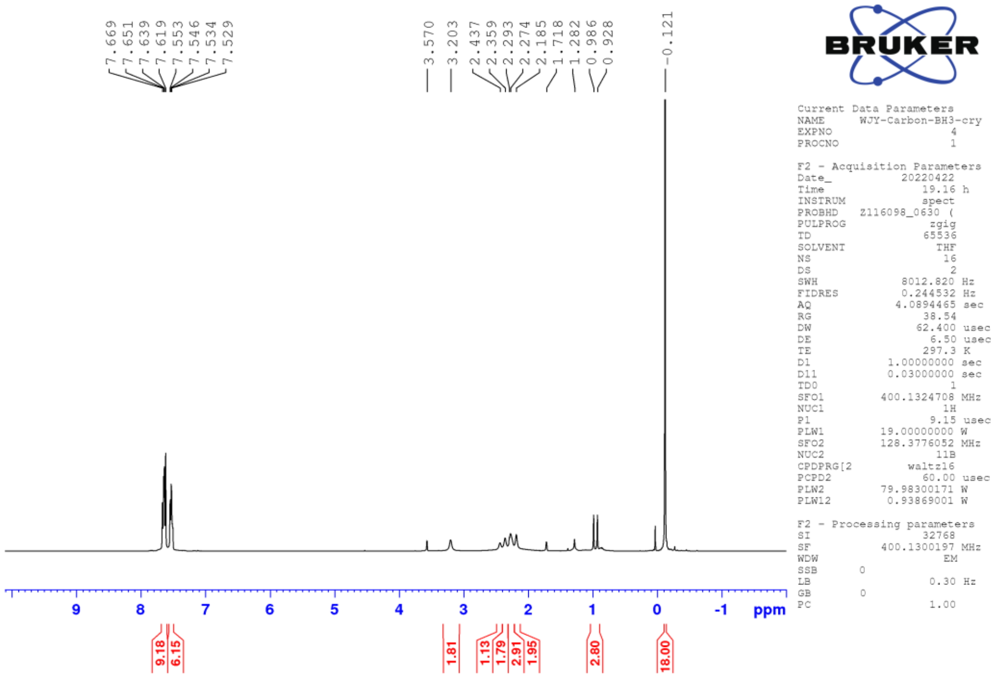

Figure S26. $^{1}H\{^{11}B\}$ NMR spectra of **6** in THF-d_8 at 298 K.

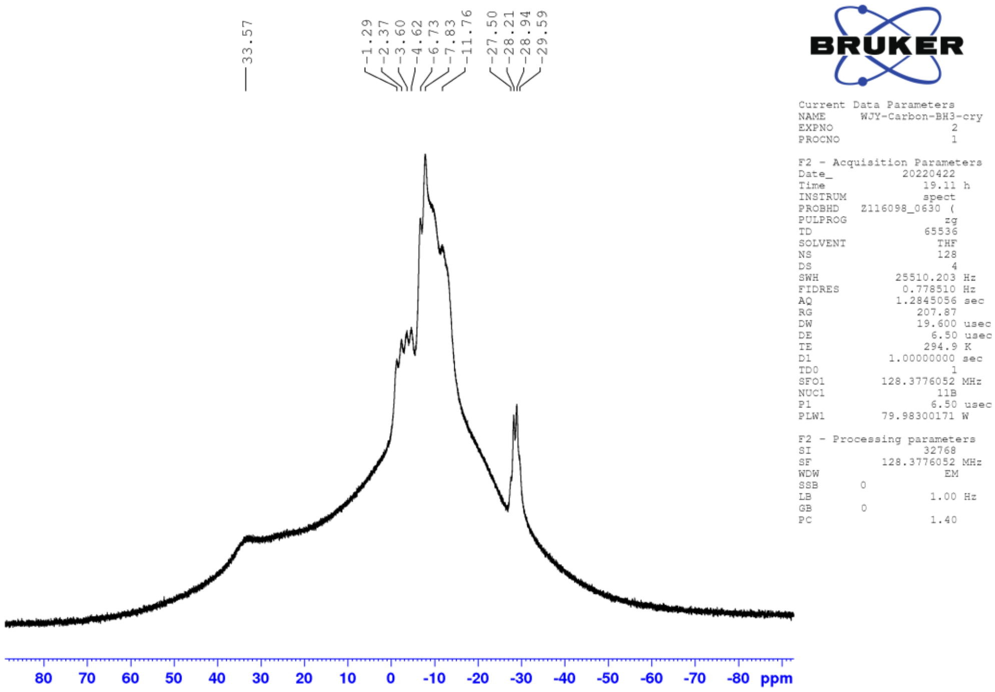

Figure S27. ^{11}B NMR spectra of **6** in THF-d_8 at 298 K.

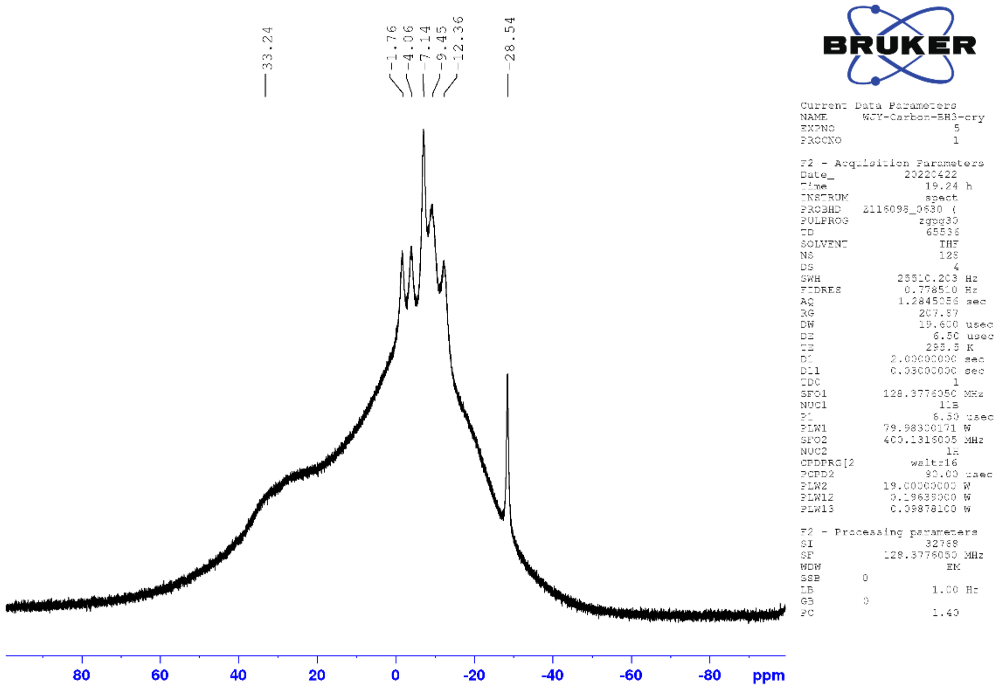

Figure S28. ^{11}B{^{1}H} NMR spectra of **6** in THF-d_8 at 298 K.

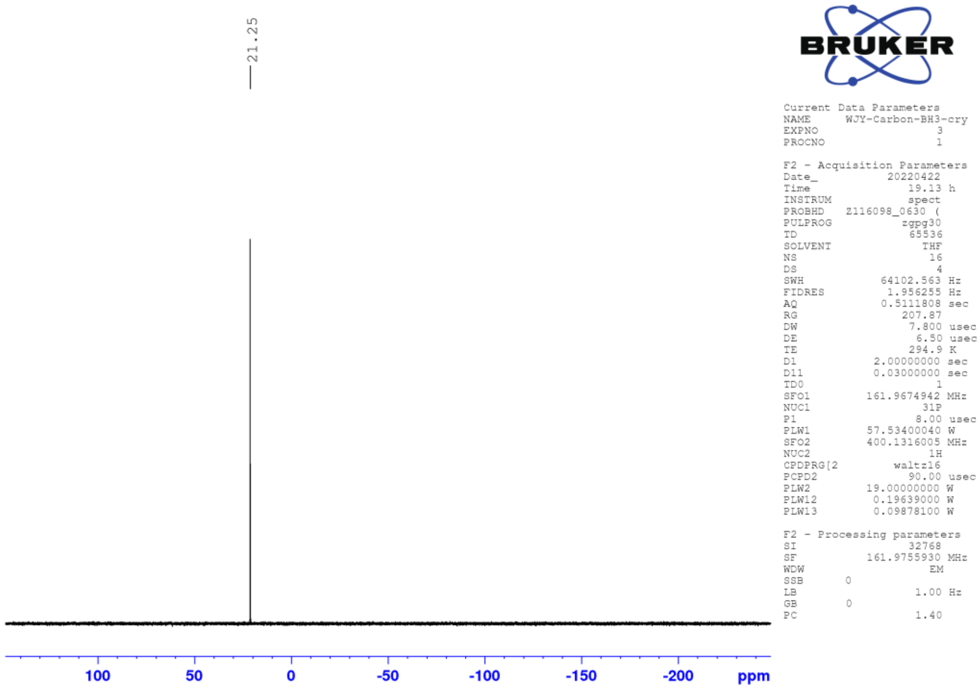

Figure S29. ^{31}P NMR spectra of **6** in THF-d_8 at 298 K.

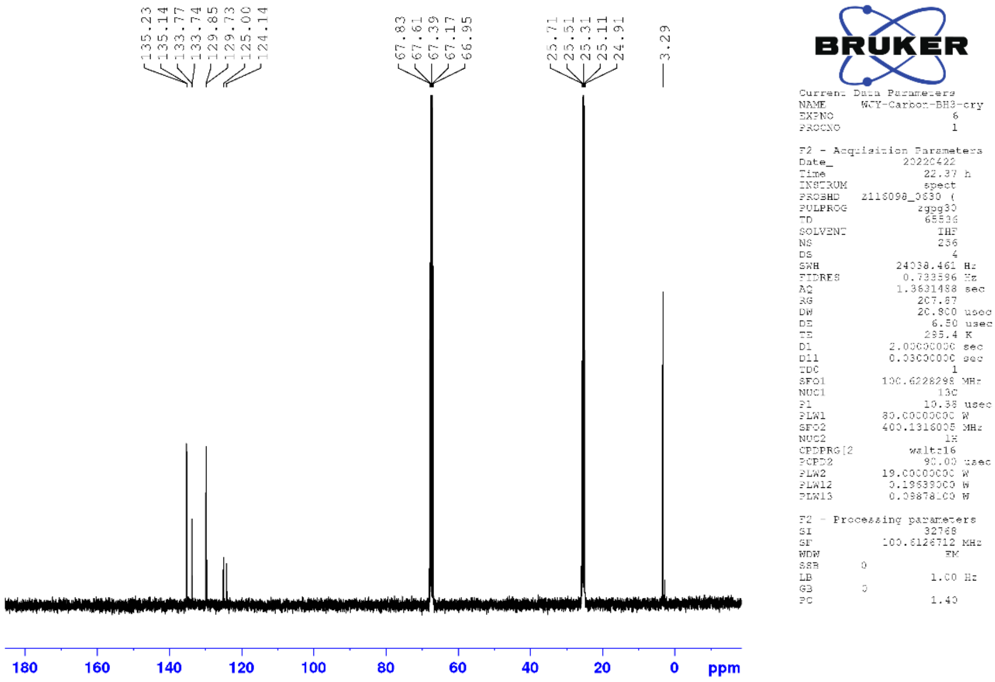

Figure S30. ^{13}C{^{1}H} NMR spectra of **6** in THF-d_8 at 298 K.

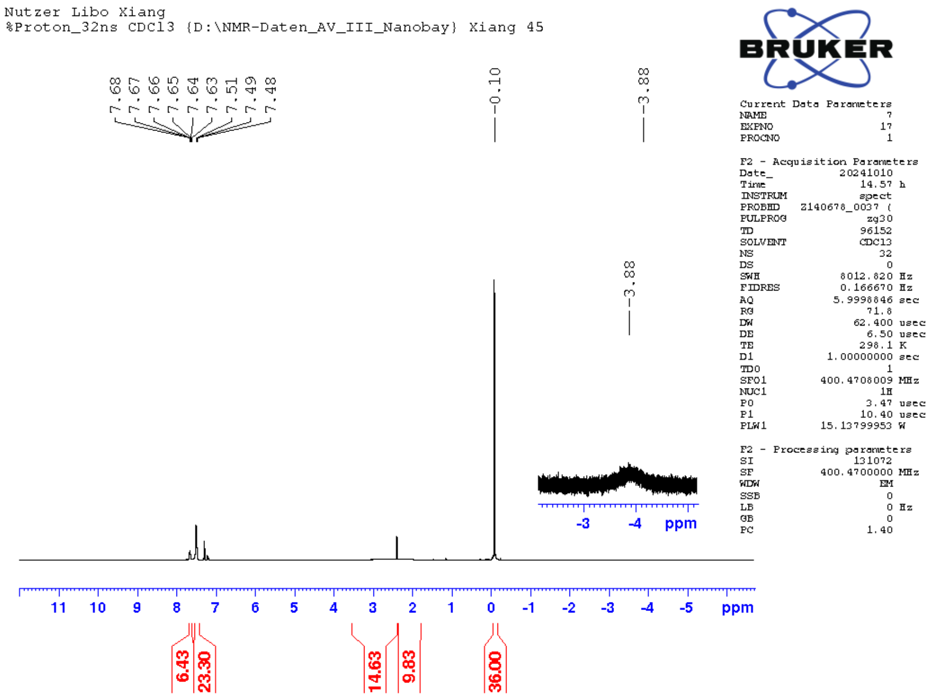

Figure S31. ^{1}H NMR spectra of **7** in $CDCl_3$ at 298 K.

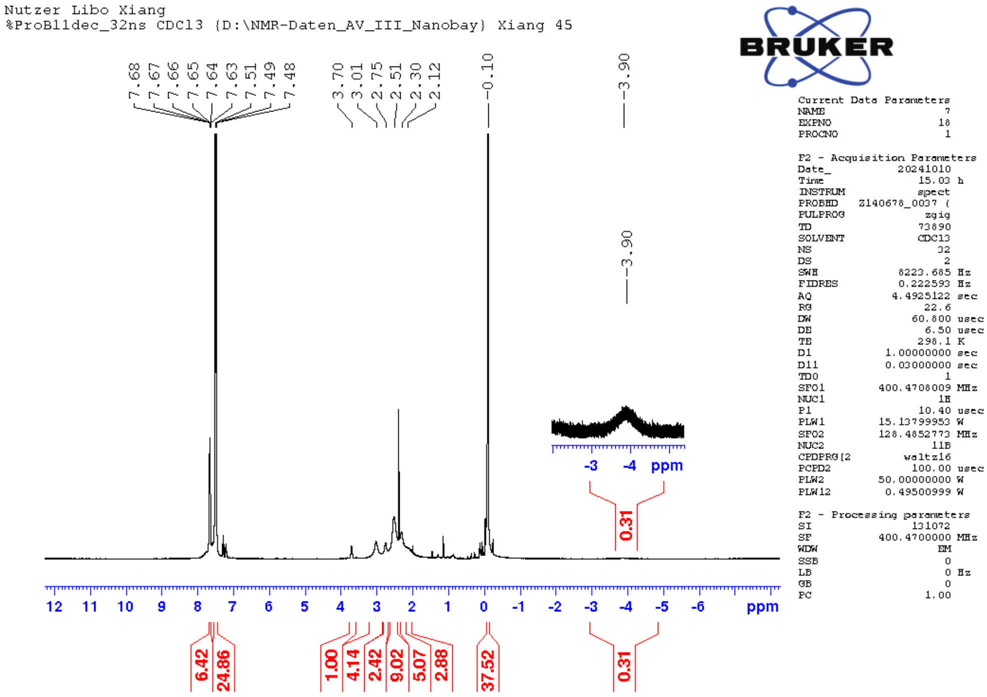

Figure S32. $^{1}H\{^{11}B\}$ NMR spectra of **7** in $CDCl_3$ at 298 K.

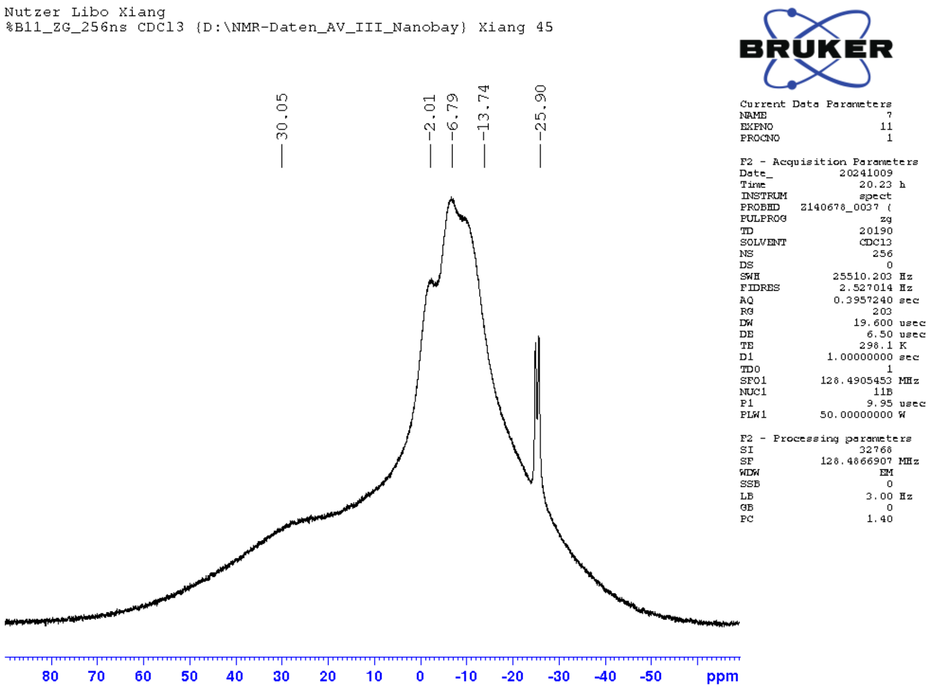

Figure S33. ^{11}B NMR spectra of **7** in $CDCl_3$ at 298 K.

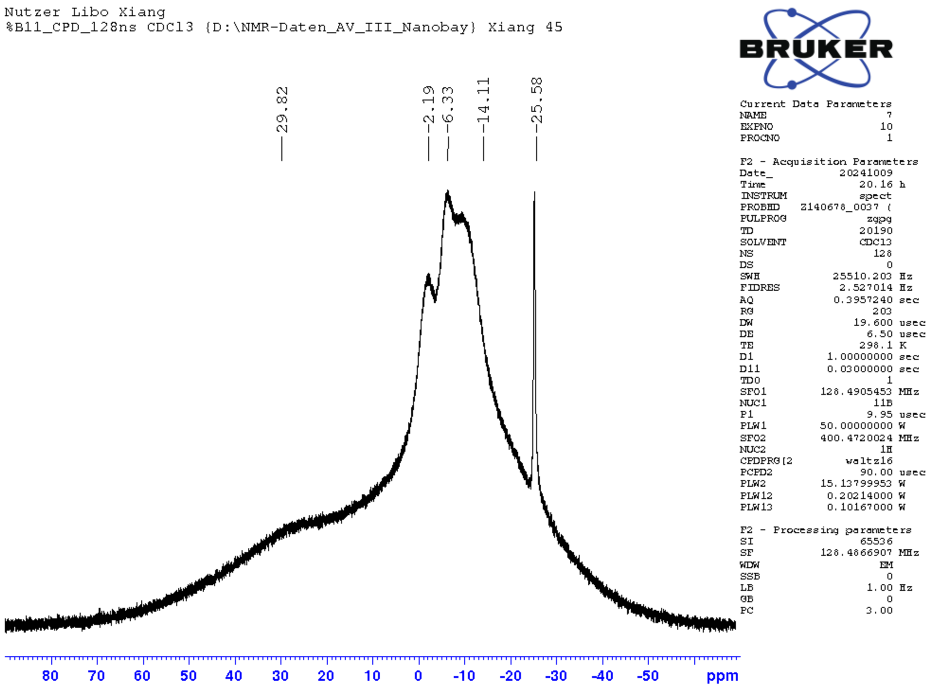

Figure S34. $^{11}B\{^1H\}$ NMR spectra of **7** in $CDCl_3$ at 298 K.

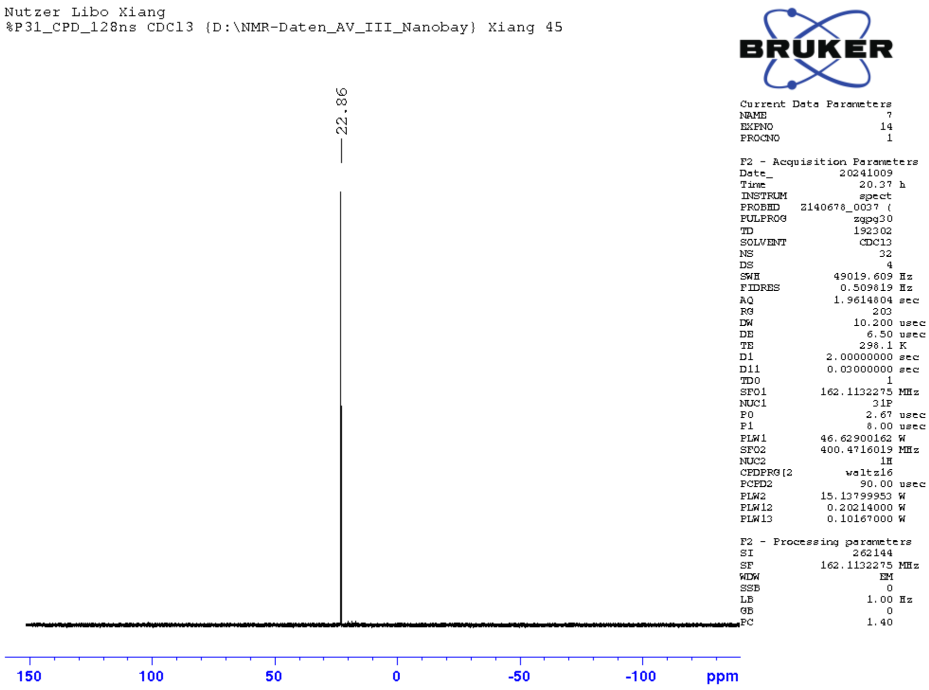

Figure S35. ^{31}P NMR spectra of **7** in $CDCl_3$ at 298 K.

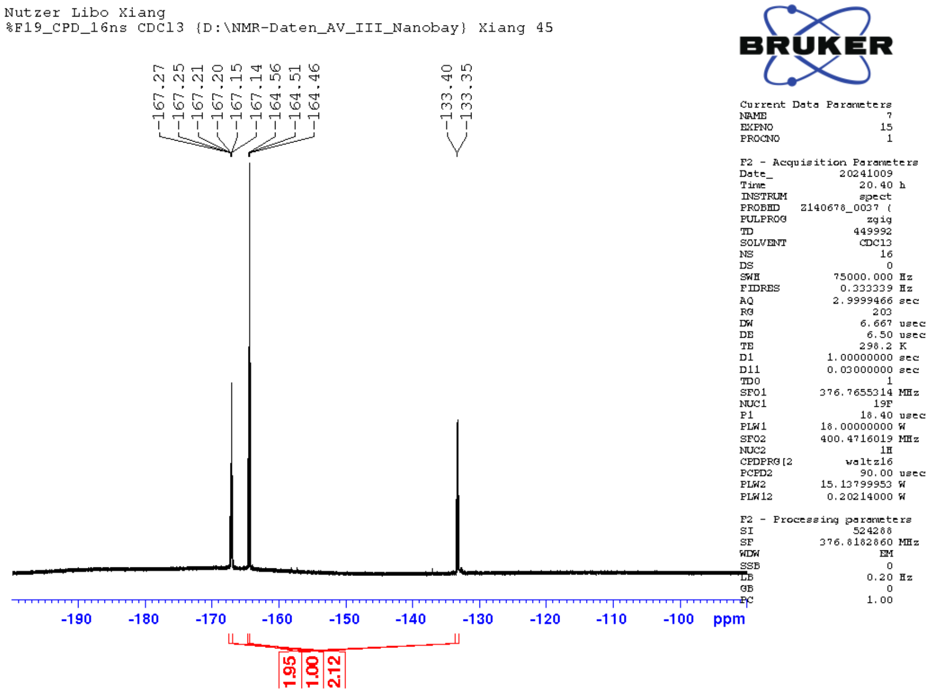

Figure S36. ^{19}F NMR spectra of **7** in $CDCl_3$ at 298 K.

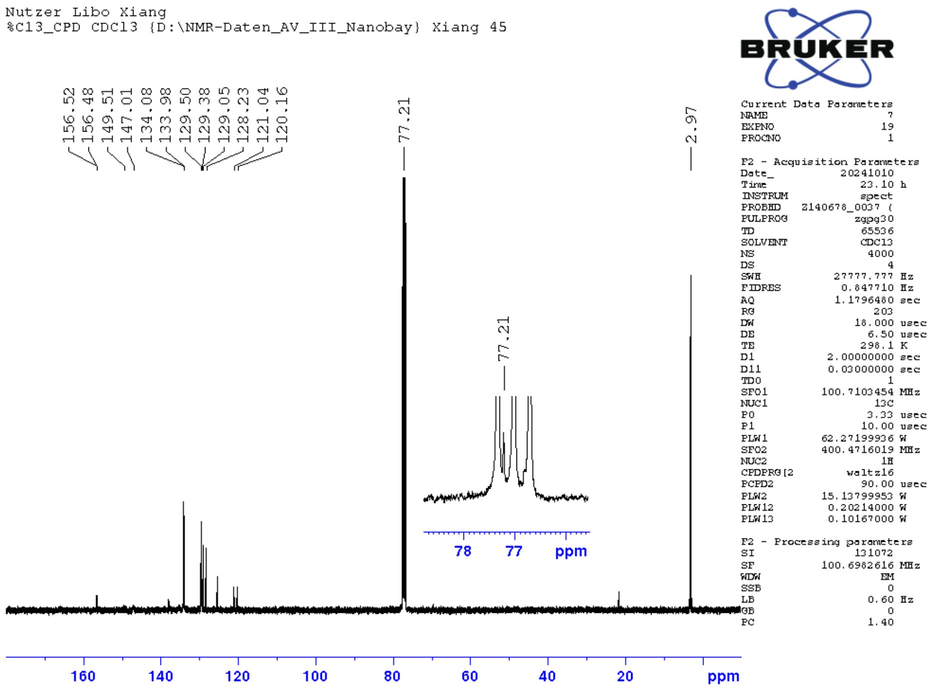

Figure S37. $^{13}C\{^1H\}$ NMR spectra of **7** in $CDCl_3$ at 298 K.

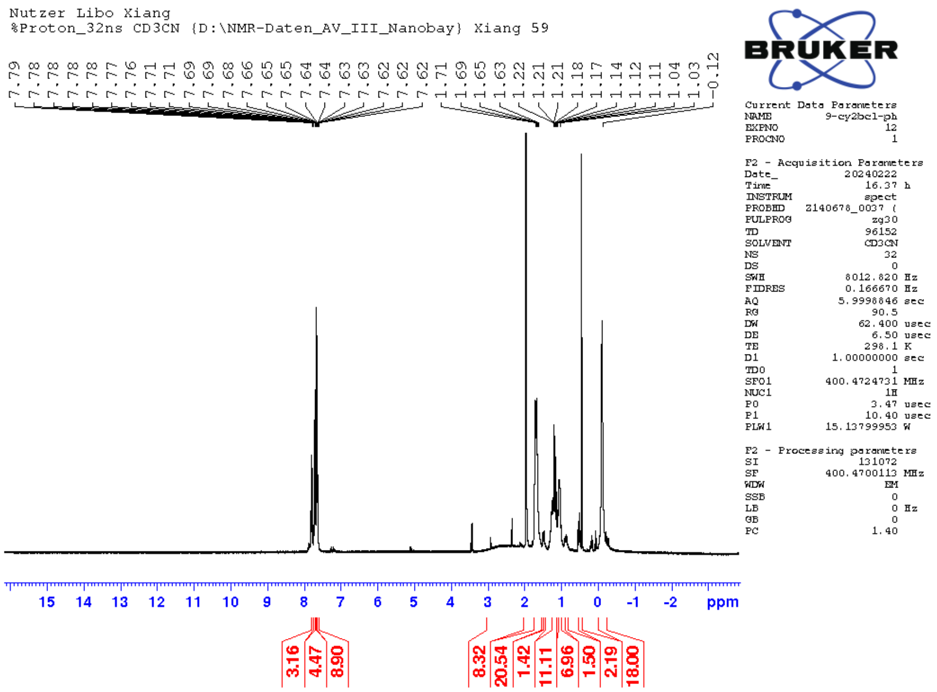

Figure S38. ^1H NMR spectra of **8** in CD_3CN at 298 K.

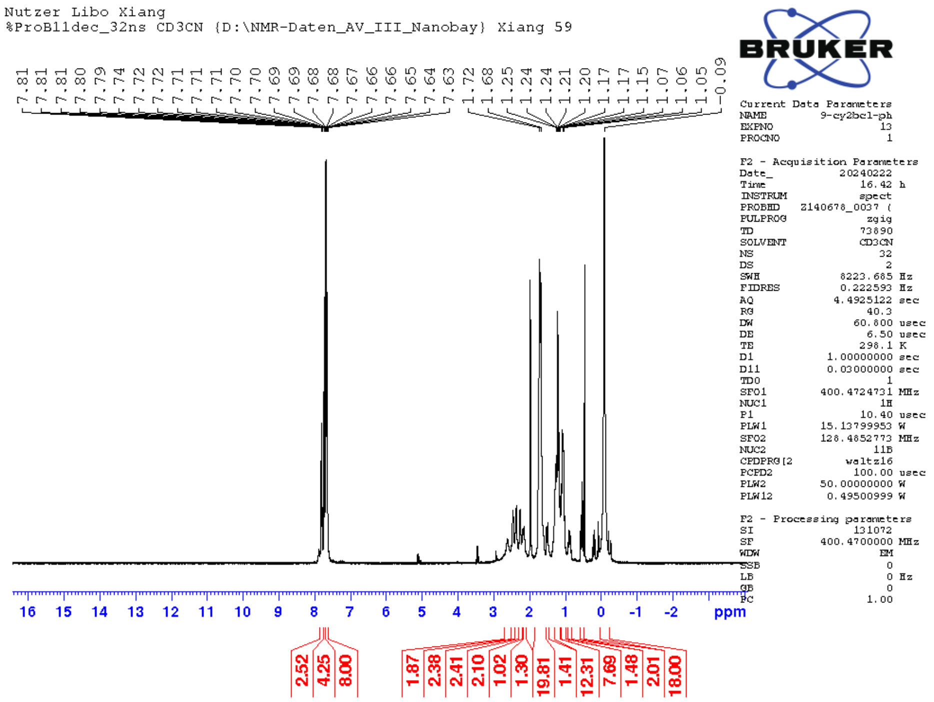

Figure S39. $^{1}H\{^{11}B\}$ NMR spectra of **8** in CD_3CN at 298 K.

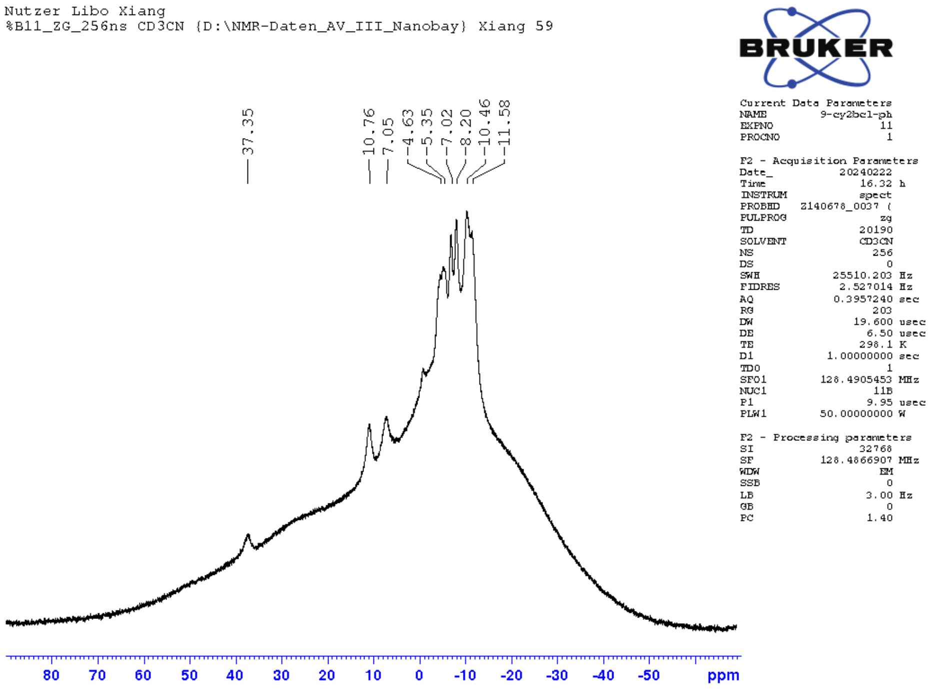

Figure S40. ^{11}B NMR spectra of **8** in CD_3CN at 298 K.

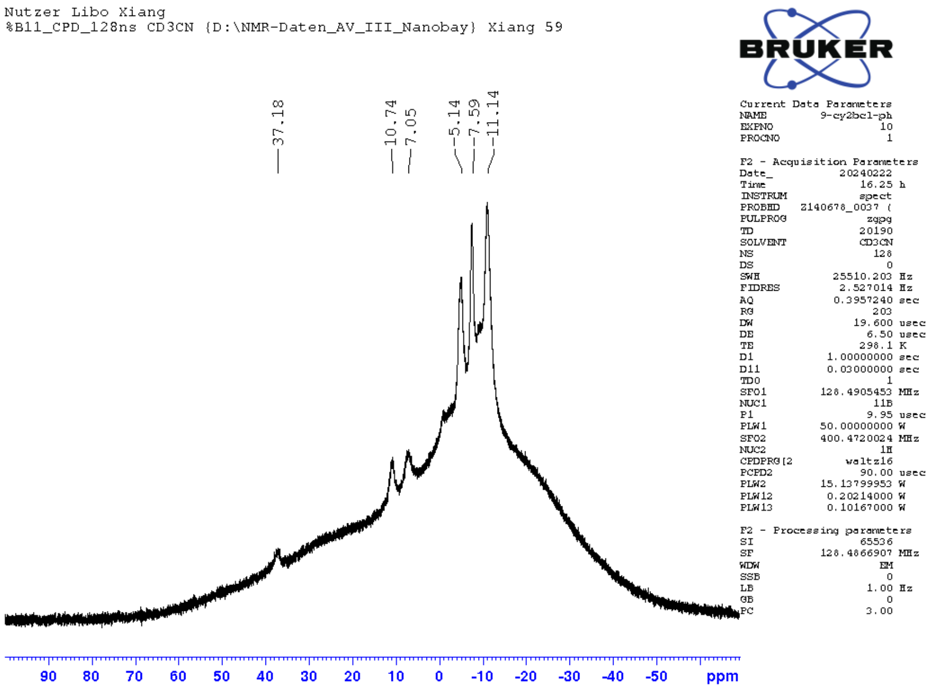

Figure S41. $^{11}B\{^{1}H\}$ NMR spectra of **8** in CD_3CN at 298 K.

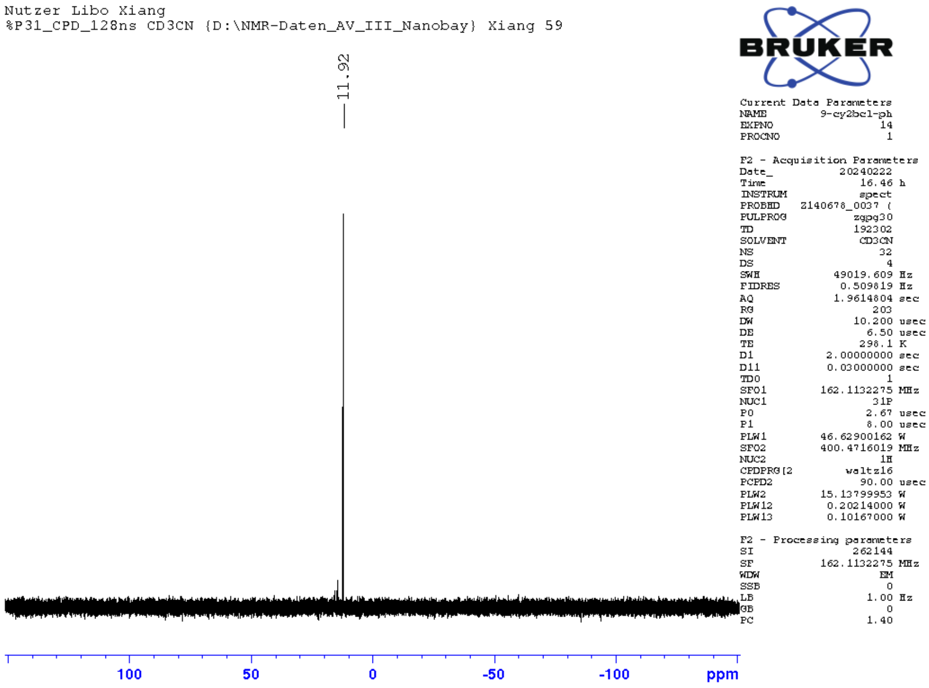

Figure S42. ^{31}P NMR spectra of **8** in CD_3CN at 298 K.

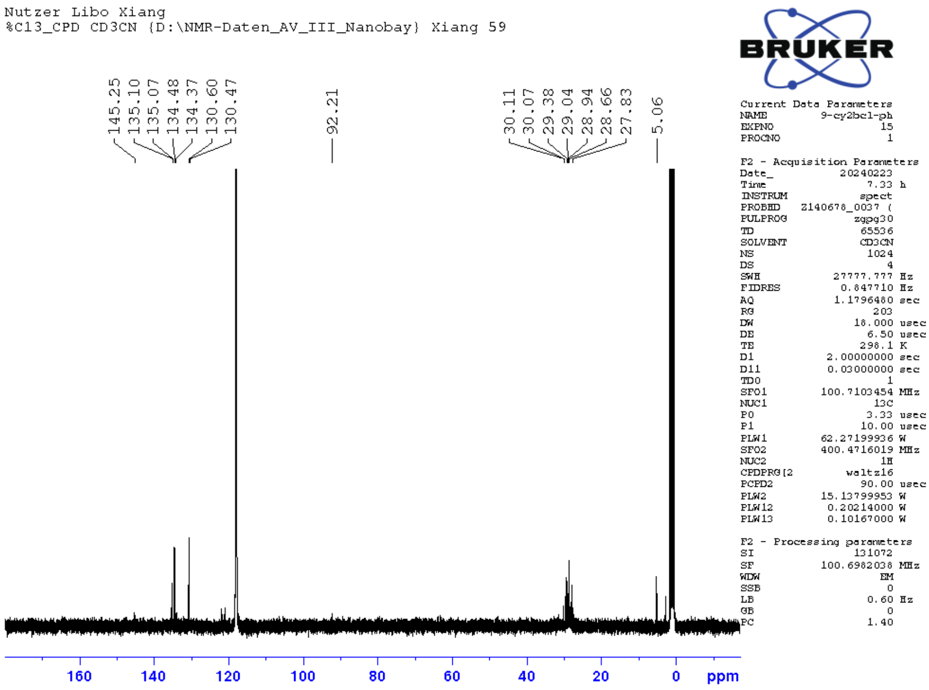

Figure S43. $^{13}C\{^1H\}$ NMR spectra of **8** in CD_3CN at 298 K.

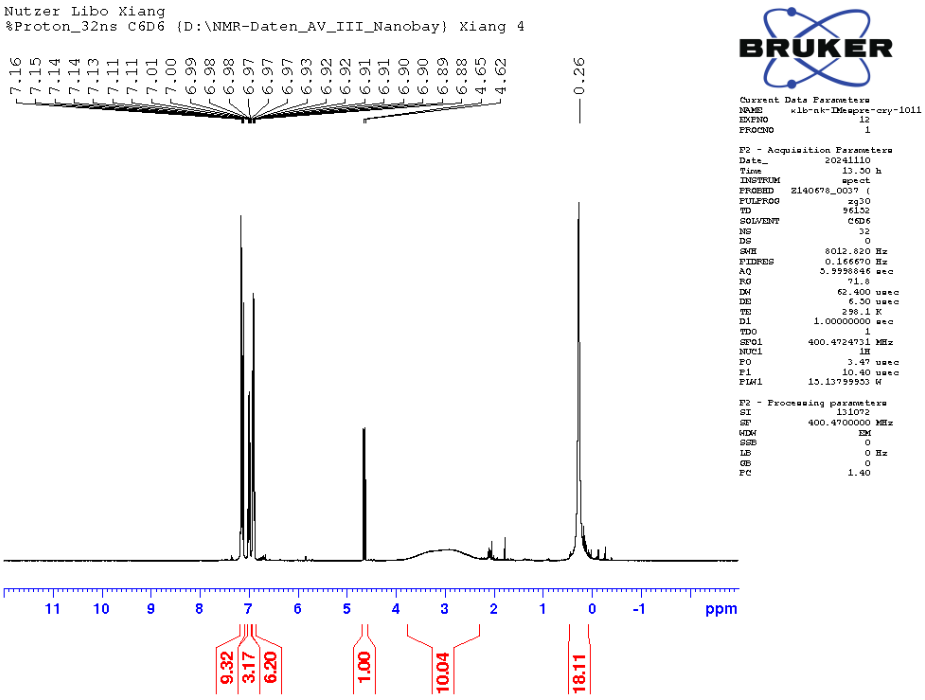

Figure S44. ^1H NMR spectra of **9** in C_6D_6 at 298 K.

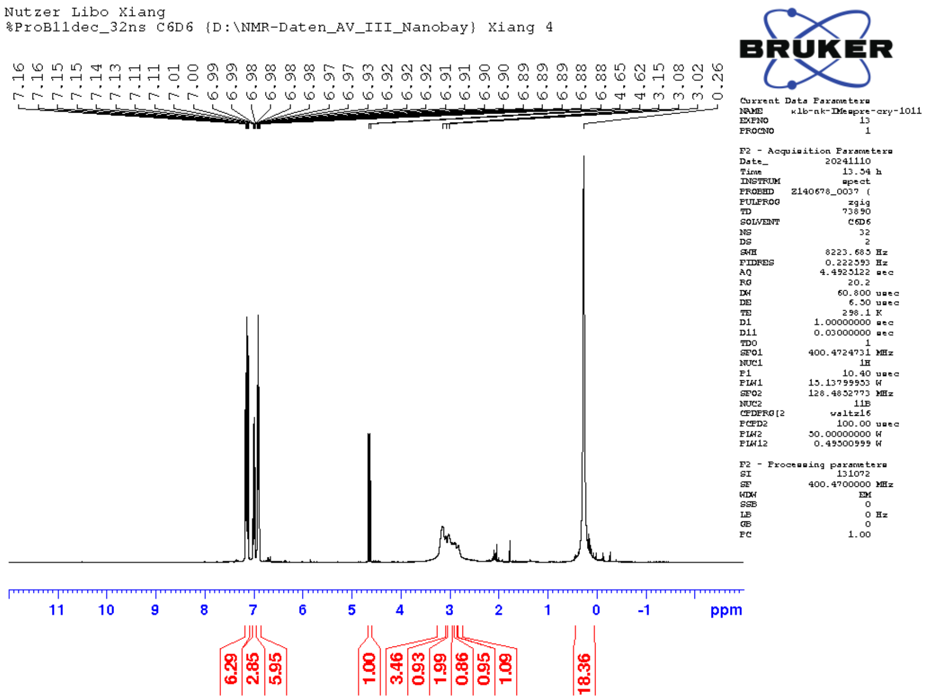

Figure S45. $^{1}H\{^{11}B\}$ NMR spectra of **9** in C_6D_6 at 298 K.

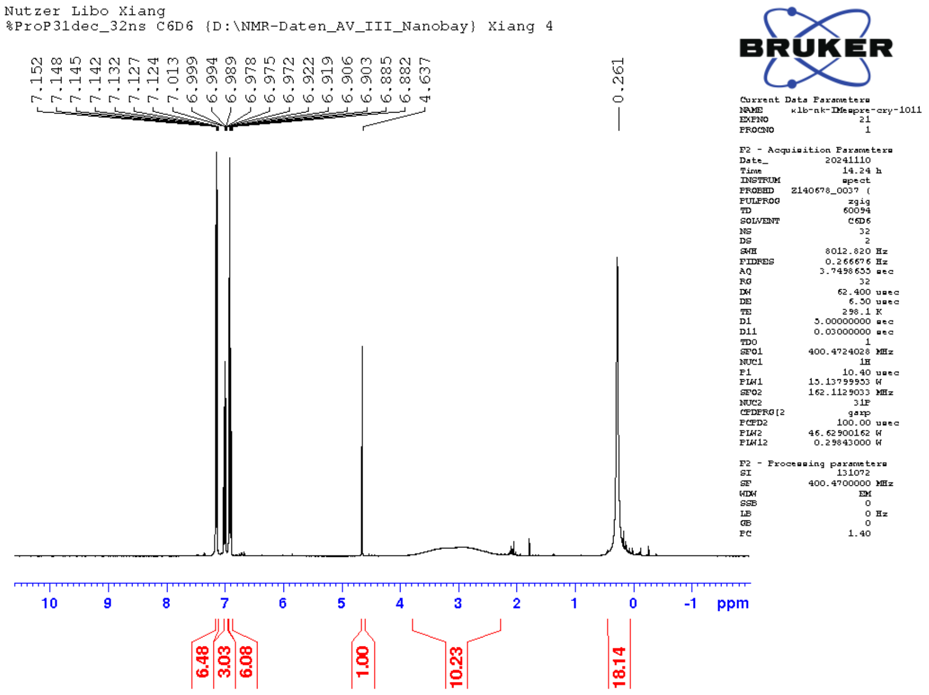

Figure S46. $^{1}H\{^{31}P\}$ NMR spectra of **9** in C_6D_6 at 298 K.

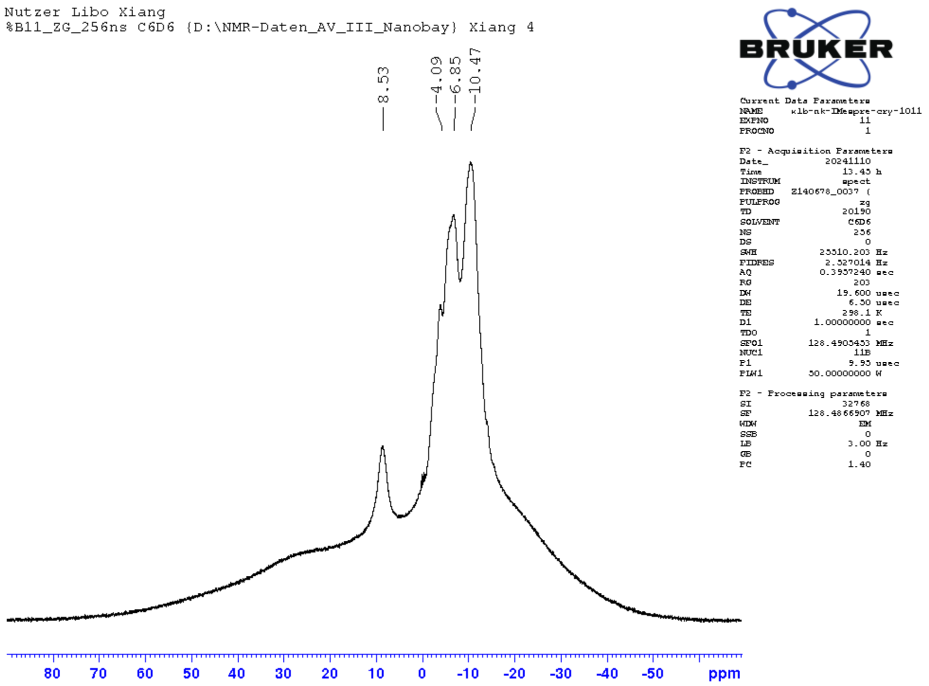

Figure S47. ^{11}B NMR spectra of **9** in C_6D_6 at 298 K.

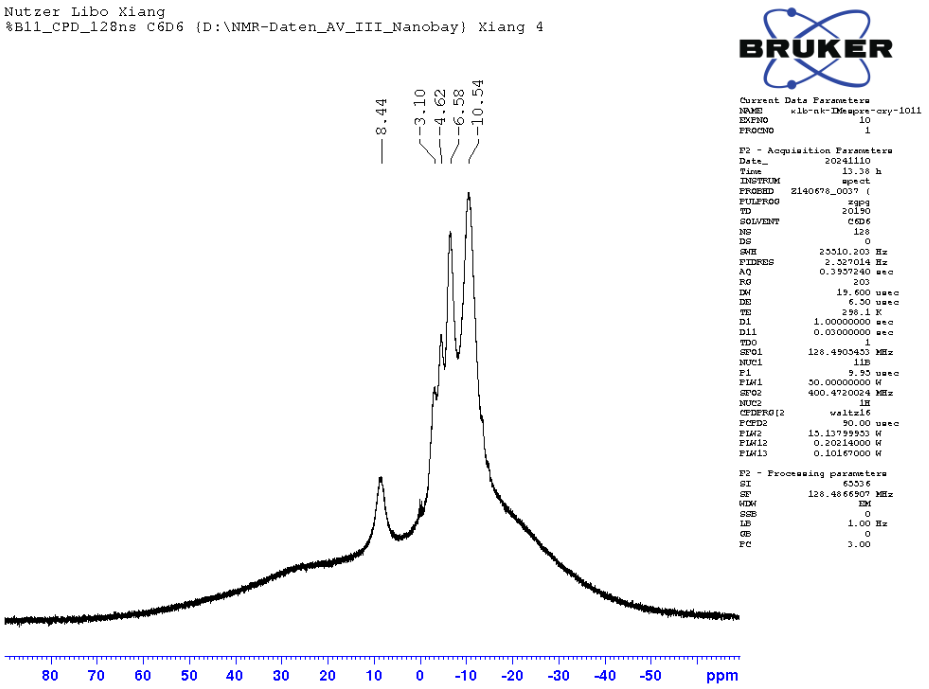

Figure S48. $^{11}B\{^1H\}$ NMR spectra of **9** in C_6D_6 at 298 K.

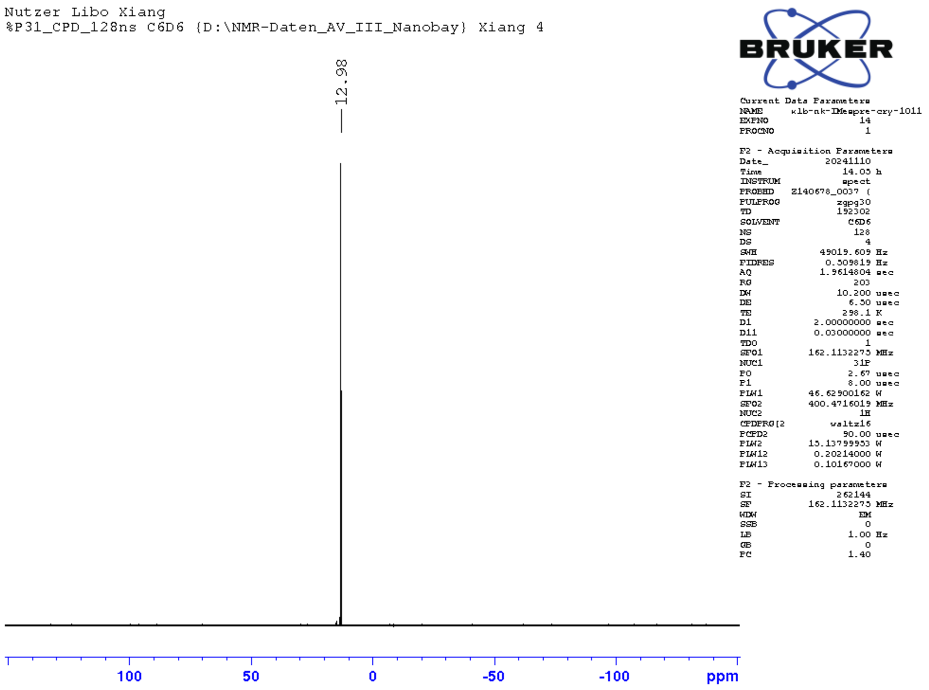

Figure S49. ^{31}P NMR spectra of **9** in C_6D_6 at 298 K.

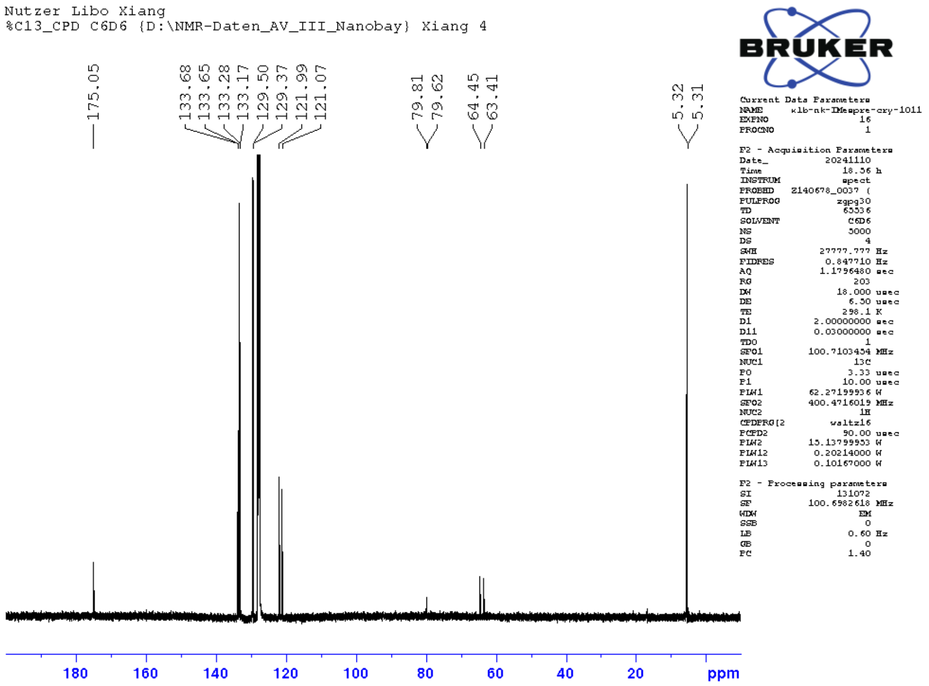

Figure S50. ^{13}C{^{1}H} NMR spectra of **9** in C_6D_6 at 298 K.

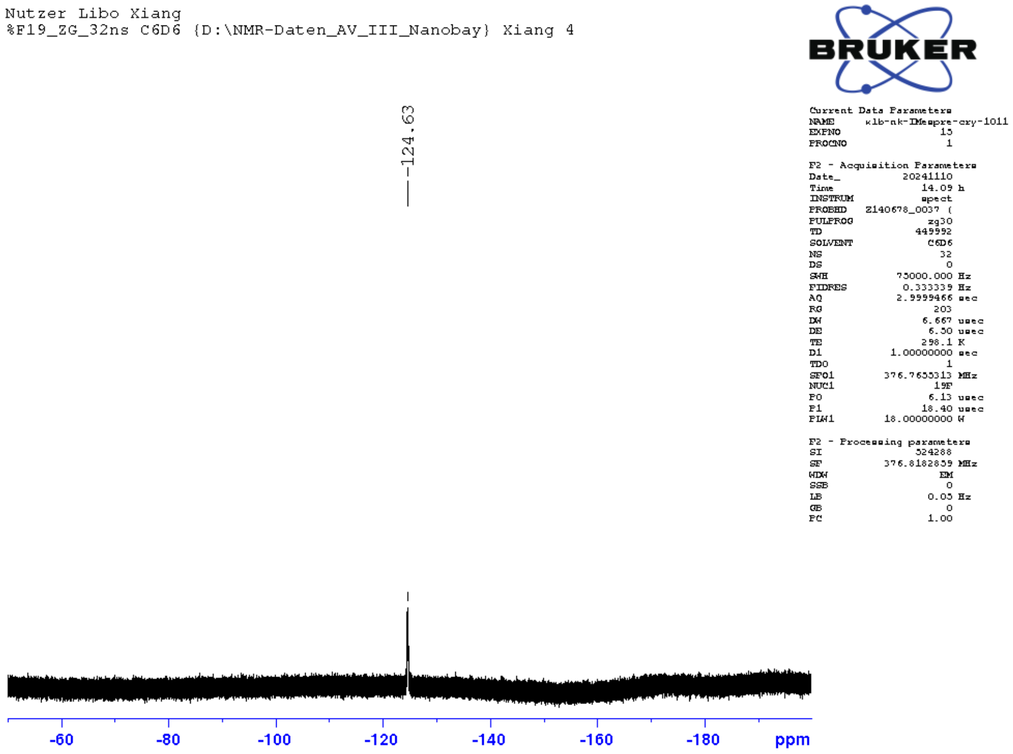

Figure S51. ${}^{19}F\{{}^{1}H\}$ NMR spectra of **9** in C_6D_6 at 298 K.

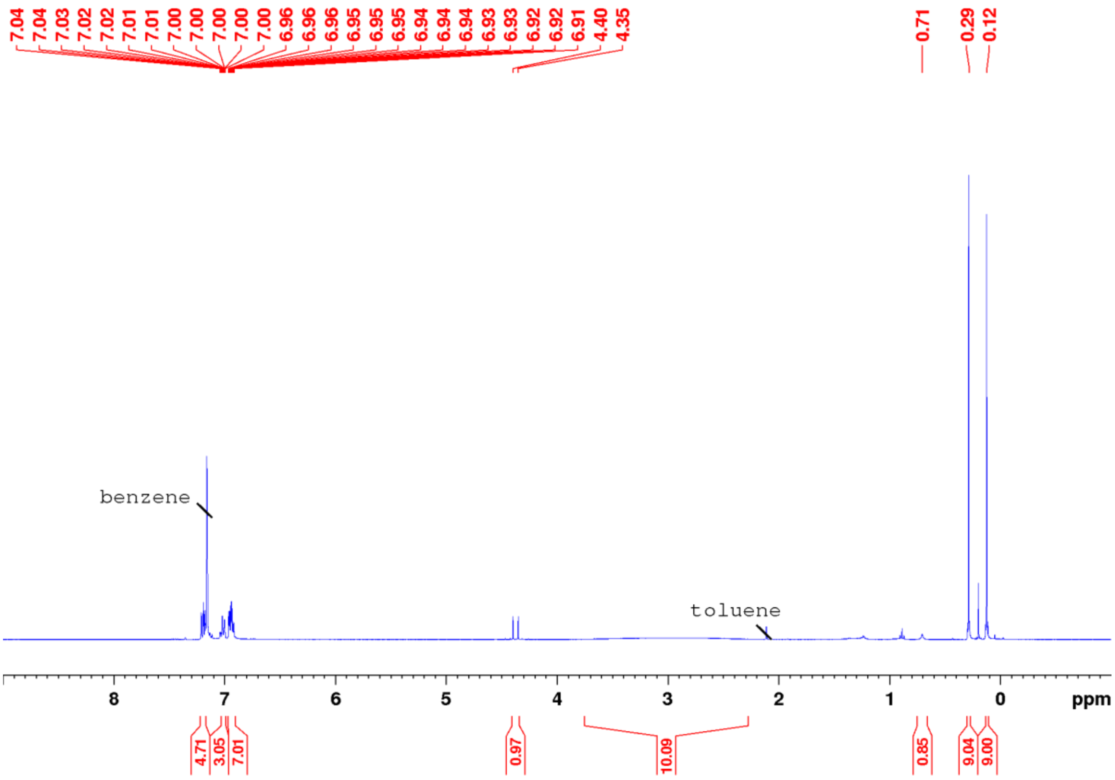

Figure S52. ${}^{1}H$ NMR spectra of **10** in C_6D_6 at 298 K.

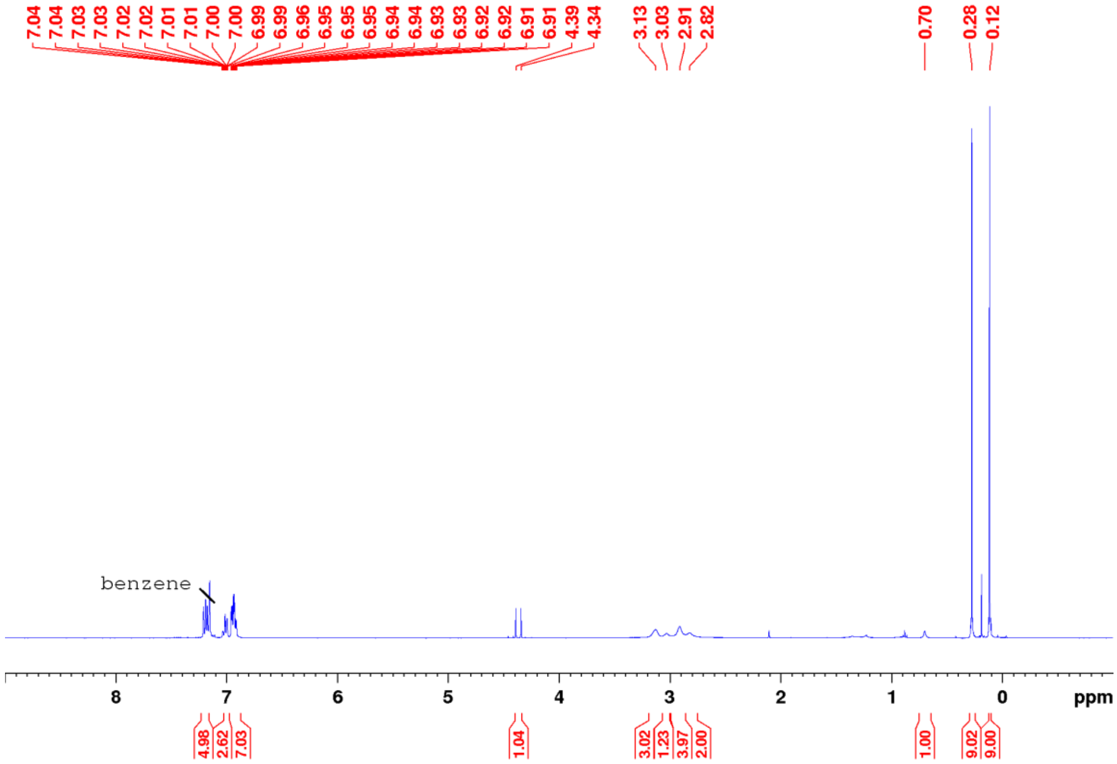

Figure S53. $^{1}H\{^{11}B\}$ NMR spectra of **10** in C_6D_6 at 298 K.

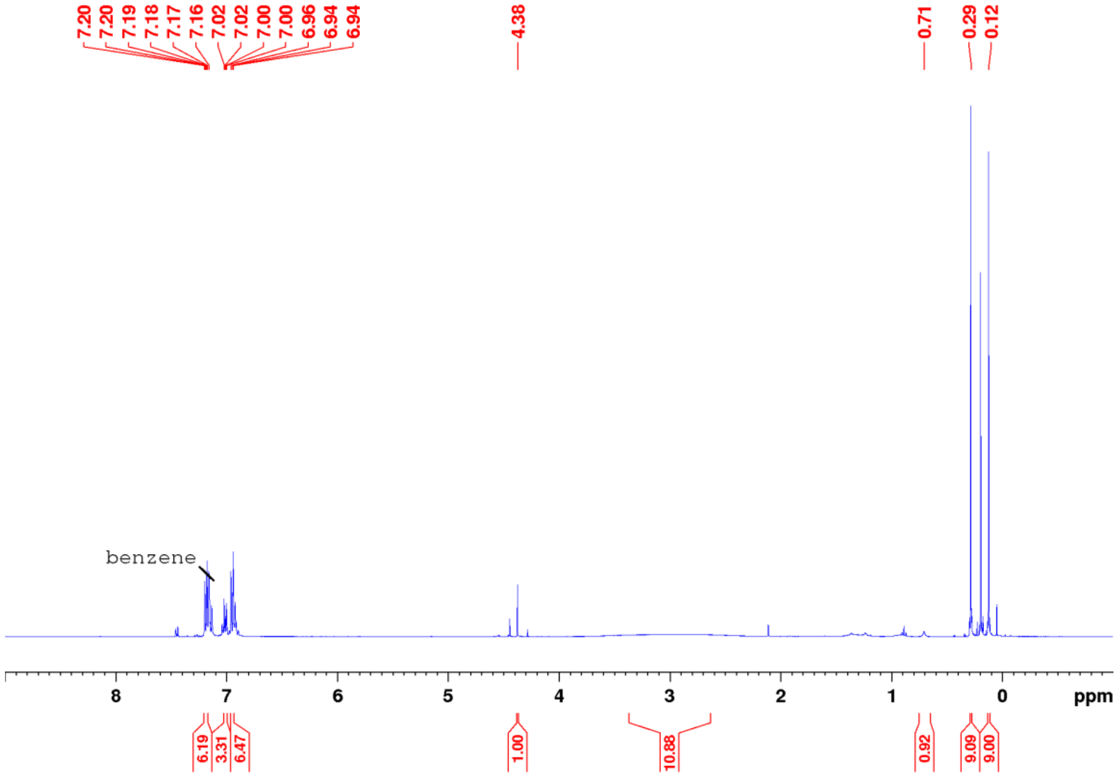

Figure S54. $^{1}H\{^{31}P\}$ NMR spectra of **10** in C_6D_6 at 298 K.

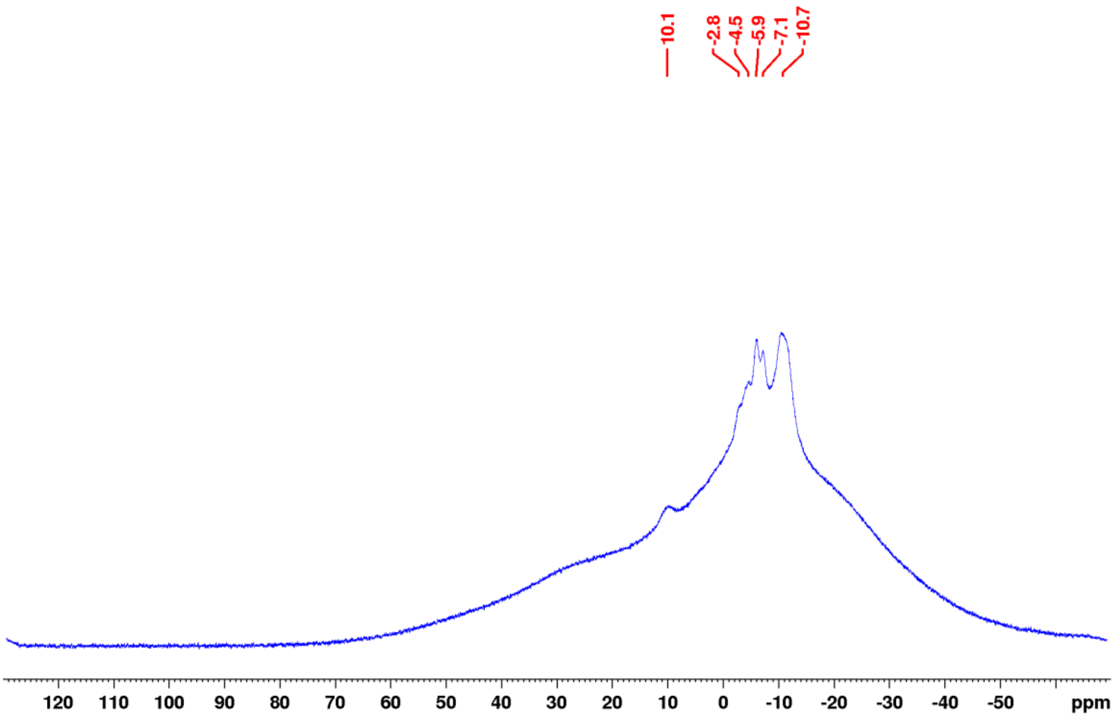

Figure S55. ^{11}B NMR spectra of **10** in C_6D_6 at 298 K.

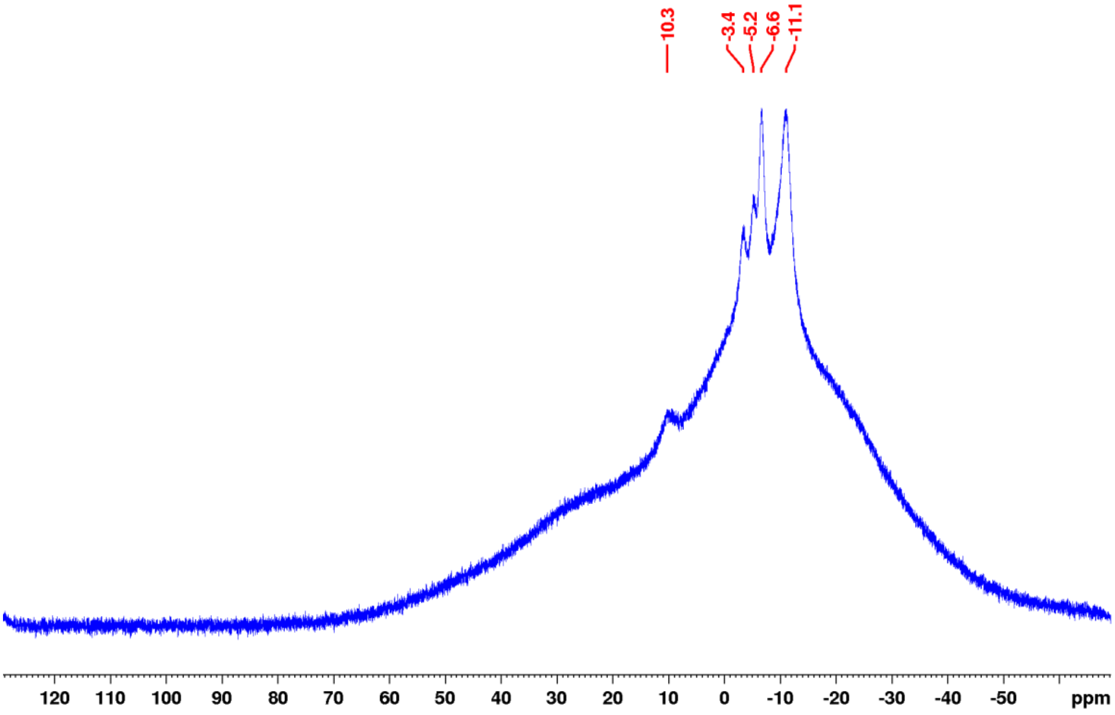

Figure S56. $^{11}B\{^1H\}$ NMR spectra of **10** in C_6D_6 at 298 K.

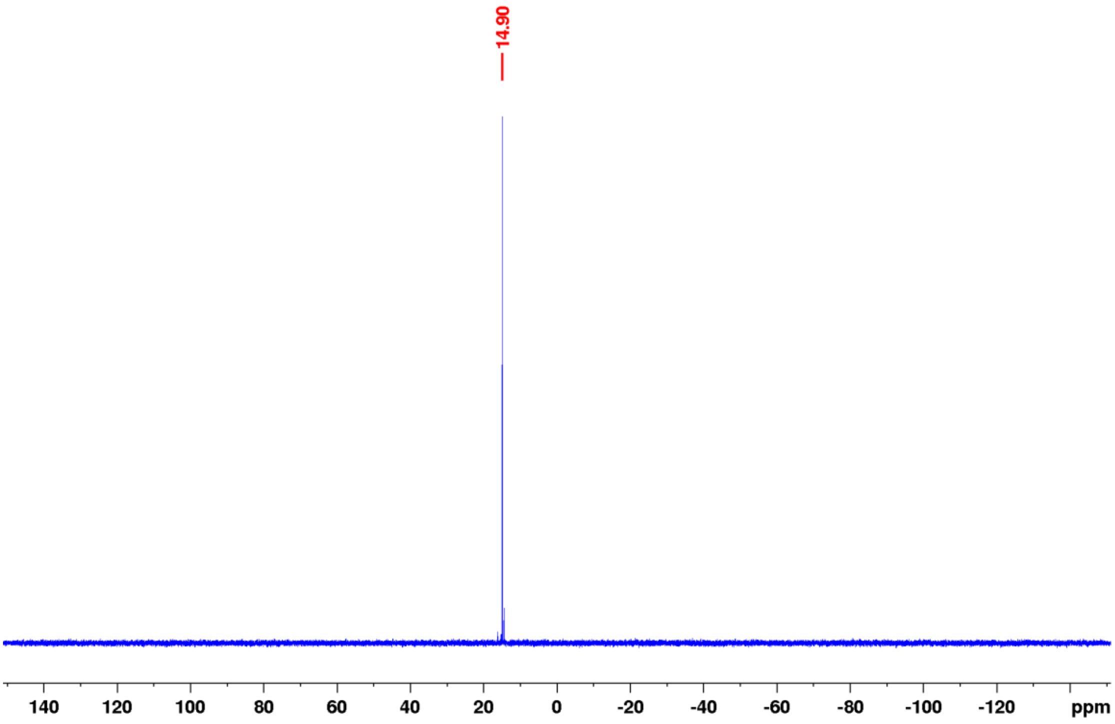

Figure S57. ^{31}P NMR spectra of **10** in C_6D_6 at 298 K.

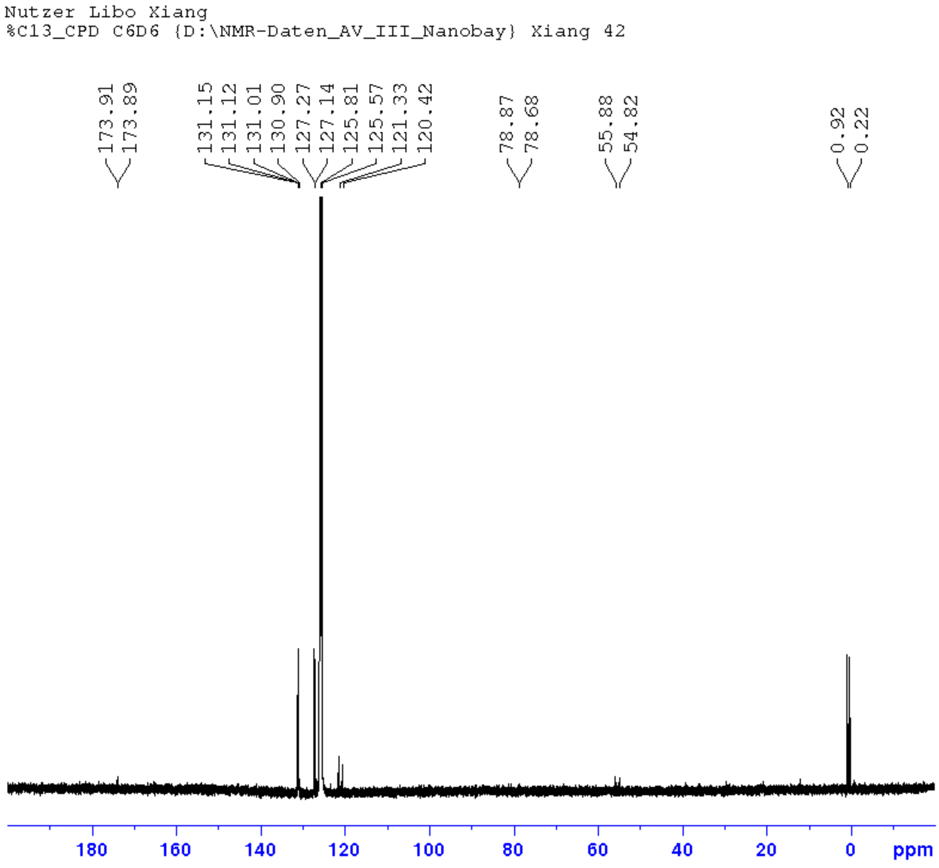

Figure S58. $^{13}C\{^1H\}$ NMR spectra of **10** in C_6D_6 at 298.

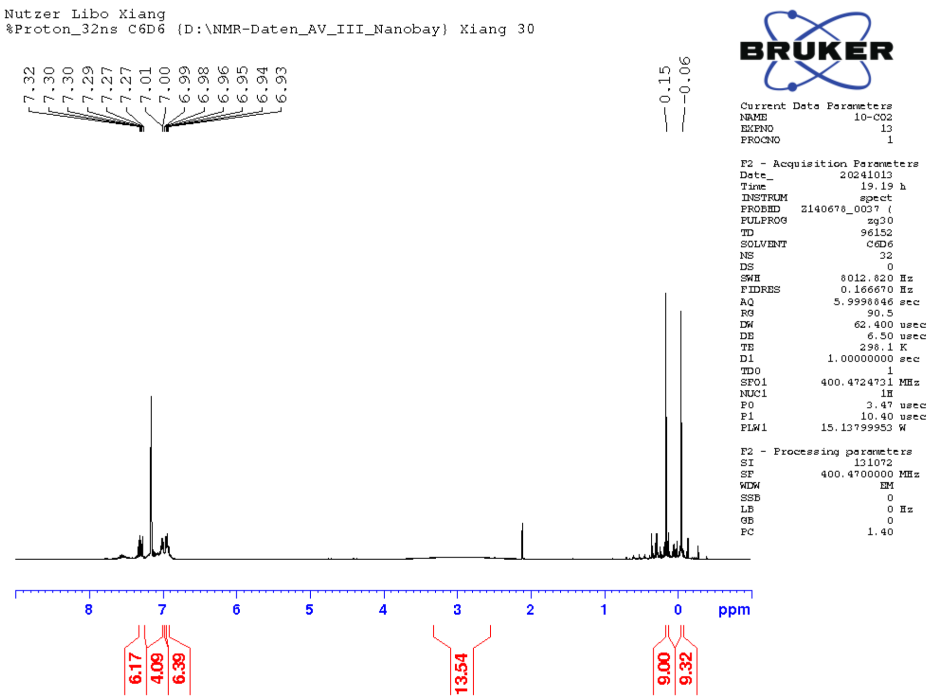

Figure S59. ^{1}H NMR spectra of **11** in C_6D_6 at 298 K.

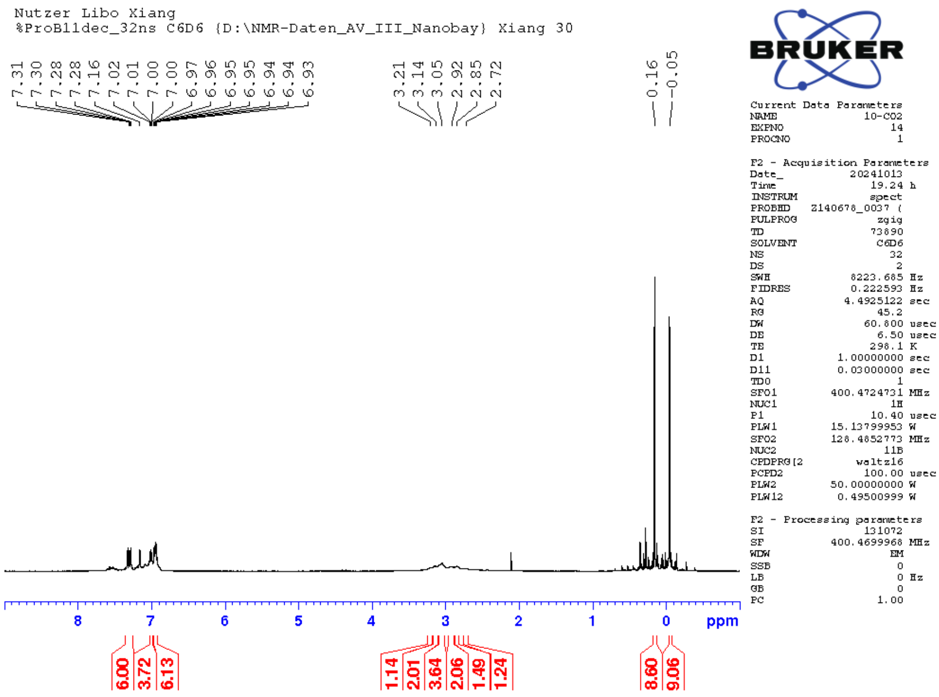

Figure S60. $^{1}H\{^{11}B\}$ NMR spectra of **11** in C_6D_6 at 298 K.

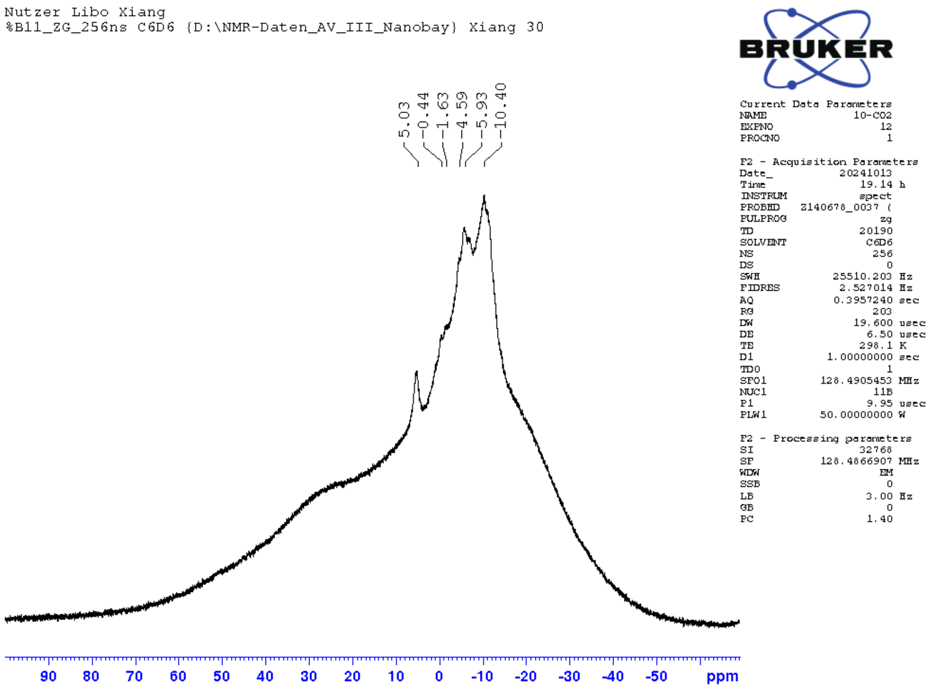

Figure S61. ^{11}B NMR spectra of **11** in C_6D_6 at 298 K.

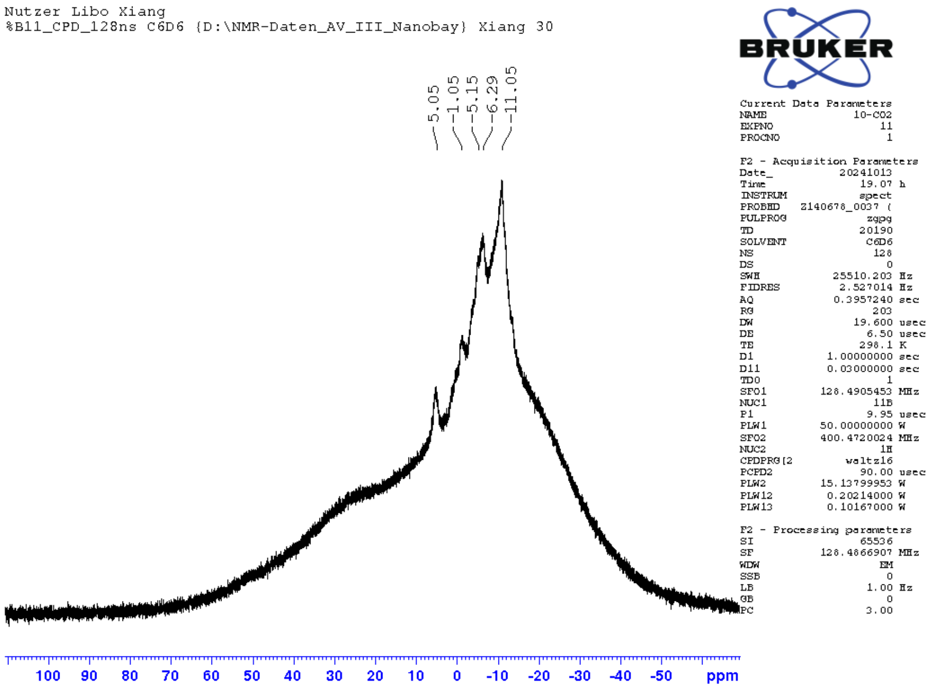

Figure S62. $^{11}B\{^{1}H\}$ NMR spectra of **11** in C_6D_6 at 298 K.

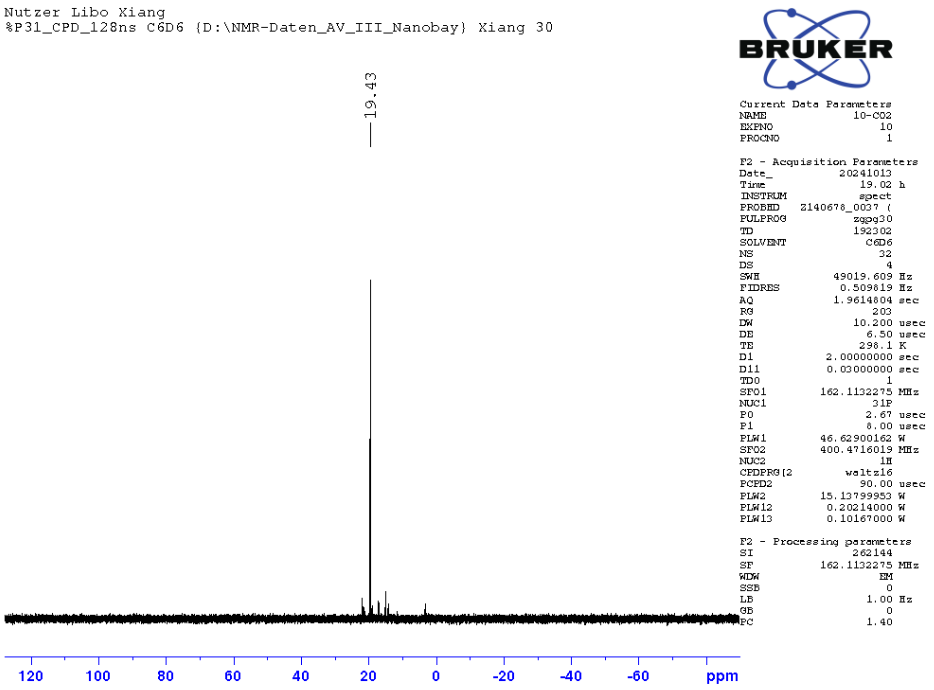

Figure S63. ^{31}P NMR spectra of **11** in C_6D_6 at 298 K.

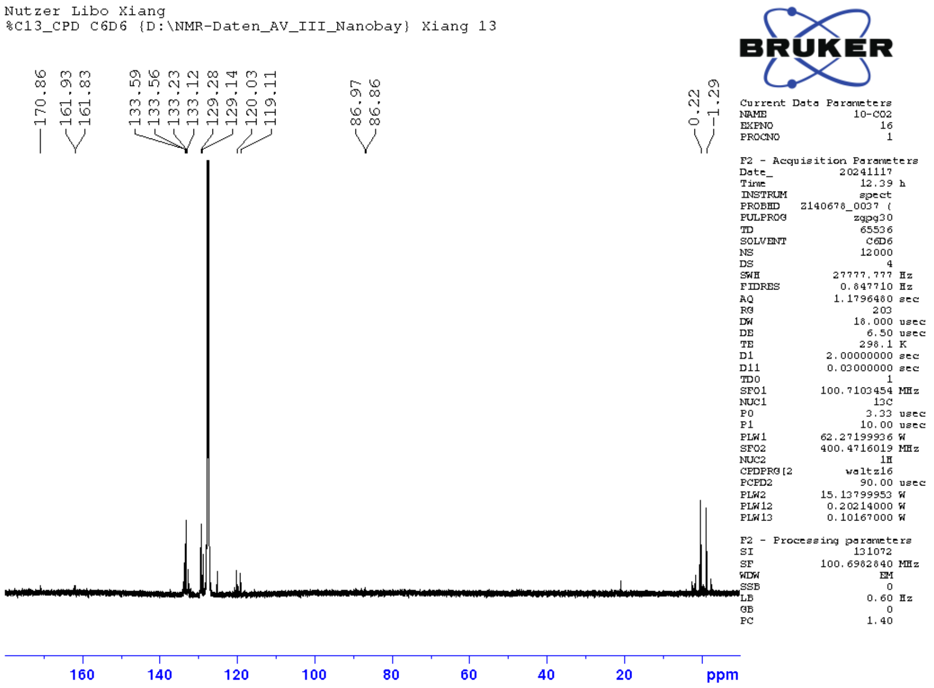

Figure S64. $^{13}C\{^1H\}$ NMR spectra of **11** in C_6D_6 at 298 K.

TEP

Calculation of the TEP from the average carbonyl stretching frequencies of *cis*-[Cl(CO)$_2$Ir-L] complexes:
TEP = 0.847(ν_{avIr}) + 336 cm^{-1}

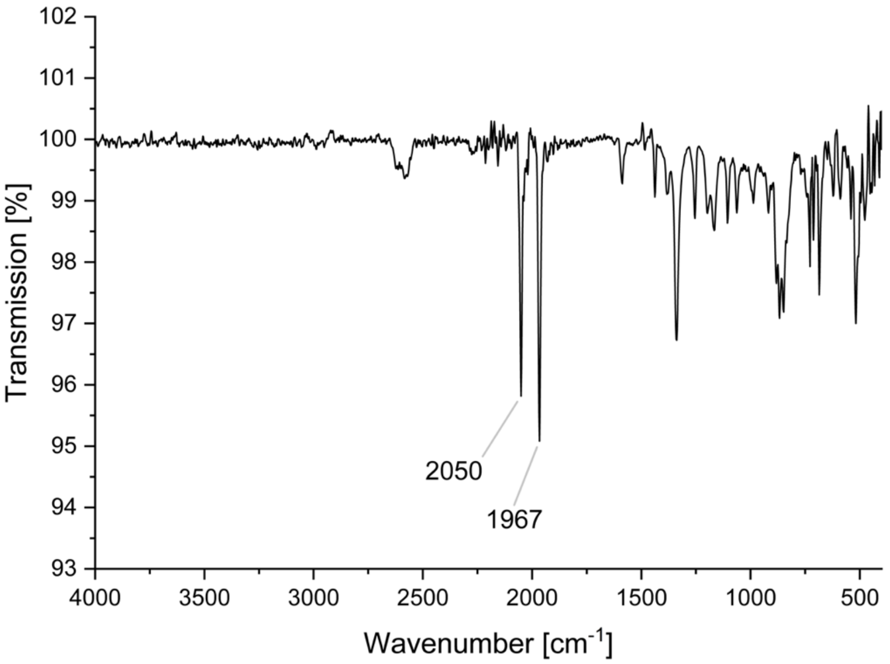

Figure S65. IR spectra of **4**.

Crystallographic Details

The crystal data of **2-4** and **6**, **10**, **11** was collected on a Bruker D8 VENTURE diffractometer with graphite monochromated $Mo_{K\alpha}$ radiation (λ = 0.71073 Å). Data reduction, scaling and absorption corrections were performed using SAINT (Bruker, V8.38A, 2013). The structure was solved with the XT structure solution program using the Intrinsic Phasing solution method[2] and by using Olex2[3] as the graphical interface. The model was refined with the ShelXL program[4] using Least Squares minimization. All non-hydrogen atoms were refined anisotropically. Hydrogen atoms were included in structure factor calculations. All hydrogen atoms were assigned to idealized geometric positions.

The crystal data of **5**, **8**, **7, 9** were collected on a Rigaku XtaLAB Synergy-R diffractometer with a HPA area detector and multi-layer mirror monochromated Cu_{Ka} radiation. The structure was solved using intrinsic phasing method5, refined with the ShelXL program[4] and expanded using Fourier techniques.

Crystallographic data have been deposited with the Cambridge Crystallographic Data Centre, CCDC No. 2418076-2418084, 2425501. These data can be obtained free of charge from The Cambridge Crystallographic Data Centre via Data https://www.ccdc.cam.ac.uk

Details of the data collection and refinement for complexes **2–11** are given in Table S1-S10.

**Table S1.** Crystal data and structure refinement for **2.**	
Empirical formula	$C_{28}H_{43}B_{11}NOPSi_2$
Formula weight	615.69
Temperature/K	99.99
Crystal system	monoclinic
Space group	$P2_1/c$
a/Å	19.1029(8)
b/Å	11.3882(5)
c/Å	17.9433(7)
α/°	90
β/°	104.3850(10)
γ/°	90
Volume/Å^3	3781.1(3)
Z	4
ρ_{calc}g/cm^3	1.082
μ/mm^{-1}	0.159
F(000)	1296.0
Crystal size/mm^3	0.1 × 0.05 × 0.05
Radiation	MoKα (λ = 0.71073)
2Θ range for data collection/°	4.536 to 49.5
Index ranges	-22 ≤ h ≤ 22, -13 ≤ k ≤ 13, -21 ≤ l ≤ 21
Reflections collected	34655
Independent reflections	6462 [R_{int} = 0.0764, R_{sigma} = 0.0506]
Data/restraints/parameters	6462/0/403
Goodness-of-fit on F^2	1.032
Final R indexes [I>=2σ (I)]	R_1 = 0.0456, wR_2 = 0.1127
Final R indexes [all data]	R_1 = 0.0630, wR_2 = 0.1228
Largest diff. peak/hole / e Å^{-3}	0.57/-0.36

**Table S2.** Crystal data and structure refinement for **3.**	
Empirical formula	$C_{36}H_{55}B_{11}ClIrNOPSi_2$
Formula weight	951.52
Temperature/K	100(2)
Crystal system	monoclinic
Space group	$P2_1/c$
a/Å	17.7244(15)
b/Å	11.901(2)
c/Å	21.620(4)
α/°	90
β/°	110.724(10)
γ/°	90
Volume/Å^3	4265.4(12)
Z	4
ρ_{calc}g/cm^3	1.482
μ/mm^{-1}	3.318
F(000)	1912.0
Crystal size/mm^3	0.293 × 0.162 × 0.125
Radiation	MoKα (λ = 0.71073)
2Θ range for data collection/°	3.906 to 54.206
Index ranges	-22 ≤ h ≤ 22, -15 ≤ k ≤ 15, -27 ≤ l ≤ 27
Reflections collected	58591
Independent reflections	9414 [R_{int} = 0.0408, R_{sigma} = 0.0252]
Data/restraints/parameters	9414/600/655
Goodness-of-fit on F^2	1.118
Final R indexes [I>=2σ (I)]	R_1 = 0.0287, wR_2 = 0.0640
Final R indexes [all data]	R_1 = 0.0313, wR_2 = 0.0651
Largest diff. peak/hole / e Å^{-3}	4.34/-1.01

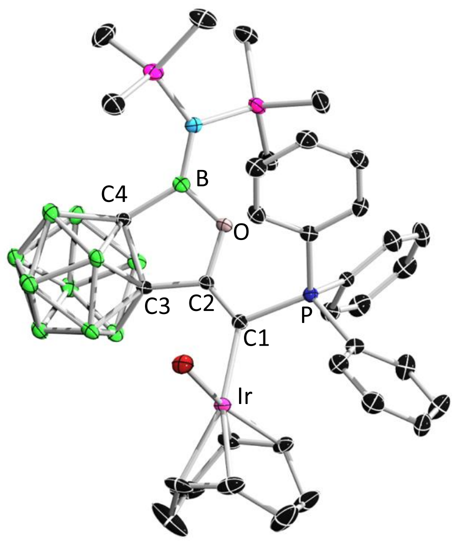

Figure S66. Single crystal structure of **3** in the solid state. The hydrogen atoms were omitted for clarity. Thermal ellipsoids are drawn at the 50% probability level. Selected bond lengths [Å] and angles [°]: C1–C2 1.337(4), C2–C3 1.510(4), C3–C4 1.657(4), C1–P 1.796(3), B–C4 1.601(5), B–O 1.394(4), O–B–C4 106.3(3), P–C1–C2 112.2(2).

**Table S3.** Crystal data and structure refinement for **4.**	
Empirical formula	$C_{30}H_{43}B_{11}ClIrNO_3PSi_2$
Formula weight	899.36
Temperature/K	100.0
Crystal system	monoclinic
Space group	$P2_1/n$
a/Å	17.257(5)
b/Å	12.641(3)
c/Å	19.864(8)
α/°	90
β/°	110.09(2)
γ/°	90
Volume/Å^3	4069(2)
Z	4
ρ_{calc}g/cm^3	1.468
μ/mm^{-1}	3.477
F(000)	1784.0
Crystal size/mm^3	0.293 × 0.15 × 0.105
Radiation	MoKα (λ = 0.71073)
2Θ range for data collection/°	4.206 to 50
Index ranges	-20 ≤ h ≤ 20, -15 ≤ k ≤ 13, -23 ≤ l ≤ 23
Reflections collected	58796
Independent reflections	7157 [R_{int} = 0.0650, R_{sigma} = 0.0334]
Data/restraints/parameters	7157/8/468
Goodness-of-fit on F^2	1.049
Final R indexes [I>=2σ (I)]	R_1 = 0.0420, wR_2 = 0.0969
Final R indexes [all data]	R_1 = 0.0549, wR_2 = 0.1029
Largest diff. peak/hole / e Å^{-3}	1.82/-1.66

**Table S4.** Crystal data and structure refinement for **5.**	
Empirical formula	$C_{28}H_{43}B_{11}NOSi_2PCl_3Ga$
Formula weight	791.76
Temperature/K	100(2)
Crystal system	monoclinic
Space group	$P2_1/n$
a/Å	11.5700(2)
b/Å	16.3152(2)
c/Å	20.5043(3)
α/°	90
β/°	93.7710(10)
γ/°	90
Volume/Å^3	3862.15(10)
Z	4
ρ_{calc}g/cm^3	1.362
μ/mm^{-1}	4.061
F(000)	1624.0
Crystal size/mm^3	0.11 × 0.09 × 0.07
Radiation	CuKα (λ = 1.54184)
2Θ range for data collection/°	6.93 to 150.388
Index ranges	-14 ≤ h ≤ 14, -19 ≤ k ≤ 20, -25 ≤ l ≤ 25
Reflections collected	72657
Independent reflections	7754 [R_{int} = 0.0580, R_{sigma} = 0.0318]
Data/restraints/parameters	7754/0/439
Goodness-of-fit on F^2	1.028
Final R indexes [I>=2σ (I)]	R_1 = 0.0410, wR_2 = 0.0974
Final R indexes [all data]	R_1 = 0.0550, wR_2 = 0.1040
Largest diff. peak/hole / e Å^{-3}	0.85/-0.86

**Table S5.** Crystal data and structure refinement for **6.**	
Empirical formula	$C_{28}H_{46}B_{12}NOPSi_2$
Formula weight	629.53
Temperature/K	100.02
Crystal system	monoclinic
Space group	C2/c
a/Å	40.3619(16)
b/Å	9.2892(4)
c/Å	20.5258(10)
α/°	90
β/°	107.255(2)
γ/°	90
Volume/Å^3	7349.4(6)
Z	8
ρ_{calc}g/cm^3	1.138
μ/mm^{-1}	0.165
F(000)	2656.0
Crystal size/mm^3	0.1 × 0.1 × 0.06
Radiation	MoKα (λ = 0.71073)
2Θ range for data collection/°	4.51 to 49.468
Index ranges	-44 ≤ h ≤ 47, -10 ≤ k ≤ 10, -23 ≤ l ≤ 23
Reflections collected	30216
Independent reflections	6184 [R_{int} = 0.1150, R_{sigma} = 0.0754]
Data/restraints/parameters	6184/0/424
Goodness-of-fit on F^2	1.028
Final R indexes [I>=2σ (I)]	R_1 = 0.0481, wR_2 = 0.1000
Final R indexes [all data]	R_1 = 0.0750, wR_2 = 0.1140
Largest diff. peak/hole / e Å^{-3}	0.29/-0.31

**Table S6.** Crystal data and structure refinement for **7.**	
Empirical formula	$C_{81}H_{100}B_{25}F_{15}N_2O_2P_2Si_4$
Formula weight	1863.17
Temperature/K	100(2)
Crystal system	triclinic
Space group	P1
a/Å	14.5360(4)
b/Å	15.4157(3)
c/Å	23.7127(6)
α/°	79.034(2)
β/°	72.898(2)
γ/°	0.143
Volume/Å^3	4735.8(2)
Z	2
ρ_{calc}g/cm^3	1.307
μ/mm^{-1}	1.539
F(000)	1924
Crystal size/mm^3	0.161 × 0.143 × 0.046
Radiation	Cu_{Ka}, 1.54184
2Θ range for data collection/°	3.074 to 74.805°
Index ranges	-17 ≤ h ≤ 17, -19 ≤ k ≤ 19, -29 ≤ l ≤ 29
Reflections collected	20631
Independent reflections	20631 [R_{int} = 0.0877, R_{sigma} = 0.0251]
Data/restraints/parameters	20631/10/1146
Goodness-of-fit on F^2	1.033
Final R indexes [I>=2σ (I)]	R_1 = 0.1398, wR_2 = 0.3710
Final R indexes [all data]	R_1 = 0.1554, wR_2 = 0.3855
Largest diff. peak/hole / e Å^{-3}	2.162 / –0.816

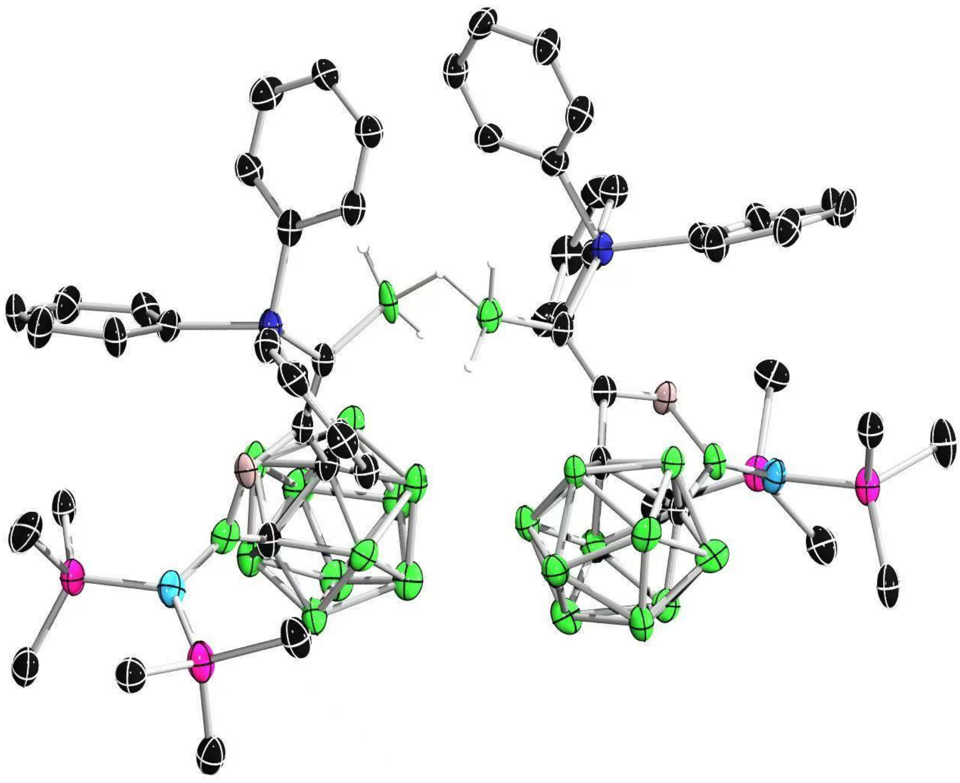

Figure S67. Single crystal structure of **3** in the solid state. The hydrogen atoms and counterion were omitted for clarity. Thermal ellipsoids are drawn at the 30% probability level. Due to the limited crystal quality, this structure is presented solely to depict atomic connectivity, and no discussion of bond lengths is provided.

**Table S7.** Crystal data and structure refinement for **8.**	
Empirical formula	$C_{52}H_{87}B_{13}Cl_2NOPSi_2$
Formula weight	1040.80
Temperature/K	99.99(10)
Crystal system	monoclinic
Space group	C2/c
a/Å	27.1968(16)
b/Å	12.4681(9)
c/Å	39.109(2)
α/°	90
β/°	98.573(5)
γ/°	90
Volume/Å^3	13113.4(14)
Z	8
ρ_{calc}g/cm^3	1.054
μ/mm^{-1}	1.711
F(000)	4448.0
Crystal size/mm^3	0.25 × 0.03 × 0.03
Radiation	CuKα (λ = 1.54184)
2Θ range for data collection/°	6.574 to 149.094
Index ranges	-33 ≤ h ≤ 33, -14 ≤ k ≤ 15, -46 ≤ l ≤ 48
Reflections collected	67928
Independent reflections	13058 [R_{int} = 0.1336, R_{sigma} = 0.0686]
Data/restraints/parameters	13058/89/633
Goodness-of-fit on F^2	1.036
Final R indexes [I>=2σ (I)]	R_1 = 0.1492, wR_2 = 0.2912
Final R indexes [all data]	R_1 = 0.1744, wR_2 = 0.3044
Largest diff. peak/hole / e Å^{-3}	0.51/-0.57

**Table S8.** Crystal data and structure refinement for **9.**	
Empirical formula	$C_{35}H_{52}B_{11}NOFSi_2P$
Formula weight	727.83
Temperature/K	100(2)
Crystal system	monoclinic
Space group	$P2_1/n$
a/Å	14.60710(10)
b/Å	17.37390(10)
c/Å	16.73730(10)
α/°	90
β/°	100.5010(10)
γ/°	90
Volume/Å^3	4176.49(5)
Z	4
ρ_{calc}g/cm^3	1.158
μ/mm^{-1}	1.382
F(000)	1536.0
Crystal size/mm^3	0.24 × 0.2 × 0.13
Radiation	CuKα (λ = 1.54184)
2Θ range for data collection/°	7.396 to 150.05
Index ranges	-17 ≤ h ≤ 17, -20 ≤ k ≤ 20, -20 ≤ l ≤ 18
Reflections collected	43077
Independent reflections	8229 [R_{int} = 0.0236, R_{sigma} = 0.0174]
Data/restraints/parameters	8229/405/541
Goodness-of-fit on F^2	1.058
Final R indexes [I>=2σ (I)]	R_1 = 0.0399, wR_2 = 0.1132
Final R indexes [all data]	R_1 = 0.0433, wR_2 = 0.1156
Largest diff. peak/hole / e Å^{-3}	0.52/-0.33

**Table S9.** Crystal data and structure refinement for **10.**	
Empirical formula	$C_{28}H_{45}B_{11}NO_2Si_2P$
Formula weight	633.71
Temperature/K	100.00
Crystal system	monoclinic
Space group	$P2_1/n$
a/Å	16.776(5)
b/Å	10.808(4)
c/Å	20.752(6)
α/°	90
β/°	102.961(14)
γ/°	90
Volume/Å^3	3667(2)
Z	4
ρ_{calc}g/cm^3	1.148
μ/mm^{-1}	0.168
F(000)	1336.0
Crystal size/mm^3	0.233 × 0.122 × 0.106
Radiation	MoKα (λ = 0.71073)
2Θ range for data collection/°	4.028 to 54.968
Index ranges	-21 ≤ h ≤ 20, -12 ≤ k ≤ 14, -25 ≤ l ≤ 26
Reflections collected	57234
Independent reflections	8392 [R_{int} = 0.0945, R_{sigma} = 0.0622]
Data/restraints/parameters	8392/0/413
Goodness-of-fit on F^2	1.047
Final R indexes [I>=2σ (I)]	R_1 = 0.0643, wR_2 = 0.1446
Final R indexes [all data]	R_1 = 0.0998, wR_2 = 0.1662
Largest diff. peak/hole / e Å^{-3}	0.69/-0.38

**Table S10.** Crystal data and structure refinement for **11.**	
Empirical formula	$C_{30}H_{43}B_{11}NO_5Si_2P$
Formula weight	703.891
Temperature/K	100.00
Crystal system	triclinic
Space group	P-1
a/Å	10.665(3)
b/Å	11.673(4)
c/Å	17.419(6)
α/°	109.52(1)
β/°	96.232(10)
γ/°	106.402(8)
Volume/Å^3	1910.8(11)
Z	2
ρ_{calc}g/cm^3	1.223
μ/mm^{-1}	0.173
F(000)	736.9
Crystal size/mm^3	0.226 × 0.186 × 0.184
Radiation	Mo Kα (λ = 0.71073)
2Θ range for data collection/°	3.94 to 53.46
Index ranges	-13 ≤ h ≤ 13, -15 ≤ k ≤ 15, -22 ≤ l ≤ 22
Reflections collected	47423
Independent reflections	8067 [R_{int} = 0.0492, R_{sigma} = 0.0372]
Data/restraints/parameters	8067/0/457
Goodness-of-fit on F^2	1.052
Final R indexes [I>=2σ (I)]	R_1 = 0.0745, wR_2 = 0.2082
Final R indexes [all data]	R_1 = 0.0842, wR_2 = 0.2164
Largest diff. peak/hole / e Å^{-3}	1.05/-0.46

Calculation details

All calculations were performed with the Gaussian 09 program.[5] Ground-state geometries were optimized gas phase with the BP86 hybrid functional[6] in combination with the def2-SVP basis set.[7] Frequency calculations were carried out to confirm that a local minimum exhibited no imaginary frequencies. The first and second proton affinities were calculated at the same level of theory, and the standard state for all thermodynamic data was 298.15 K and 1 atm. Orbital localization analyses were performed using the Multiwfn program,[8] while buried volume calculations for the optimized $GaCl_3$ adducts were executed utilizing the SEQCROW toolshed within ChimeraX. The natural population analysis and Wiberg bond index were calculated using NBO 7.0.[9]

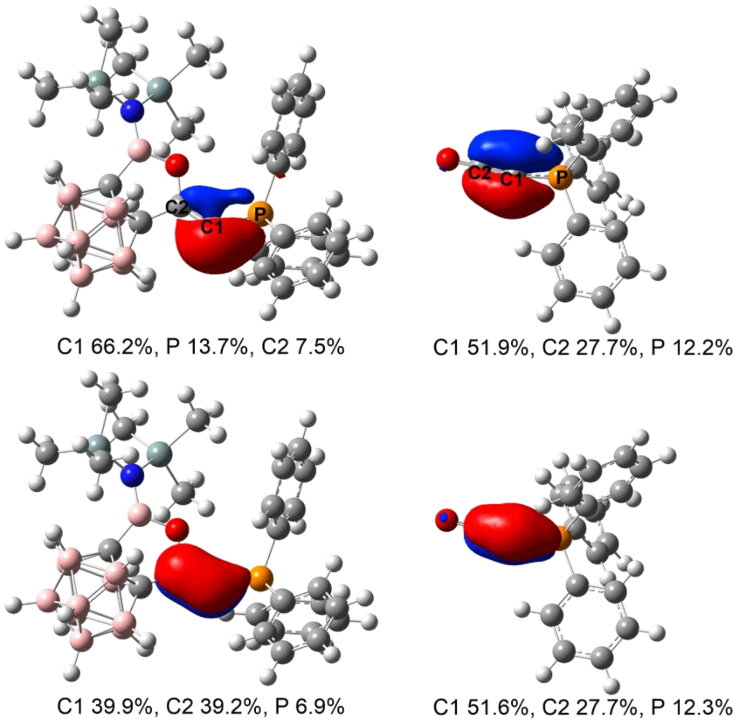

Figure S68. Selected localized molecular orbitals and their atom contributions of compound **2** (left) and PhPCCO (right). Isovalue = 0.05.

Table S11. WBI and NPA charge around central carbon of compound **2** and other CPCs.

$R_xC2{=}C1{=}PR_3$	Wiberg Bond Index		NPA Charge		
	C1-C2	C1-P	C1	C2	P
**2**	1.887	1.193	-0.694	0.298	1.456
Ph_3PCCO	1.954	1.223	-0.949	0.603	1.495
**A**	1.720	1.247	-0.930	0.649	1.463
**B**	1.780	1.038	-0.650	-0.148	1.799
**C**	1.623	1.352	-0.951	0.546	1.453
**D**	1.745	1.251	-0.610	-0.054	1.415
**E**	1.612	1.335	-0.915	0.397	1.453

References

1 (a) H. Zhang, J. Wang, W. Yang, L. Xiang, W. Sun, W. Ming, Y. Li, Z. Lin, Q. Ye, *J. Am. Chem. Soc*. **2020**, *142*, 17243−17249; (b) J. Wang, P. Jia, W. Sun, Y. Wei, Z. Lin, Q. Ye, *Inorg. Chem*. **2022**, *61*, 8879−8886.

2 G. M. Sheldrick, SHELXT - Integrated space-group and crystal-structure determination. *Acta Cryst. A* **2015**, *71*, 3-8.

3 O. V. Dolomanov, L. J. Bourhis, R. J. Gildea, J. A. K. Howard, H. Puschmann, OLEX2: a complete structure solution, refinement and analysis program. *J. Appl. Cryst.* **2009**, *42*, 339–341.

4 G. M. Sheldrick, A short history of SHELX. *Acta Cryst. A* **2008**, *64*, 112-122.

5 M. J. Frisch, G. W. Trucks, H. B. Schlegel, G. E. Scuseria, M. A. Robb, J. R. Cheeseman, G. Scalmani, V. Barone, B. Mennucci, G. A. Petersson, H. Nakatsuji, M. Caricato, X. Li, H. P. Hratchian, A. F. Izmaylov, J. Bloino, G. Zheng, J. L. Sonnenberg, M. Hada, M. Ehara, K. Toyota, R. Fukuda, J. Hasegawa, M. Ishida, T. Nakajima, Y. Honda, O. Kitao, H. Nakai, T. Vreven, J. A., Jr. Montgomery, J. E. Peralta, F. Ogliaro, M. Bearpark, J. J. Heyd, E. Brothers, K. N. Kudin, V. N. Staroverov, R. Kobayashi, J. Normand, K. Raghavachari, A. Rendell, J. C. Burant, S. S. Iyengar, J. Tomasi, M. Cossi, N. Rega, N. J. Millam, M. Klene, J. E. Knox, J. B. Cross, V. Bakken, C. Adamo, J. Jaramillo, R. Gomperts, R. E. Stratmann, O. Yazyev, A. J. Austin, R. Cammi, C. Pomelli, J. W. Ochterski, R. L. Martin, K. Morokuma, V. G. Zakrzewski, G. A. Voth, P. Salvador, J. J. Dannenberg, S. Dapprich, A. D. Daniels, Ö. Farkas, J. B. Foresman, J. V. Ortiz, J. Cioslowski, D. J. Fox, Gaussian 09, Revision D.01; Gaussian, Inc.: Wallingford CT, **2013**.

6 a) A. D. Becke, *Phys. Rev. A* **1988**, *38*, 3098-3100; b) J. P. Perdew, *Phys. Rev. B* **1986**, *33*, 8822-8824

7 F. Weigend R. Ahlrichs, *Phys. Chem. Chem. Phys*. **2005**, *7*, 3297-3305.

8 a) T. Lu, F. Chen, *J. Comput. Chem.* **2012**, *33*, 580-592; b) T. Lu, *J. Chem. Phys*. **2024**, *161*, 082503.

9 NBO 7.0. E. D. Glendening, J. K. Badenhoop, A. E. Reed, J. E. Carpenter, J. A. Bohmann, C. M. Morales, P. Karafiloglou, C. R. Landis, and F. Weinhold, Theoretical Chemistry Institute, University of Wisconsin, Madison, WI (2018).
